# Supplementary material for: GIP/GLP-1RA as adjunctive to automated insulin delivery in adults with Type 1 diabetes (the AID-JUNCT trial): Study protocol for a prospective, randomized, clinical trial
Source: PLoS One. 2025 Oct 22;20(10):e0335060. doi: 10.1371/journal.pone.0335060 (PMC12543135; doi:10.1371/journal.pone.0335060)
Supplement: S2 File — (DOCX) [file pone.0335060.s002.docx]

Anfrage zur Teilnahme an medizinischer Forschung

Studientitel:

GIP/GLP-1RA als Ergänzung zur automatischen Insulinabgabe bei Erwachsenen mit Typ-1-Diabetes: Eine Prospektive, randomisierte, klinische Studie - AID-JUNCT-Studie

Laienverständlicher Titel:

Verwendung zusätzlicher Hormontherapie zur Ergänzung der automatischen Insulinabgabe bei Erwachsenen mit Typ-1-Diabetes: Eine auf noch nicht bereits erhobenen Daten basierende, randomisierte klinische Studie - AID-JUNCT-Studie

Sehr geehrte Dame, sehr geehrter Herr

Hiermit möchten wir Sie über die AID-JUNCT Studie informieren und Sie anfragen, ob Sie daran teilnehmen möchten. Denn, bevor ein Medikament in einem neuen Anwendungsbereich von Ärztinnen und Ärzten verschrieben werden darf, muss untersucht werden, wie dieses Medikament im neuen Anwendungsbereich wirkt.

Eine solche Forschung nennen wir eine **klinische Studie^[[1]](#footnote-2)^**. In dieser Studie wollen wir herausfinden, welchen Effekt das Studienmedikament Mounjaro® (enthaltener Wirkstoff ist Tirzepatid) unterstützend zu einer automatischen Insulinabgabe bei einer Erkrankung mit Diabetes Mellitus Typ-1 auf die Blutzuckerkontrolle hat. Sie leiden an Diabetes Mellitus Typ-1, sind bereits in Behandlung deswegen und verwenden ein automatisches Insulinabgabesystem. Deshalb fragen wir Sie an, ob Sie an dieser Studie teilnehmen möchten.

Ihre Teilnahme ist freiwillig. Die folgende **Patienteninformation** soll Ihnen bei der Entscheidung helfen. Alle Fragen zur Studienteilnahme können Sie im Gespräch mit dem Prüfarzt stellen. So nennen wir die Ärztinnen und Ärzte, die für eine Studie verantwortlich sind und die Sie im Rahmen dieser Studie betreuen. Wenn Sie teilnehmen möchten, unterzeichnen Sie bitte am Ende die **Einwilligungserklärung**. Mit Ihrer Unterschrift bestätigen Sie, dass Sie die Patienteninformation gelesen und verstanden haben. Wenn Sie etwas nicht verstehen, fragen Sie bitte den Prüfarzt.

Die Patienteninformation und Einwilligungserklärung bestehen aus vier Teilen:

Teil 1 Das Wichtigste in Kürze
Teil 2 Darum geht es im Detail: Informationen zur Studie
Teil 3 Datenschutz und Versicherungsschutz
Teil 4 Einwilligungserklärung

Wenn Sie **Teil 1** lesen, dann erhalten Sie einen Überblick über die Studie.
In **Teil 2** erklären wir Ihnen den ganzen Ablauf und Hintergrund der Studie im Detail.
**Teil 3** enthält die Informationen zum Daten- und Versicherungsschutz.
Mit Ihrer Unterschrift am Ende des Dokuments, **Teil 4**, bestätigen Sie, dass Sie alles verstanden haben und mit der Teilnahme einverstanden sind.

Diese Studie wird von der Universität Bern veranlasst. Diese Institution nennt man den Sponsor. Der Sponsor verantwortet, leitet und finanziert eine Studie.

Im Rahmen dieser Studie ist für Sie zuständig:

Name PD Dr. med. Thomas Züger
Adresse Baslerstrasse 150; CH-4600 Olten
Telefon 062 311 44 94

062 311 41 11 (24-Stunden-Erreichbarkeit; Dienstarzt Endokrinologie verlangen)

E-Mail thomas.zueger@spital.so.ch

Teil 1:
Das Wichtigste in Kürze

Vor allem ab Phase-3-Studien (IMP) oder konfirmatorischen MD-Studien.

# Warum führen wir diese Studie durch?

Sie leiden an Diabetes Mellitus Typ-1 und nutzen bereits ein System zur automatischen Insulinabgabe. Deswegen fragen wir Sie hier an, ob Sie an dieser Studie teilnehmen möchten.

Den Blutzuckerwert zu überwachen ist für Menschen, die mit Typ-1-Diabetes (T1D) leben, von grosser Wichtigkeit und stellt immer noch eine Herausforderung dar.

Neue Therapien mit Medikamenten, welche die Insulinausschüttung und Insulinwirkung verbessern zeigen vielversprechende Ergebnisse bei der Verbesserung der Blutzuckerkontrolle und der Reduktion des Insulinbedarfs.

In dieser Studie untersuchen wir, wie sich das Studienmedikament Mounjaro® (Wirkstoff Tirzepatid) in Kombination mit automatischen Insulinabgabesystemen auf die Blutzuckerkontrolle auswirkt und ob es gut verträglich und sicher in dieser kombinierten Anwendung ist. In **Kapitel 4** erfahren Sie mehr zum wissenschaftlichen Hintergrund der Studie.

# Was müssen Sie tun, wenn Sie teilnehmen?

Die Teilnahme an dieser Studie dauert für Sie ungefähr 4,5 – 5 Monate. Wir werden Sie für insgesamt vier Studienvisiten einladen und Sie zwischen den Visiten zwei Mal telefonisch kontaktieren. Falls Sie sich in der Gruppe befinden, welche das Studienmedikament einnehmen wird (Versuchsgruppe), werden wir Sie zusätzlich noch ein weiteres Mal telefonisch kontaktieren. Diese Visiten finden unabhängig von Ihrer regulären Behandlung statt, d.h. sie werden ausschliesslich für die Studie durchgeführt. Eine Studienvisite dauert etwa 45 – 90 Minuten und die telefonischen Kontakte ungefähr 15 Minuten. Die Anzahl und Dauer der Termine sind in der Abbildung in Kapitel 5 angegeben.

Wenn Sie sich entscheiden teilzunehmen, werden Sie zufällig einer von zwei Gruppen zugeteilt. Sie gehören entweder zur Versuchsgruppe oder zur Kontrollgruppe. In der Versuchsgruppe erhalten Sie zusätzlich zu ihrer automatisierten Standardinsulintherapie das Studienmedikament Mounjaro®, welches Sie sich gemäss den Vorgaben, selbst unter die Haut spritzen. In der Kontrollgruppe werden Sie Ihre bisherige automatisierte Standardinsulintherapie weiter anwenden, ohne ein Studienmedikament zu erhalten.

In **Kapitel 5** erfahren Sie mehr zum Ablauf und Vorgehen der Studie.

# Welcher Nutzen und welches Risiko sind mit der Teilnahme verbunden?

## Nutzen

Sie haben keinen direkten Nutzen durch die Teilnahme an der Studie. Es ist aber möglich, dass Sie mit Ihrer Teilnahme künftigen Patientinnen und Patienten helfen.

## Risiko

Die geplanten studienbezogenen Interventionen sind mit ähnlichen Risiken verbunden, denen Menschen mit T1D in ihrem täglichen Leben ausgesetzt sind.

Das Studienmedikament ist derzeit in der Schweiz zur Behandlung von T1D nicht zugelassen, jedoch aber für Typ-2-Diabetes (T2D) und Übergewicht. Es kann sein, dass bei der Einnahme Nebenwirkungen auftreten. Bisher sind folgende Risiken und Nebenwirkungen bekannt:

- Übelkeit, Erbrechen, Verstopfung, Bauchschmerzen, Durchfall, Blähungen, Sodbrennen und Verdauungsstörungen
- Überempfindlichkeitsreaktionen, Reaktionen an der Injektionsstelle, wie Hautausschlag, Hautrötung oder Juckreiz

In **Kapitel 6** finden Sie weitere Informationen zu Risiken und Belastungen.

Teil 2:
Darum geht es im Detail: Informationen zur Studie

# Der wissenschaftliche Hintergrund der Studie

## Hintergrund: Warum führen wir diese Studie durch?

Typ-1-Diabetes (T1D) ist eine Autoimmunerkrankung, die zu einem Insulinmangel und einer lebenslang notwendigen Insulintherapie führt. Den Blutzuckerwert zu überwachen ist für Menschen mit T1D von grosser Wichtigkeit und stellt immer noch eine Herausforderung dar. In der Schweiz erreichen nur etwa 32 % der Erwachsenen einen HbA1c-Zielwert von < 7 %, und dies trotz moderner Insulintherapien, genauerer Blutzuckermessung und neuer Technologien wie automatisierten Insulinabgabesystemen. Diese Technologien verbessern zwar die Blutzuckerwerte und verringern Unterzuckerungen, dennoch sind Übergewicht, Fettstoffwechselstörungen und Herz-Kreislauf-Erkrankungen bei Menschen mit T1D weiterhin häufiger.

Ein Zuwachs der Häufigkeit von Übergewicht und Adipositas bei Menschen mit T1D ist erkennbar. Diese Menschen haben meist höhere HbA1c-Werte, ein erhöhtes Körpergewicht und benötigen grössere Insulindosen. Zusätzliche Therapien zur intensivierten Insulintherapie wurden entwickelt, um den Blutzucker besser zu kontrollieren und das Risiko für Gefässkomplikationen zu senken. Zu den vielversprechenden Medikamenten gehören Hormonanaloga wie GLP-1- und GIP/GLP-1-Rezeptoragonisten (RA), die den Blutzuckerspiegel effektiv senken, ohne ein relevantes Risiko für Unterzuckerungen aufzuweisen. Diese Hormonanaloga gleichen natürlichen Hormonen im Körper, die bei der Regulierung des Blutzuckerspiegels und des Appetits eine Rolle spielen. Somit wirken diese Medikamente, indem sie die Wirkung der natürlichen Hormone nachahmen und so den Blutzucker stabilisieren und das Hungergefühl verringern. Studien zeigen, dass die Kombination dieser Medikamente mit Insulin bei Typ-1-Diabetes (T1D) die Blutzuckerkontrolle verbessert, die Insulindosis reduziert und das Körpergewicht verringert.

Wir planen eine klinische Studie, um die Sicherheit und Wirksamkeit von automatischen Insulinabgabesystemen (AID) in Kombination mit Tirzepatid bei Menschen mit T1D zu testen. Tirzepatid ist ein noch relativ neues Medikament, das die Wirkung von zwei natürlichen Hormonen nachahmt (GIP/GLP-1 RA). Die Ergebnisse sollen neue Erkenntnisse über die Nutzung dieser Therapie liefern.

Das Studienmedikament Mounjaro® (enthaltener Wirkstoff ist Tirzepatid) ist in der Schweiz bisher für die Behandlung von T2D und/oder Übergewicht zugelassen, für die Behandlung von T1D jedoch noch nicht. Erst wenn die Wirksamkeit und Sicherheit des Studienmedikamentes wissenschaftlich untersucht und erwiesen ist, kann es in der Schweiz auch zur Behandlung von Meschen mit T1D zugelassen und eingesetzt werden.

## Aufbau der Studie: Wie gehen wir vor?

In unserer Studie werden die Teilnehmenden zufällig in Gruppen eingeteilt. Dies ist wichtig, um verlässliche Ergebnisse der Studie zu erhalten. Man nennt dies Randomisierung. Jede Gruppe bekommt eine andere Behandlung. In unserer Studie gibt es zwei Gruppen:

- **Gruppe 1** (Versuchsgruppe) bekommt das Studienmedikament in einer anfänglichen Dosierung von 2,5 mg pro Woche für die Dauer von 4 Wochen und danach in einer Dosierung von 5 mg pro Woche für die Dauer von 12 Wochen.
- **Gruppe 2** (Kontrollgruppe) wendet während dieser Zeit die gewohnte Standardbehandlung ohne Studienmedikament an.

Die Wahrscheinlichkeit, der Versuchsgruppe oder der Kontrollgruppe zugeteilt zu werden, ist in dieser Studie gleich hoch (Randomisationsverhältnis 1:1).

Durch die Randomisierung können wir besser beurteilen, ob die neue Behandlung wirkt und wie wirksam und sicher das Studienmedikament als Ergänzung zu einer automatischen Insulinabgabe bei Erwachsenen mit T1D ist.

## Regelungen zur wissenschaftlichen Forschung mit Menschen

Wir machen diese Studie so, wie es die Gesetze in der Schweiz vorschreiben (Humanforschungsgesetz, Datenschutzgesetze). Ausserdem beachten wir alle international anerkannten Richtlinien. Die zuständige Ethikkommission und Swissmedic haben die Studie geprüft und bewilligt.

Unsere Studie ist eine nationale Studie. Das heisst, es gibt 42 Teilnehmende in der Schweiz (21 in der Versuchsgruppe und 21 in der Kontrollgruppe).

Eine Beschreibung dieser Studie finden Sie auch auf der Internetseite des Bundesamtes für Gesundheit unter www.kofam.ch unter der SNCTP-Registriernummer SNCTP000006174 oder der BASEC-Nummer 2024-01947.

# Ablauf der Studie

## Was müssen Sie tun, wenn Sie an der Studie teilnehmen?

Die Teilnahme an der Studie ist freiwillig und dauert 4,5 bis 5 Monate. Sie müssen sich an den Ablaufplan halten (🡪 Kapitel 5.2) und auch an alle Vorgaben, die Ihr Prüfarzt macht.

Sie müssen Ihren Prüfarzt informieren,

- wenn sich Ihr Gesundheitszustand ändert, z. B. wenn es Ihnen schlechter geht oder wenn Sie neue Beschwerden haben; dies gilt auch, wenn Sie die Studie vorzeitig abbrechen (🡪 Kapitel 5.3 und 5.4)
- wenn sie eine Frau sind und versuchen schwanger zu werden oder schwanger sind
- wenn Sie im Laufe der Studie andere Medikamente einnehmen (Begleitmedikation)
- wenn eine neue Erkrankung bei Ihnen diagnostiziert wurde oder Sie einen Unfall hatten (medizinischer Zwischenfall)
- wenn Ihr Ketonwert zwischen 1-1.5 mmol/L ist und sie keine Flüssigkeit zu sich nehmen können
- wenn Ihr Ketonwert grösser als 1.5 mmol/L ist –> falls Sie dehydriert sind und keine Flüssigkeiten zu sich nehmen können, sollten sie die Notaufnahme aufsuchen

Sie müssen ausserdem Folgendes beachten:

- Sie müssen während der Teilnahme das Eintreten einer Schwangerschaft wirksam verhüten (🡪 Kapitel 5.5).
- Sie dürfen während der Teilnahme keine Diät halten oder eine Ernährungsumstellung machen.
- Sie werden während der Studiendauer, zusätzlich zu Ihrem bestehenden Sensor, einen kontinuierlichen Glukosesensor (Dexcom G7) tragen und die Daten mit dem Studienteam teilen. Die Daten dieses Glukosesensors werden nicht verblindet, was bedeutet, dass Sie die Daten sehen werden. Diese Daten werden keinen Einfluss auf Ihr gewohntes Diabetesmanagement haben und ausschliesslich für Forschungszwecke genutzt, um die Datenerfassung zu standardisieren und zu harmonisieren. Ihre Entscheidungen zu Ihrem Diabetesmanagement basieren weiterhin wie gewohnt auf den Messungen Ihres persönlichen Sensors, der mit Ihrem automatisierten Insulinabgabesystem verbunden ist.

## Was passiert bei den Terminen?

Im Verlauf Ihrer Teilnahme kommen Sie viermal zu einer Studienvisite (V1 - V4) zu uns und haben zwei kurze Telefonvisiten (T1/T2) mit uns. Wenn sie in die Gruppe mit dem Studienmedikament eingeteilt werden, haben Sie zusätzlich noch eine weitere Telefonvisite (T3) mit uns. Alle Termine werden für die Forschungsstudie durchgeführt und gehören nicht zur allgemeinen Behandlung. Daten aus Ihrer Patientenakte können für die Studie herangezogen werden, insbesondere zur Überprüfung der Einschlusskriterien sowie bei möglichen schwerwiegenden unerwünschten Ereignissen.

Eine Studienvisite bei uns dauert ungefähr 45 – 90 Minuten, eine Telefonvisite ungefähr 15 Minuten. Die Abfolge der Termine ist in der Abbildung weiter unten angegeben.

Bei allen Terminen machen wir Folgendes:

- Wir beantworten Ihre Fragen
- Wir stellen Ihnen Fragen zu Ihrem Gesundheitszustand, zu Ihrer Begleitmedikation und zu allfälligen medizinischen Zwischenfällen
- Wir besprechen das weitere Vorgehen

Bei einzelnen Terminen machen wir ausserdem

- Eine Blutentnahme zur Überprüfung Ihres Langzeitblutzuckers (HbA1c) und Ihrer Organfunktionen (ca. 15 ml Blut; das entspricht ungefähr einem Esslöffel)
- Eine EKG-Untersuchung, das heisst wir bringen Elektroden auf Ihrem Brustkorb an und messen Ihre Herzströme
- Eine Messung der Körperzusammensetzung mit einer Körperanalysewaage
- Eine Messung Ihrer Vitalzeichen (Blutdruck, Puls, Temperatur)
- Eine ultraschallähnliche Untersuchung der Leber, um allfällige Veränderungen im Lebergewebe zu erkennen
- Eine Abfrage Ihrer Zufriedenheit mit der Diabetesbehandlung mithilfe eines Fragebogens

Alle Teilnehmer:innen nehmen an zwei Telefonvisiten (T1/T2) teil. Dabei werden Ihr Befinden, allfällige medizinische Zwischenfälle und Änderungen an Ihrer Begleitmedikation abgefragt, sowie allfällige Fragen geklärt, und das weitere Vorgehen besprochen. Sie werden die Daten Ihrer Insulinpumpe und dem Glukosesensor herunterladen.

Alle Teilnehmer:innen erhalten ein Gerät zur Messung der Ketonwerte, welches bei Überzuckerung (Hyperglykämien) oder bei Krankheit angewendet wird. Sie erhalten eine bebilderte Anleitung dazu. Zudem werden Sie, während der gesamten Studiendauer ein Hypoglykämie-Tagebuch ausfüllen um bedeutsame Unterzuckerungen, dazugehörige Symptome und mögliche Auslöser wie ausgelassene Mahlzeiten, intensivere Bewegung oder andere Ursachen festzuhalten.

Teilnehmer:innen, welche bei der zweiten Studienvisite (V2) in die Gruppe mit dem Studienmedikament eingeteilt werden, werden zusätzlich an einer dritten Telefonvisite (T3), zwei Wochen nach dem Absetzen des Studienmedikaments, teilnehmen und zudem einmal wöchentlich ein Medikationsprotokoll mit Angaben zur Studienmedikamenteneinnahme ausfüllen.

Alle Teilnehmer:innen werden für die Studiendauer, ab Visite 1, die SNAQ Applikation verwenden. Mit der SNAQ App werden Sie während fünf Tagen vor den Visiten V2-V4 Ihre Mahlzeiten dokumentieren. Sie können die Mahlzeiten manuell in die App eingeben oder Fotos davon aufnehmen.

Ausserdem werden alle Teilnehmer:innen während der gesamten Studiendauer einen

Aktivitäts-Tracker (Fitbit sense 2) am Handgelenk tragen, damit folgende Körpermesswerte aufgezeichnet werden können:

- Puls und die Schwankungen zwischen den Herzschlägen
- Sauerstoffsättigung im Blut
- Atemfrequenz
- Stressniveau
- Schlafphasen
- Schritte
- Hauttemperatur

Durch diese Untersuchungen sehen wir, wie wirksam und sicher das Studienmedikament als Ergänzung zu einer automatischen Insulinabgabe bei Erwachsenen mit T1D ist.

Der Ablaufplan auf der nächsten Seite zeigt alle Studientermine. Die **Untersuchungen und Aktivitäten** für alle TeilnehmerInnen sind mit **grau hinterlegtem Haken** **(**
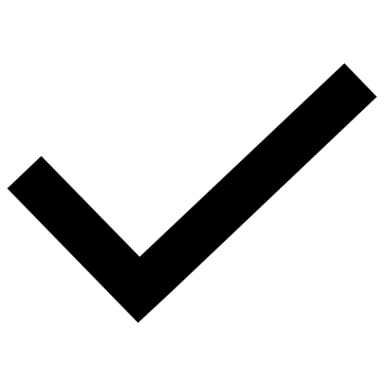
 **)** gekennzeichnet. Die **Aktivitäten**, welche ausschliesslich für **TeilnehmerInnen in der Versuchsgruppe** mit dem Studienmedikament durchgeführt werden, sind **mit Pluszeichen** ( **+** ) gekennzeichnet.

### Ablaufplan: Studienvisiten

| **Studienvisite (V)/ Telefonvisite (T)** | **V 1** | **V 2** | T 1 | **V 3** | T 2 | **V 4** | T 3 |
| --- | --- | --- | --- | --- | --- | --- | --- |
| **Datum** | **0** | **Wo 2** | Wo 6 | **Wo 10** | Wo 14 | **Wo 18** | Wo 20 |
| **Dauer in Minuten** (ungefähre Angaben) | **90** | **60** | 15 | **45** | 15 | **60** | 15 |
| Unterschrift Einwilligungserklärung | 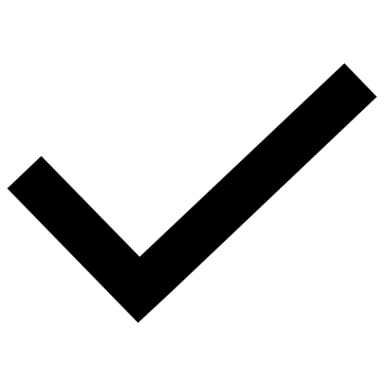 |  |  |  |  |  |  |
| Prüfung der Ein- und Ausschlusskriterien | 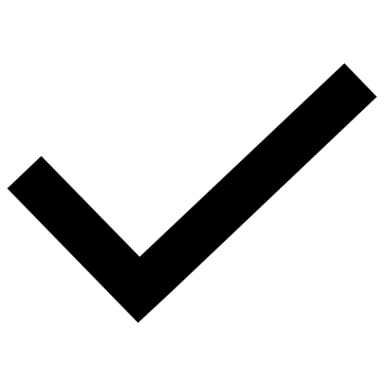 |  |  |  |  |  |  |
| Zuteilung in Versuchs- oder Kontrollgruppe |  | 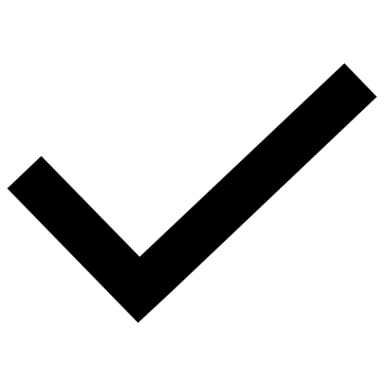 |  |  |  |  |  |
| Überprüfen Ihrer Krankheitsgeschichte, demographischen Daten, Medikamente | 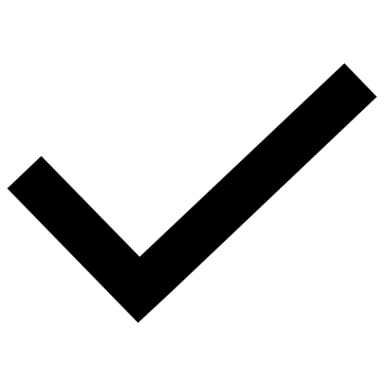 |  |  |  |  |  |  |
| Blutdruck, Puls, Temperatur, Schwangerschaftstest | 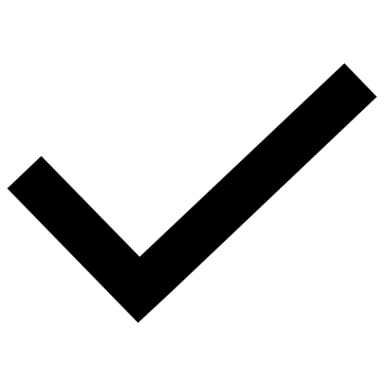 | 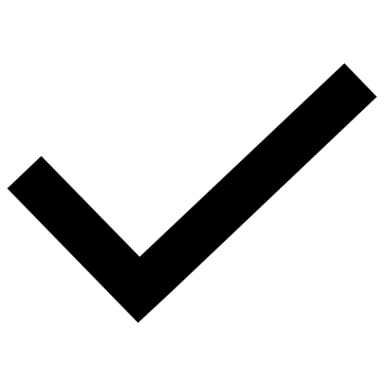 |  | 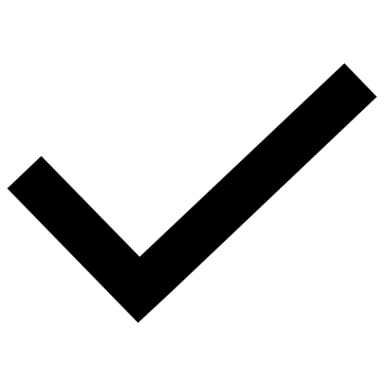 |  | 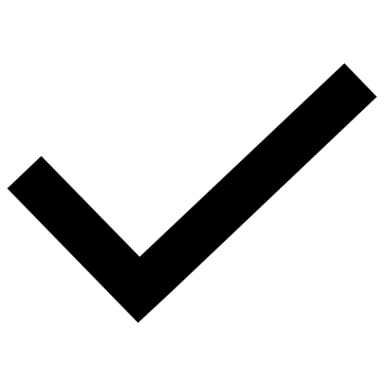 |  |
| Körpergewicht, Body Mass Index, Taillen- und Hüftumfang, EKG | 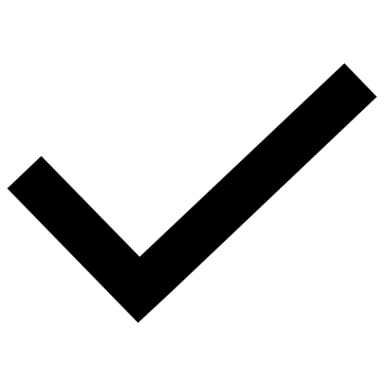 |  |  | 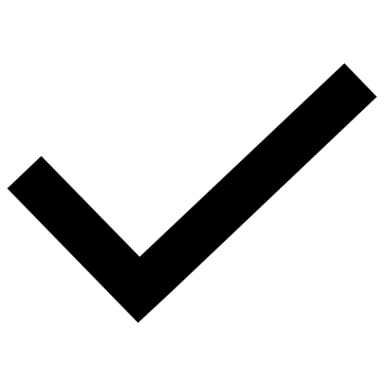 |  | 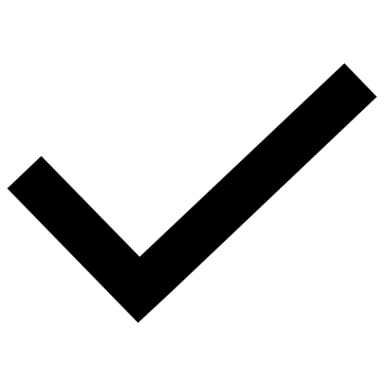 |  |
| Messung Körperzusammensetzung, Ultraschall der Leber |  | 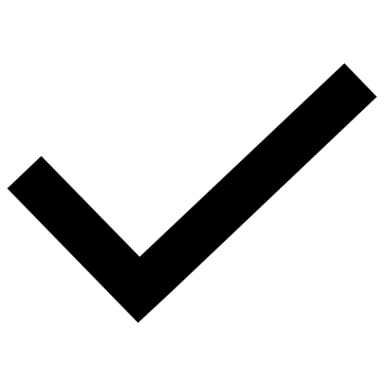 |  |  |  | 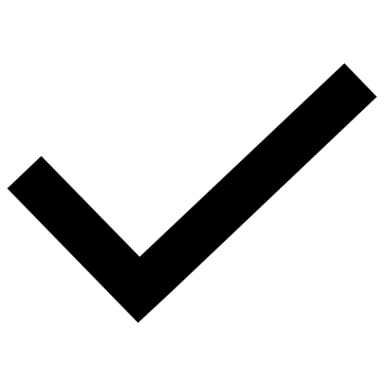 |  |
| Überprüfung Hypoglykämie-Tagebuch |  | 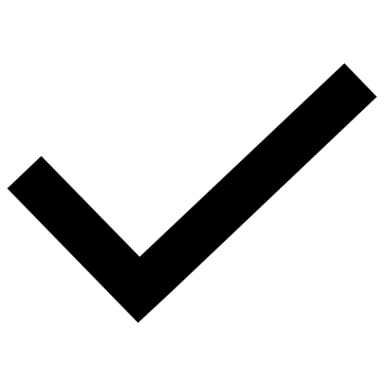 | 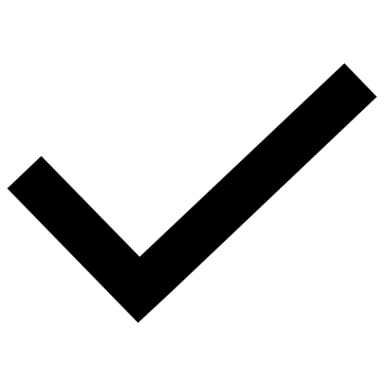 | 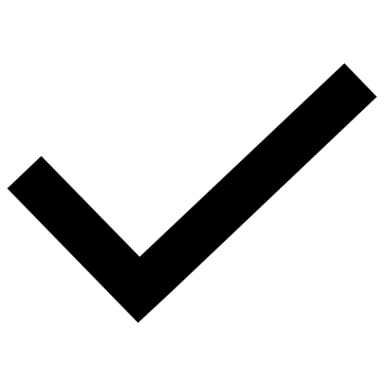 | 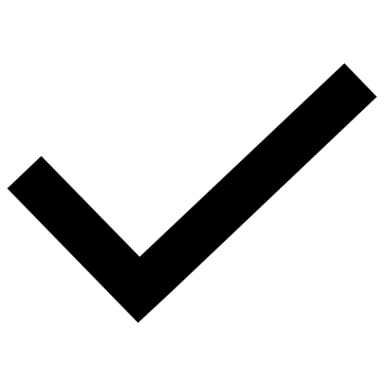 | 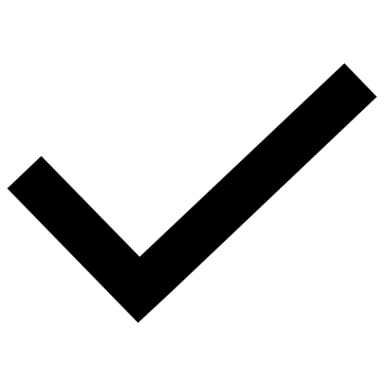 |  |
| Fragebogen |  | 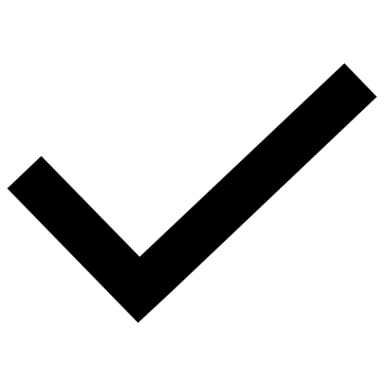 |  |  |  | 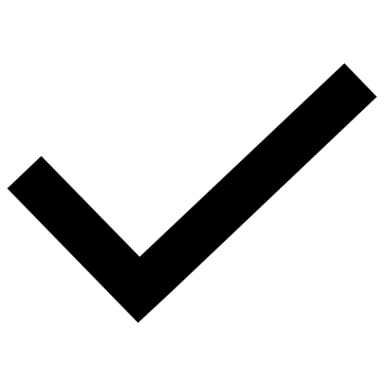 |  |
| Blutentnahme | 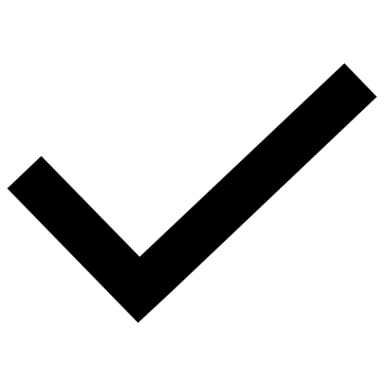 |  |  | 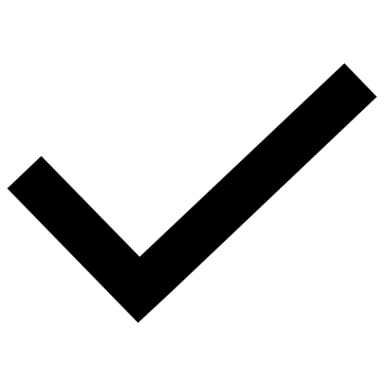 |  | 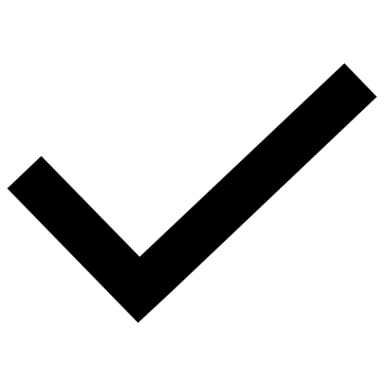 |  |
| Instruktionen / Abgabe / Installation von SNAQ App und Aktivitäts-Tracker | 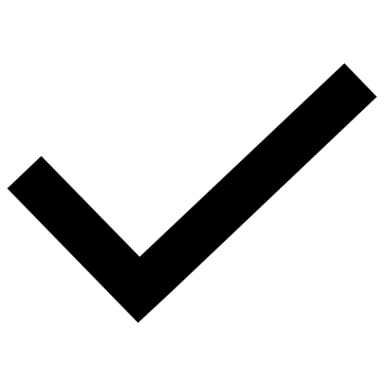 |  |  |  |  |  |  |
| Deinstallation Apps und Aktivitäts-Tracker |  |  |  |  |  | 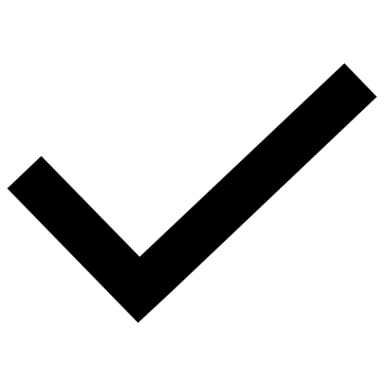 |  |
| Download Daten Glukosemonitor und Insulinpumpe, Fragen zum Blutzucker |  | 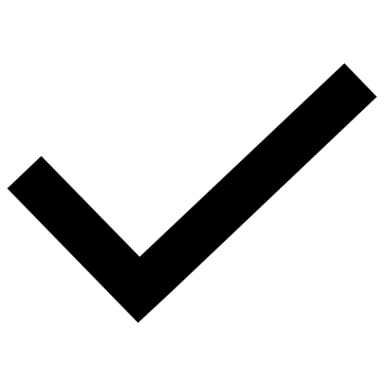 | 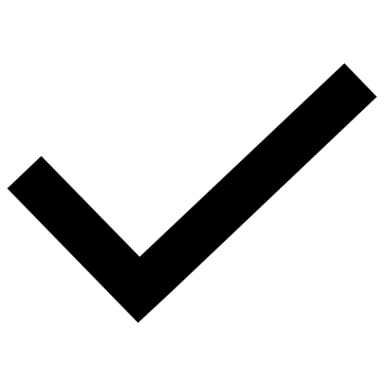 | 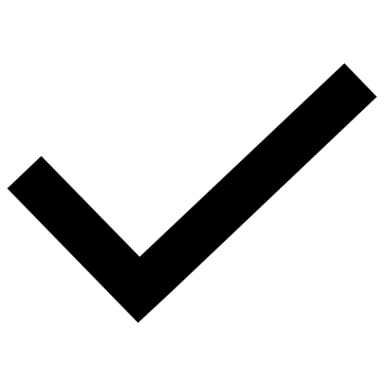 | 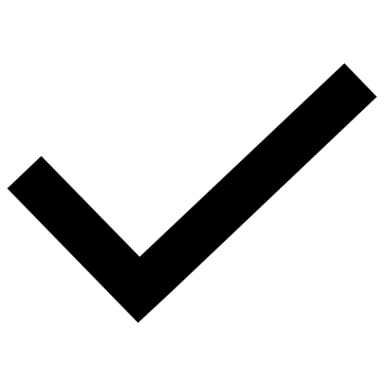 | 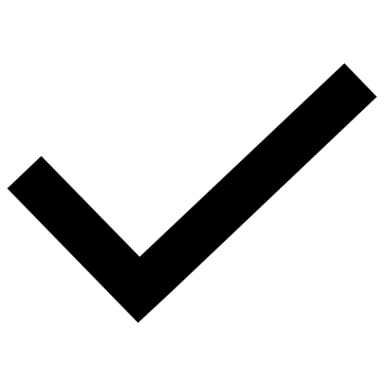 | 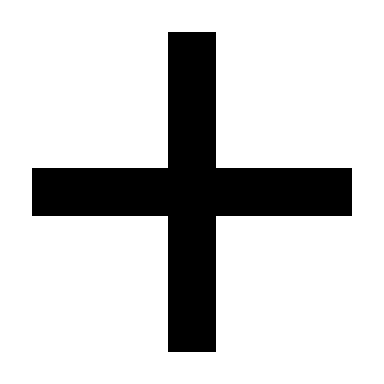 |
| Instruktion und Abgabe Ketonmesser | 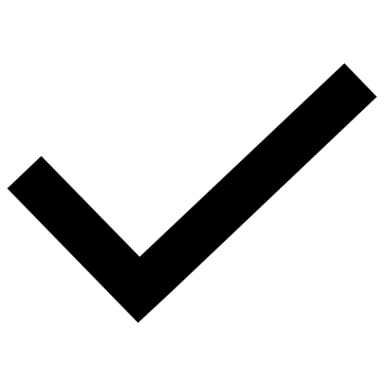 |  |  |  |  |  |  |
| Rückgabe Ketonmesser |  |  |  |  |  | 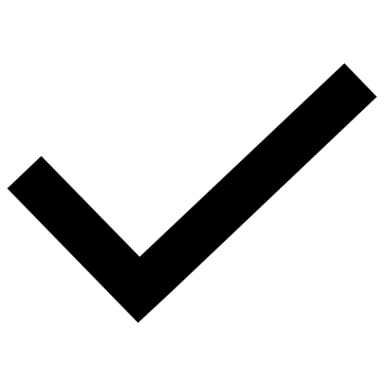 |  |
| Instruktionen/ Informationen Studienmedikament |  | 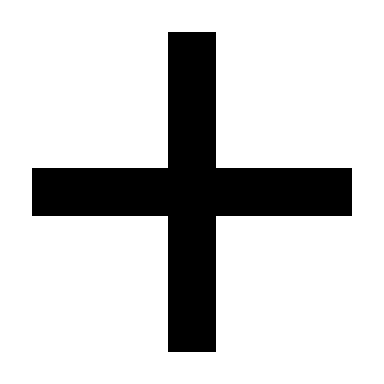 |  |  |  |  |  |
| Abgabe Studienmedikament |  | 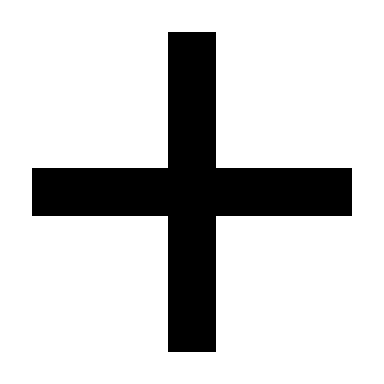 |  | 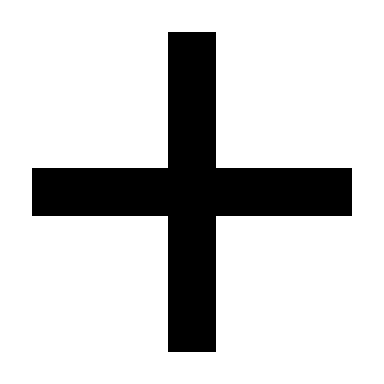 |  |  |  |
| Auswertung Medikationsprotokoll |  |  |  | 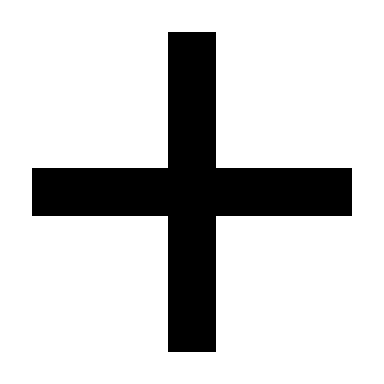 |  | 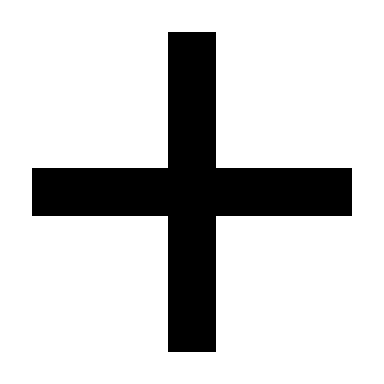 |  |
| Rückgabe Studienmedikament (leere Pens) |  |  |  | 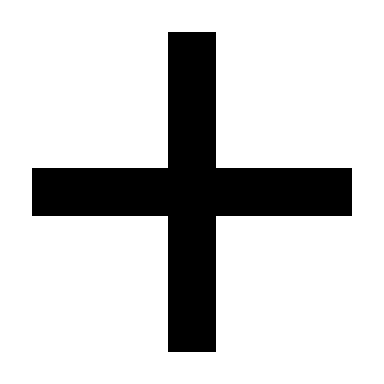 |  | 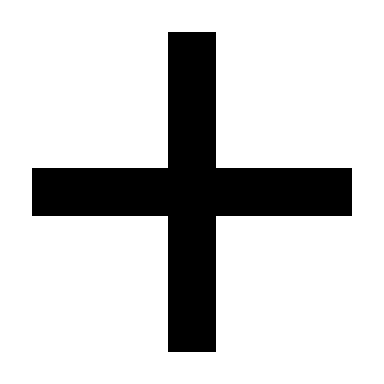 |  |

Wir vereinbaren die Termine gemeinsam mit Ihnen. Sie erhalten einen genauen Überblick über die Termine. Die Studienvisiten können nicht einfach verschoben werden. Wir bitten Sie, uns frühzeitig zu informieren, wenn Sie trotzdem einmal einen Termin aus wichtigen Gründen verschieben müssen oder während der Studienzeit Ferien planen.

## Wann endet die Teilnahme an der Studie?

Reguläre Dauer:

- Für Patienten in der Kontrollgruppe dauert die Teilnahme 4,5 Monate und endet nach der vierten Studienvisite (V4).
- Für Patienten in der Versuchsgruppe dauert die Teilnahme 5 Monate und endet nach der dritten Telefonvisite (T3).

Vorzeitiger Ausstieg:

Sie können Ihre Teilnahme jederzeit auch vorzeitig abbrechen (🡪 Kapitel 5.4). Sie müssen nicht erklären, warum Sie nicht mehr teilnehmen möchten. Wenn Sie Ihre Teilnahme früher beenden möchten, sprechen Sie bitte mit Ihrem Prüfarzt.

Wenn Sie Ihre Teilnahme vorzeitig beenden, hat dies keinen Einfluss auf Ihre weitere medizinische Versorgung und Behandlung (🡪 Kapitel 5.4 für alternative Behandlungsoptionen). In diesem Fall machen wir zu Ihrer Sicherheit abschliessend noch eine Untersuchung. Bitte bringen Sie dann, falls zutreffend, alle übrigen Studienmedikamente, den Ketonmesser, das Studien-Smartphone (falls zutreffend) und den Aktivitäts-Tracker zu uns zurück. Ausserdem werden wir Sie ungefähr 14 Tage nach der Abschlussuntersuchung anrufen, um sicherzustellen, dass sie in einem guten Gesundheitszustand sind.

Wenn Ihre Teilnahme vorzeitig endet, werden wir die bis dahin erhobenen Daten (z.B. Blutwerte oder Glukosemonitor-Daten) noch für die Studie auswerten. Ihre Studiendaten bleiben weiterhin verschlüsselt (🡪 Kapitel 9).

Es kann auch sein, dass wir Sie bitten müssen, die Studie frühzeitig zu beenden. Das ist zum Beispiel der Fall, wenn Sie das Studienmedikament nicht vertragen, wiederholte Unterzuckerungen oder zu viele Ketonkörper im Blut haben.

## Was passiert, wenn Sie nicht teilnehmen möchten?

Auch wenn Sie nicht an dieser Studie teilnehmen, behandeln und betreuen wir Sie medizinisch bestmöglich nach den aktuellen Standards. Wenn Sie nicht an der Studie teilnehmen möchten, wird Sie Ihr Prüfarzt im Gespräch über alternative Behandlungsmöglichkeiten beraten.

## Schwangerschaft

Das Studienmedikament ist für Menschen, die mit Typ-2 Diabetes leben zugelassen. Es kann für ein ungeborenes Kind gefährlich und schädlich sein.

Deswegen dürfen Sie während Ihrer Teilnahme an der Studie nicht schwanger sein, schwanger werden oder ein Kind zeugen. Das gilt für Frauen und Männer, die an dieser Studie teilnehmen. Sie werden diesen Punkt mit Ihrem Prüfarzt besprechen.

### Für Frauen, die schwanger werden können

Sie dürfen während Ihrer Teilnahme an der Studie und vier Wochen nach der letzten Studienvisite (V4) nicht schwanger werden. Sie müssen Ihre(n) Partner darüber informieren, dass Sie an dieser Studie teilnehmen. Vor Beginn der Studie machen wir einen Schwangerschaftstest im Blut. Wir wiederholen den Schwangerschaftstest im Urin während der Studie bei den Visiten V2, V3 und V4. Wenn Sie stillen, dürfen Sie nicht teilnehmen.

Während der Teilnahme an der Studie müssen Sie eine hochwirksame oder zwei wirksame Verhütungsmethoden anwenden.

Hochwirksame Verhütungsmethoden:

Oral:

- Orale Verhütungsmethode mit einer kombinierten (Oestrogen und Gestagen) Pille (Tablette, die den Eisprung unterdrückt) oder Minipille (Tablette, welche die Schleimhaut verändert und bei einigen Präparaten den Eisprung unterdrückt).

Nicht oral:

- Hormoninjektion oder Hormonimplantat («Stäbchen»)
- Eileiterimplantat
- Verhütungspflaster
- Vaginalring oder Intrauterinpessare (Kupfer- oder Hormonspirale)
- Regulärer Verzicht auf Geschlechtsverkehr

Wirksame Verhütungsmethoden:

- Kondome für Frauen oder Männer mit Spermizid (ein chemisches Verhütungsmittel,

welches Spermien abtötet oder ihre Beweglichkeit stark beeinträchtigt)

- Diaphragma mit Spermizid oder Zervixschwamm

Es ist wichtig zu wissen, dass das Studienmedikament die Wirksamkeit der oralen Verhütungsmethode reduzieren kann. Falls sie eine orale Verhütungsmethode verwenden und sich in der Versuchsgruppe befinden, müssen Sie daher während acht Wochen, beginnend ab V2, zusätzlich eine Barriere-Verhütungsmethose verwenden, wie z.B. Kondom oder Diaphragma (idealerweise mit einem Spermizid zur Erhöhung der Sicherheit), oder Sie wechseln von Ihrem oralen Verhütungsmittel auf eine nicht orale Verhütungsmethode.

Sie müssen diese Verhütungsmethoden auch nach dem Ende der Studie (V4) noch vier Wochen anwenden. Wenn Sie im Verlauf der Studie oder bis zu 4 Wochen danach trotzdem schwanger werden, müssen Sie das sofort Ihrem Prüfarzt sagen. Er wird dann mit Ihnen und Ihrem Partner über das weitere Vorgehen sprechen.

### Für Männer

Das Studienmedikament (Mounjaro®) ist für Menschen, die mit T2D leben, zugelassen. Es gibt bisher keine umfassenden Studien, die die Auswirkungen von Mounjaro® auf die Spermienqualität untersucht haben. Sie müssen daher, falls Sie das Studienmedikament einnehmen, Ihre Partnerin(nen) - falls diese im gebärfähigen Alter und nicht schwanger ist - darüber informieren, dass Sie an dieser Studie teilnehmen.

Falls Sie der Versuchsgruppe zugeteilt sind, müssen Sie während der Teilnahme an der Studie und bis zu 4 Wochen nach der letzten Dosis des Studienmedikaments den Verhütungsempfehlungen folgen. Weil das Kondom als alleinige Verhütungsmethode aber nicht sicher genug ist, muss Ihre Partnerin (bzw. müssen Ihre Partnerinnen) zusätzlich eine hochwirksame Verhütungsmethode anwenden (siehe oben). Wenn Ihre Partnerin(nen) keine hochwirksame Verhütungsmethode anwendet, müssen beide Partner eine wirksame Verhütungsmethode anwenden. Falls Ihre Partnerin bereits zu Beginn der Studie schwanger ist, ist das Verwenden von Kondomen ausreichend.

Wenn Ihre Partnerin im Verlauf der Studie oder bis zu 4 Wochen danach trotzdem schwanger wird, müssen Sie das umgehend Ihrem Prüfarzt sagen. Ihr Prüfarzt wird dann mit Ihnen und Ihrer Partnerin über das weitere Vorgehen sprechen.

# Risiken, Belastungen und Nebenwirkungen

## Welche Risiken und Belastungen können auftreten?

Es gibt Risiken und Belastungen bei der Teilnahme an dieser Studie, wie bei jeder medizinischen Behandlung. Manche Risiken kennen wir bereits, andere sind noch unbekannt. Diese Unsicherheit ist im Umfeld von Studien nicht ungewöhnlich. Sie finden in **Kapitel 6.2** eine Liste der häufigsten und schwersten Risiken. Viele Nebenwirkungen sind medizinisch behandelbar. Wir informieren Sie während der Studie über alle neuen Erkenntnisse zu Risiken und Nebenwirkungen.

Bei einer neuen Anwendung eines Medikaments, ist es möglich, dass es Risiken gibt, die wir noch nicht kennen. Das Studienmedikament Mounjaro® ist derzeit in der Schweiz zur Behandlung von T1D nicht zugelassen, jedoch für die Behandlung von Typ-2-Diabetes (T2D) und/oder Übergewicht.

Zusätzlich gibt es Risiken bei den medizinischen Untersuchungen, die wir in dieser Studie machen. Manche Untersuchungen werden Sie bereits kennen. Sie finden in **Kapitel 6.3** eine Liste dieser Risiken der Untersuchungen.

## Die häufigsten und schwerwiegendsten Risiken durch das Studienmedikament

Sie finden hier Informationen über die häufigsten und schwerwiegendsten Nebenwirkungen, die wir bereits kennen und Sie auch gerne in der Fach- oder Patienteninformation des Studienmedikaments Mounjaro® nachlesen dürfen. Diese Informationen sind online und frei zugänglich auf der Webseite [compendium.ch](https://compendium.ch/) verfügbar.

Wir benutzen die folgenden Beschreibungen:

| sehr häufig | Wir finden die Nebenwirkung bei mehr als 10 Personen von 100 (mehr als 10%). |
| --- | --- |
| häufig | Wir finden die Nebenwirkung bei 1 bis 10 Personen von 100 (1%-10%). |
| gelegentlich | Wir finden die Nebenwirkung bei 1 bis 10 Personen von 1‘000 (0.1%-1%). |
| selten | Wir finden die Nebenwirkung bei 1 bis 10 Personen von 10‘000 (0.01%-0.1%). |
| sehr selten | Wir finden die Nebenwirkung bei weniger als 1 Person von 10‘000 (unter 0.01%). |

Sehr häufige Nebenwirkungen sind:

- Übelkeit
- Durchfall
- Verstopfung bei Verwendung zur Gewichtsregulierung
- Erbrechen bei Verwendung zur Gewichtsregulierung

Häufige Nebenwirkungen sind:

- Unterzuckerungen (Hypoglykämien), wenn Mounjaro® zusammen mit Insulin, Metformin und SGLT2-Hemmer angewendet wird.

Symptome einer Unterzuckerung können je nach Schweregrad von Schwitzen, Zittrigkeit und Unwohlsein bis hin zu Ohnmacht, Krampfanfällen, Hirnschäden oder Tod umfassen. Eine schwere Hypoglykämie kann erfordern, dass ein Teilnehmer Glucagon Intranasal (BAQSIMI®) oder eine Ampulleninjektion (GlucaGen® HypoKit®) erhält und/oder Notdienste in Anspruch nimmt.

- Allergische Reaktionen (Überempfindlichkeit) z.B. Hautausschlag, Juckreiz und Ekzem
- Schwindel wurde bei Verwendung zur Gewichtsregulierung beobachtet
- Niedriger Blutdruck wurde bei Verwendung zur Gewichtsregulierung beobachtet
- weniger Hungergefühle (verminderter Appetit)
- Bauchschmerzen
- Erbrechen
- Verdauungsstörung (Dyspepsie)
- Verstopfung
- Flatulenz
- Rülpsen (Aufstossen)
- Blähungen
- Sodbrennen
- Haarausfall wurde bei Verwendung zur Gewichtsregulierung beobachtet
- Müdigkeit
- Reaktionen an der Injektionsstelle (z.B. Ausschlag oder Rötung)

Gelegentliche Nebenwirkungen:

- Unterzuckerungen (Hypoglykämien), wenn Mounjaro® mit Metformin angewendet wird

Seltene, aber gefährliche Nebenwirkungen sind:

- Schwere allergische Reaktionen (anaphylaktische Reaktionen und Angioödeme) wurden selten berichtet. Wenden Sie Sich sofort an Ihren Arzt bzw. Ihre Ärztin, wenn Sie solche Symptome bemerken: Hautausschläge, Juckreiz und plötzliche Schwellung von Hals, Gesicht, Mund oder Rachen; Nesselsucht und Schwierigkeiten beim Atmen.

## Risiken und Belastungen durch Untersuchungen in der Studie

Wir machen für diese Studie verschiedene medizinische Untersuchungen (🡪 Kapitel 5.2). Diese Untersuchungen sind bewährte Verfahren. Trotzdem können sie Risiken und Belastungen haben, das heisst, sie können unangenehm sein oder unerwünschte Nebenwirkungen haben. Die geplanten studienbezogenen Interventionen sind mit ähnlichen Risiken verbunden, wie sie Menschen mit T1D in ihrem täglichen Leben ausgesetzt sind. In dieser Studie gibt es folgende Risiken und Belastungen:

- Blutentnahme: Es kann zu vorübergehenden Schmerzen durch den Nadelstich kommen, oder zu Blutergüssen, Blutungen oder Schwellungen an der Einstichstelle führen. Es können zudem in seltenen Fällen Schwindel, Infektionen, Ohnmacht auftreten.
- Fingerstick-Blutentnahme (Kapillarblutmessung): Es kann zu vorübergehenden Schmerzen durch den Lanzettenstich kommen, es können leichte Blutergüsse und zeitweilen kleine Narben auftreten und es besteht ein sehr geringes Risiko einer lokalen Infektion.
- CGM-Sensor (Gerät zur kontinuierlichen Glukosemessung): Es besteht das Risiko einer lokalen Hautinfektion, Blutungen, Blutergüssen und einer Infektion mit Schwellung, Rötung und Schmerzen an der Einstichstelle.
- Hyperglykämie (hoher Blutzucker): Aufgrund der häufigen Blutzuckerüberwachung wird keine schwere Hyperglykämie erwartet, bei Menschen mit T1D besteht dieses Risiko jedoch immer. Symptome sind Durst, Müdigkeit, oder erhöhten Zuckergehalt im Urin. In schweren Fällen von Hyperglykämie kann es zu einer diabetischen Ketoazidose (DKA) oder einem Koma kommen, es kann zu Nierenversagen, Herzrhythmusstörungen, Herzinfarkt, Muskelabbau (Rhabdomyolyse) und sogar Tod führen.

# Finanzierung und Entschädigung

Diese Studie wird von der Universität Bern als Sponsor, vertreten durch Prof. Dr. José García-Tirado, initiiert und vollständig finanziert.

Die beteiligten Forschenden haben keinen unmittelbaren finanziellen Vorteil an der Durchführung dieser Studie.

Sie bekommen folgende Entschädigung, wenn Sie bei dieser Studie mitmachen:

- Für jede Studienvisite (V1 – V4): 50.- CHF pro Visite

Hinzu kommt für jede Studienvisite die Vergütung von Reisekosten, die durch die Teilnahme bedingt sind, bis zu einem Wert von 50.- CHF pro Studienbesuch (öffentlicher Transport 2. Klasse und / oder gefahrene Kilometer mit dem Auto und Parkplatzgebühren).

Durch die Teilnahme an der Studie entstehen keine zusätzlichen Kosten für Sie oder für Ihre Krankenkasse.

Die Ergebnisse dieser Studie können dazu beitragen, dass später das Studienmedikament möglicherweise eine Zulassung für die Behandlung von T1D erlangt. Daran sind Sie nicht beteiligt, wenn Sie bei dieser Studie mitmachen.

# Ergebnisse aus der Studie

Es gibt Ergebnisse, die Sie selbst betreffen. Diese Ergebnisse teilt Ihnen Ihr Prüfarzt mit. Es gibt auch Zufallsbefunde. Zufallsbefunde sind «Begleit-Ergebnisse», die nicht beabsichtigt sind. Das können z.B. abnormale Messwerte sein. Wir informieren Sie, wenn diese Zufallsergebnisse relevant sind für Ihre Gesundheit.

Wir informieren Sie zum Beispiel, wenn wir zufällig eine Erkrankung feststellen, von der Sie noch nichts wissen. Wir informieren Sie auch, wenn wir ein Risiko für eine Erkrankung finden, die man durch vorbeugende Massnahmen verhindern kann.

Es gibt auch die Gesamtergebnisse der Studie, die aus den Daten von allen Teilnehmenden kommen. Dazu gehört zum Beispiel, dass wir mehr wissen über die Sicherheit und Wirksamkeit von automatischen Insulinabgabesystemen (AID) in Kombination mit Tirzepatid bei Menschen mit T1D (🡪 Kapitel 4.1). Diese Ergebnisse betreffen Sie und Ihre Gesundheit nicht direkt. Ihr Prüfarzt gibt Ihnen nach dem Ende der Studie, sobald die Daten ausgewertet sind, gerne eine Zusammenfassung der Gesamtergebnisse der Studie, wenn Sie das wünschen.

Teil 3:
Datenschutz und Versicherungsschutz

# Schutz von Daten

Wir schützen Ihre Daten (z.B. Angaben wie Blutdruck und Puls aus Ihrer Krankengeschichte). Zum Schutz von Daten gibt es in der Schweiz strenge gesetzliche Regelungen.

Das schweizerische Datenschutzgesetz gibt Ihnen das Recht auf Auskunft, Berichtigung und Erhalt Ihrer Daten, die im Rahmen der Studie erhoben, verarbeitet und weitergeleitet werden. Diese Rechte können in Ausnahmefällen wegen anderer gesetzlicher oder regulatorischer Anforderungen nicht immer garantiert werden. Wenn Sie Fragen dazu haben, wenden Sie sich bitte an Ihren Prüfarzt.

## Verschlüsselung von Daten

Bei jeder Studie entstehen Daten aus den Untersuchungen (z.B. Blutwerte, EKG-Auswertungen, Schwangerschaftstests). Diese Daten werden dokumentiert. Das passiert entweder in Papierform oder elektronisch in grossen Tabellen, den sogenannten «Datenerhebungsbögen». Alle Daten werden verschlüsselt dokumentiert. «Verschlüsselt» heisst, dass persönliche Informationen, die Sie direkt identifizieren können, *getrennt* von den Untersuchungsergebnissen aufbewahrt werden. Dazu gibt es eine Liste (Schlüsselliste), die jede Person mit einem eindeutigen Code identifiziert. So stehen z.B. Ihr Name, Ihr Geburtsdatum oder Ihr Wohnort *nicht* direkt im Datenerhebungsbogen. Diese Schlüsselliste bleibt für die Dauer von 10 Jahren nach der letzten Studienvisite aller Teilnehmer im Spitalarchiv Ihres Prüfarztes und wird anschliessend vernichtet. Niemand sonst bekommt diese Schlüsselliste. Spezielle Ausnahmen sind in Kapitel 9.5 geregelt.

Am Ende der vorgegebenen Aufbewahrungsdauer der Studienunterlagen werden Ihre Daten de-identifiziert. Das bedeutet, dass es nicht mehr möglich sein wird, Sie ohne unverhältnismässigen Aufwand zu identifizieren. Zur De-Identifikation werden verschiedene Massnahmen eingesetzt, u.a. die Vernichtung des Codes und der Schlüsselliste.

Wenn wir Daten zum Zwecke dieser Studie weitergeben – an den Sponsor oder an andere Fachpersonen oder Organisationen, die weitere Untersuchungen machen – dann sind die Daten immer verschlüsselt und Ihre persönlichen Daten sind geschützt. Das gilt auch, sollten die Daten ins Ausland weitergegeben werden.

## Sicherer Umgang mit den Daten während der Studie

Der Sponsor Universität Bern ist verantwortlich für den sicheren Umgang mit Ihren Daten aus dieser Studie. Er ist verantwortlich dafür, dass die geltenden Gesetze, z.B. die Datenschutzgesetze, eingehalten werden. Dies gilt auch, wenn (verschlüsselte) Daten für Untersuchungen in Länder verschickt werden, wo die Datenschutzgesetze weniger gut sind. So schützt der Sponsor dieser Studie Ihre Daten:

In dieser Studie werden Ihre Daten elektronisch erfasst und übermittelt. Die Daten sind auf einem Server in der Schweiz gespeichert. Trotzdem gibt es immer ein gewisses Restrisiko, dass fremde Personen auf Ihre verschlüsselten Daten zugreifen (z.B. Risiko von „Hacking“).

Oftmals ist es wichtig, dass Ihre Hausärztin / Ihr Hausarzt Daten Ihrer Krankengeschichte mit dem Prüfarzt teilt. Das gilt auch für andere Ärztinnen / Ärzte, die Sie behandeln. Mit der Einwilligung am Schluss des Dokuments erlauben Sie das.

## Sicherer Umgang mit Daten nach Ende der Studie

Der Sponsor bleibt auch nach Ende der Studie verantwortlich für den sicheren Umgang mit Ihren Daten. Das Gesetz schreibt vor, dass alle Studiendokumente, z.B. die Datenerhebungsbögen, für mindestens 10 Jahre aufbewahrt werden.

Nach Ende dieser langen Zeit bleiben Studiendaten verschlüsselt, dazu wird nach Ablauf der 10 Jahre die Schlüsselliste mit den Codes zu Ihren Bezugsdaten vernichtet werden. Gesundheitsrelevante Daten Ihrer Krankengeschichte, auch von dieser Studie, sind und bleiben für Ihre behandelnden Ärzte immer zugänglich.

Nach Abschluss einer Studie werden die Ergebnisse meist in wissenschaftlichen Zeitschriften veröffentlicht. Dazu werden die Ergebnisse durch andere Fachpersonen begutachtet. Ihre verschlüsselten Daten müssen dabei an diese Fachpersonen weitergeleitet werden. Es ist möglich, dass Ihre Daten in verschlüsselter Form im Rahmen dieser Studie zur statistischen Analyse an die Universität von Antioquia in Medellín, Kolumbien übermittelt werden. Der Sponsor trägt die Verantwortung dafür zu sorgen, dass im Ausland die gleichen Standards wie in der Schweiz eingehalten werden. Die Daten dürfen allerdings nicht für neue Forschungszwecke weiterverwendet werden. Dafür würde es Ihre separate Einwilligung brauchen (🡪 Kapitel 9.4).

## Weiterverwendung und Weitergabe Ihrer Daten in anderen, zukünftigen Studien

Ihre Daten aus dieser Studie sind für die zukünftige Forschung sehr wichtig. Die Daten können möglicherweise für andere Versuche weiterverwendet und/oder weitergegeben werden (auch ins Ausland).

Für die Weiterverwendung und/oder Weitergabe Ihrer Daten brauchen wir Ihre separate Einwilligung. Diese ist freiwillig. Bitte lesen Sie die zusätzliche Einwilligungserklärung am Schluss des Dokuments genau durch. Unterschreiben Sie bitte die Einwilligung, wenn Sie mit Ihren Daten weitere Forschung in der Zukunft unterstützen möchten. Wenn Sie nicht zustimmen, können Sie trotzdem an der Studie teilnehmen.

## Einsichtsrechte bei Kontrollen

Die Durchführung dieser Studie kann überprüft werden. Die Überprüfung geschieht durch Behörden wie die zuständige Ethikkommission oder die Zulassungsbehörde *Swissmedic*
oder auch durch ausländische Zulassungsbehörden. Auch der Sponsor muss solche Überprüfungen machen, damit die Qualität dieser Studie und die Ergebnisse gesichert sind.

Dafür erhalten wenige, speziell dafür ausgebildete Personen Einblick in Ihre persönlichen Daten und in Ihre Krankengeschichte. Für diese Überprüfung sind die Daten also *nicht* verschlüsselt. Die Personen, die Ihre unverschlüsselten Daten sehen, unterliegen der Schweigepflicht.

Als Studienteilnehmer haben Sie jederzeit das Recht, Ihre Daten einzusehen.

# Versicherungsschutz

Sie sind versichert, wenn Sie durch die Studie – also durch das Studienmedikament, oder das Studienprozedere – einen Schaden erleiden. Das Vorgehen ist gesetzlich geregelt. Dafür hat der Sponsor eine Versicherung bei Chubb Versicherungen (Schweiz) AG, Bärengasse 32, 8001 Zürich abgeschlossen. Wenn Sie meinen, dass Sie einen Schaden durch die Studie erlitten haben, wenden Sie sich bitte an Ihren Prüfarzt oder direkt an die Versicherung.

Bei Schäden, die auf ein zugelassenes und nach medizinischem Standard angewendetes Arzneimittel / Medizinprodukt zurückzuführen sind oder auch bei Anwendung einer üblichen Therapie aufgetreten wären, gelten dieselben Haftungsregelungen wie bei einer Behandlung ausserhalb einer Studie. In einem solchen Fall übernimmt die Haftpflichtversicherung des Spitals die Kosten / Entschädigung.

Teil 4:
Einwilligungserklärungen

Diese Einwilligung besteht aus zwei unabhängigen Einwilligungserklärungen:

- Einwilligungserklärung zur Teilnahme an dieser Studie *AID-JUNCT Studie*
- Einwilligungserklärung für die Weiterverwendung und Weitergabe von Daten aus dieser Studie in verschlüsselter Form für weitere Forschung.

Bitte lesen Sie dieses Formular sorgfältig durch. Bitte fragen Sie uns, wenn Sie etwas nicht verstehen oder wenn Sie noch etwas wissen möchten. Für die Teilnahme ist Ihre schriftliche Einwilligung notwendig.

### Einwilligungserklärung zur Teilnahme an der Studie *AID-JUNCT*

| **BASEC-Nummer** | 2024-01947 |
| --- | --- |
| **Titel der Studie** | GIP/GLP-1RA als Ergänzung zur automatischen Insulinabgabe bei Erwachsenen mit Typ-1-Diabetes: Eine Prospektive, randomisierte, klinische Studie |
| **Laien-verständlicher Titel** | Verwendung zusätzlicher Hormontherapie zur Ergänzung der automatischen Insulinabgabe bei Erwachsenen mit Typ-1-Diabetes: Eine auf noch nicht bereits erhobenen Daten basierende, randomisierte klinische Studie |
| **Verantwortliche Institution** (Sponsor mit Adresse) | Universität Bern  Hochschulstrasse 6  CH-3012 Bern |
| **Ort der Durchführung** | Kantonsspital Olten  Baslerstrasse 150  CH-4600 Olten |
| **Prüfarzt am Studienort** | PD Dr. med. Thomas Züger |
| **Teilnehmerin/ Teilnehmer:** Name und Vorname in Druckbuchstaben: Geburtsdatum: |  |

- Ich habe mündlich und schriftlich Informationen über die Studie bekommen, und zwar von dem Prüfarzt der unten unterschreibt.
- Der Prüfarzt hat mir den Zweck, den Ablauf und die Risiken des Studienprozederes und des Studienmedikaments erklärt.
- Ich nehme freiwillig an der Studie teil.
- Der Prüfarzt hat mir erklärt, welche möglichen Standardbehandlungen es ausserhalb der Studie gibt.
- Ich hatte genügend Zeit, um diese Entscheidung zu treffen. Ich behalte die schriftliche Information und erhalte eine Kopie meiner schriftlichen Einwilligungserklärung.
- Ich kann jederzeit meine Teilnahme beenden. Ich muss nicht erklären, warum. Auch wenn ich die Teilnahme beende, bekomme ich weiter meine medizinische Behandlung. Die Daten, die bis dahin gesammelt wurden, werden im Rahmen der Studie noch ausgewertet.
- Wenn ich zurücktrete, bleiben die Daten verschlüsselt.
- Wenn es besser für meine Gesundheit ist, kann mich der Prüfarzt jederzeit von der Studie ausschliessen.
- Bei Ergebnissen und Zufallsbefunden, die direkt meine Gesundheit betreffen, werde ich informiert.
- Mein Hausarzt / meine Hausärztin darf Daten meiner Krankengeschichte, die für die Studie wichtig sind, mit dem Prüfarzt teilen. Das gilt auch für andere Ärztinnen / Ärzte, die mich behandeln.
- Die zuständigen Fachpersonen des Sponsors, der Ethikkommission und der Arzneimittelbehörde *Swissmedic* dürfen meine unverschlüsselten Daten zur Kontrolle einsehen. Alle diese Personen unterstehen der Schweigepflicht.
- Ich weiss, dass der Sponsor, die Universität Bern, eine Versicherung abgeschlossen hat. Diese Versicherung bezahlt, wenn ich einen Schaden erleide – aber nur, wenn der Schaden direkt mit der Studie zusammenhängt.
- Ich bin damit einverstanden, dass meine verschlüsselten Daten im Rahmen dieser Studie möglicherweise zur statistischen Analyse an die Universität von Antioquia in Medellín, Kolumbien übermittelt werden.

| Ort, Datum | Name und Vorname Teilnehmerin / Teilnehmer  in Druckbuchstaben  Unterschrift Teilnehmerin / Teilnehmer |
| --- | --- |

**Bestätigung des Prüfarztes:** Hiermit bestätige ich, dass ich dieser Teilnehmerin / diesem Teilnehmer Art, Bedeutung und Tragweite der Studie erläutert habe. Ich versichere, alle mit dieser Studie in Zusammenhang stehenden Verpflichtungen nach Schweizer Recht zu erfüllen. Sollte ich im Verlauf der Studie von Aspekten erfahren, welche die Bereitschaft der Teilnehmerin / des Teilnehmers zur Studienteilnahme beeinflussen könnten, werde ich sie / ihn umgehend darüber informieren.

| Ort, Datum | Name und Vorname des Prüfarztes in Druckbuchstaben  Unterschrift des Prüfarztes |
| --- | --- |

### Einwilligungserklärung für die Weiterverwendung und/oder Weitergabe von Daten in verschlüsselter Form

Diese Einwilligung betrifft Sie nicht im Sinne der persönlichen Teilnahme an einer Studie. (🡪 Kapitel 9.4 der Patienteninformationen).

«Weiterverwendung» meint, dass Ihre Daten über die Zeit Ihrer Studienteilnahme hinaus aufbewahrt und in verschlüsselter Form für weitere Forschung verwendet werden können. Das kann z.B. heissen, dass entsprechende Laborwerte von Ihnen zusammen mit einer grossen Zahl von anderen Werten statistisch ausgewertet werden oder neue Untersuchungen damit durchgeführt werden.

«Weitergabe» meint, dass Ihre Daten an andere Forschungspersonen oder Forschungsinstitutionen in verschlüsselter Form für weitere Forschungsprojekte weitergegeben werden dürfen. Diese anderen Forschungspersonen oder Forschungsinstitutionen können auch im Ausland angesiedelt sein. Es ist die Verantwortung des Sponsors, dass dieses Land über ein angemessenes Datenschutzniveau verfügt, welches mit der Schweiz vergleichbar ist.

| **BASEC-Nummer:** | 2024-01947 |
| --- | --- |
| **Titel der Studie** | GIP/GLP-1RA als Ergänzung zur automatischen Insulinabgabe bei Erwachsenen mit Typ-1-Diabetes: Eine Prospektive, randomisierte, klinische Studie |
| **Laien-verständlicher Titel** | Verwendung zusätzlicher Hormontherapie zur Ergänzung der automatischen Insulinabgabe bei Erwachsenen mit Typ-1-Diabetes: Eine auf noch nicht bereits erhobenen Daten basierende, randomisierte klinische Studie |
| **Teilnehmerin/Teilnehmer:**   - - Name und Vorname in Druckbuchstaben:   - Geburtsdatum: |  |

- Ich erlaube, dass meine verschlüsselten Daten aus dieser Studie für die medizinische Forschung weiterverwendet und weitergegeben (auch ins Ausland) werden dürfen. Sie stehen dann für zukünftige, weitere Forschungsprojekte auf unbestimmte Zeit zur Verfügung.
- Ich habe verstanden, dass die Daten verschlüsselt sind und der Schlüssel sicher aufbewahrt wird.
- Die Daten können im In- und Ausland ausgewertet werden und in einer Datenbank hier oder im Ausland gespeichert werden. Forschungsinstitutionen im Ausland müssen dieselben Standards zum Datenschutz einhalten, wie sie in der Schweiz gelten.
- Ich entscheide mich freiwillig für die Weiterverwendung und/oder Weitergabe von Daten in verschlüsselter Form und kann diesen Entscheid zu jedem Zeitpunkt zurücknehmen. Ich informiere lediglich meinen Prüfarzt und muss diesen Entscheid nicht begründen.
- Wenn ich zurücktrete, bleiben die Daten verschlüsselt.
- Normalerweise werden alle Daten zusammengefasst ausgewertet. Wenn sich zufällig ein Ergebnis zeigt, das für meine Gesundheit sehr wichtig ist, werde ich kontaktiert.

| Ort, Datum | Name und Vorname Teilnehmerin / Teilnehmer  in Druckbuchstaben  Unterschrift Teilnehmerin / Teilnehmer |
| --- | --- |

**Bestätigung des Prüfarztes:** Ich bestätige, dass ich der Teilnehmerin/dem Teilnehmer Art, Bedeutung und Tragweite der Weiterverwendung und/oder Weitergabe von Daten erläutert habe.

| Ort, Datum | Name und Vorname des Prüfarztes in Druckbuchstaben  Unterschrift des Prüfarztes |
| --- | --- |

1. Im Gesetz wird dafür der Begriff „klinischer Versuch“ verwendet. [↑](#footnote-ref-2)
