# Supplementary material for: GIP/GLP-1RA as adjunctive to automated insulin delivery in adults with Type 1 diabetes (the AID-JUNCT trial): Study protocol for a prospective, randomized, clinical trial
Source: PLoS One. 2025 Oct 22;20(10):e0335060. doi: 10.1371/journal.pone.0335060 (PMC12543135; doi:10.1371/journal.pone.0335060)
Supplement: S1 Text — (PDF) [file pone.0335060.s003.pdf]

Docusign Envelope ID: 11555059-DCA3-47FE-9701-55DBADEB3B9E

GIP/GLP-1RA as Adjunctive to Automated Insulin Delivery in Adults with Type 1 Diabetes: A Prospective, Randomized, Clinical Study – The AID-JUNCT Trial

Clinical Study Protocol

Study Type:	Clinical trial with Investigational Medicinal Product (IMP)
Study Categorization:	Risk category according to ClinO (B)
Study Registration:	ClinicalTrials.gov ID NCT06630585<br>Swiss National Clinical Trial Portal (SNCTP) pending
Study Identifier:	AID-JUNCT Trial
Sponsor:	University of Bern<br>Hochschulstrasse 6<br>CH-3012 Bern<br>Contact: Prof. Dr. José F. García-Tirado<br>Tel: 078 267 52 12<br>E-mail: jose.garcia@unibe.ch
Principal Investigator:	PD Dr. med. Thomas Züger<br>Chefarzt – Kantonsspital Olten<br>Baslerstrasse 150<br>CH-4600 Olten<br>Tel: 062 311 44 94<br>E-mail: thomas.zueger@spital.so.ch
Investigational Product:	*Mounjaro*® [tirzepatide]
Protocol Version and Date:	1.1 (dated 04.11.2024)

CONFIDENTIAL

The information contained in this document is confidential and the property of the Sponsor. The information may not - in full or in part - be transmitted, reproduced, published, or disclosed to others than the applicable Competent Ethics Committee(s) and Regulatory Authority(ies) without prior written authorization from the sponsor except to the extent necessary to obtain informed consent from those who will participate in the study.

SIGNATURE PAGES DELETED FROM THIS COPY

Table of Contents

SIGNATURE PAGE(S) **2**
STUDY SYNOPSIS **7**
ABBREVIATIONS **10**
SUMMARY OF THE REVISION HISTORY IN CASE OF AMENDMENTS **12**
STUDY SCHEDULE **12**
1. STUDY ADMINISTRATIVE STRUCTURE **15**
Sponsor, Sponsor-Investigator 15
Principal Investigator(s) 15
Statistician ("Biostatistician") 15
Laboratory 15
Monitoring institution 15
Data Safety Monitoring Committee 15
2. ETHICAL AND REGULATORY ASPECTS **16**
2.1 Study registration 16
2.2 Categorization of Study 16
2.3 Competent Ethics Committee (CEC) 16
2.4 Competent Authorities (CA) 16
2.5 Ethical Conduct of the Study 16
2.6 Declaration of Interest 17
2.7 Patient Information and Informed Consent 17
2.8 Participant privacy and confidentiality 17
2.9 Early termination of the study 17
2.10 Protocol amendments 17
2.11 Deviation from the CIP 18
3. BACKGROUND AND RATIONALE **19**
3.1 Background and Rationale 19
3.2 Investigational Product (treatment) and Indication 20
3.3 Preclinical Evidence 20
3.4 Clinical Evidence to Date 21
3.5 Rationale for the dosage, route, and regimen 22
3.6 Explanation for choice of comparator (or placebo) 22
3.7 Risks / Benefits 22
3.8 Justification of choice of the study population 25
4. STUDY OBJECTIVES **26**
4.1 Overall Objective 26
4.2 Primary Objective 26
4.3 Secondary Objectives 26
4.4 Safety Objectives 26
5. STUDY OUTCOMES **27**
Primary Outcome 27
Secondary Outcomes 27
Exploratory Outcomes 27
Other Outcomes of Interest 28
Safety Outcomes 28

6. STUDY DESIGN 29
6.1 General study design and justification of design 29
6.2 Methods of Minimizing Bias 29
6.2.1 Randomization 29
6.2.2 Blinding procedures 29
6.2.3 Other methods of minimizing bias 29
6.3 Unblinding Procedures (Code break) 29
7. STUDY POPULATION 30
7.1 Eligibility criteria 30
7.1.1 Inclusion criteria 30
7.2 Recruitment and screening 31
7.3 Assignment to Study Groups 31
7.4 Criteria for withdrawal/discontinuation of participants 32
8. STUDY INTERVENTION 33
8.1 Identity of Investigational Products 33
8.1.1 Experimental Intervention 33
8.1.2 Control Intervention (standard/routine/comparator treatment) 33
8.1.3 Packaging, Labelling, and Supply (re-supply) 33
8.1.4 Storage Conditions 33
8.2 Administration of experimental and control interventions 33
8.2.1 Experimental Intervention 33
8.2.2 Control Intervention 33
8.3 Dose modifications 34
8.4 Compliance with Study Intervention 34
8.5 Data Collection and Follow-up for Withdrawn Participants 34
8.6 Trial-specific Preventive Measures 34
8.7 Concomitant Interventions (treatments) 34
8.8 Study Drug Accountability 35
8.9 Return or Destruction of Study Drug 35
8.10 Study accessories 35
9.1 Study flow chart(s) / table of study procedures and assessments. 37
9.2 Assessments of Outcomes 37
9.2.1 Assessment of primary outcome 37
9.2.2 Assessment of secondary (exploratory) outcomes 37
9.2.3 Assessment of other outcomes of interest 37
9.2.4 Assessment of safety outcomes 38
9.2.5 Assessments in participants who prematurely stop the study. 38
9.3 Procedures at each visit 39
9.3.1 Visit 1 – Screening. 39
9.3.2 Visit 2 – Randomization. 40
Additionally, all participants will receive the CGM Dexcom G7 system (re-supply), a Ketone meter, and additional supplies (test strips, lancing devices, and lancets). 40
9.3.3 Phone Visit 1 40
9.3.4 Visit 3 40
9.3.7 Phone Visit 4 – Follow-up 41

10. SAFETY ... 42
10.1 Drug studies ... 42
10.1.1 Definition and Assessment of (Serious) Adverse Events and Other Safety-Related Events... 42
10.1.2 Reporting of serious adverse events (SAE) and other Safety-Related events ... 43
10.1.2.2 Reporting of SAEs ... 45
10.1.2.3 Reporting of SUSARs ... 45
10.1.2.4 Reporting of immediate safety and protective measures ... 45
10.1.2.5. Reporting and Handling of Pregnancies ... 46
10.1.2.6 Periodic reporting of safety ... 46
10.1.3 Follow-up of (Serious) Adverse Events ... 46
10.2 Assessment, notification, and reporting on the use of radiation sources ... 46
11. STATISTICAL METHODS ... 47
11.1 Hypothesis ... 47
11.2 Determination of Sample Size ... 47
11.3 Statistical criteria for termination of trial ... 47
11.4 Planned Analyses ... 47
11.4.1 Datasets to be analyzed, analysis populations ... 47
11.4.2 Primary Analysis ... 47
11.4.3 Secondary Analyses ... 47
11.4.4 Interim analyses ... 48
11.4.5 Safety analysis ... 48
11.4.6 Deviation(s) from the original statistical plan ... 48
11.5 Handling of missing data and drop-outs ... 48
12. QUALITY ASSURANCE AND CONTROL ... 49
12.1 Data handling and record keeping/archiving ... 49
12.1.1 Case Report Forms ... 49
12.1.2 Specification of source documents ... 49
12.1.3 Record keeping/archiving ... 49
12.2 Data management ... 50
Data Management System ... 50
12.2.1 Data security, access, and back-up ... 50
12.2.2 Analysis and archiving ... 50
12.2.3 Electronic and central data validation ... 50
12.3 Monitoring ... 50
12.4 Audits and Inspections ... 50
12.5 Confidentiality, Data Protection ... 50
12.6 Storage of biological material and related health data ... 51
13. PUBLICATION AND DISSEMINATION POLICY ... 52
14. FUNDING AND SUPPORT ... 53
14.1 Funding ... 53
14.2 Other Support ... 53
15. INSURANCE ... 54
16. REFERENCES ... 55

STUDY SYNOPSIS

**Sponsor**	University of Bern
**Study Title:**	GIP/GLP-1RA as Adjunctive to Automated Insulin Delivery in Adults with Type 1 Diabetes: A Prospective, Randomized, Clinical Study – The AID-JUNCT Trial.
**Short Title / Study ID:**	The AID-JUNCT Trial
**Protocol Version and Date:**	1.1 (04.11.2024)
**Trial registration:**	ClinicalTrials.gov ID NCT06630585
**Study category and Rationale**	Category B: This investigational medicinal product (IMP) is authorized in Switzerland to treat adults with type 2 diabetes mellitus (T2D) or obesity, in addition to diet and exercise. There is no current indication from the local authorities to use the IMP for type 1 diabetes (T1D) mellitus.
**Clinical Phase:**	Clinical study phase 3
**Background and Rationale:**	Glycemic control in type 1 diabetes (T1D) remains a challenge, with ~32% of adults in Switzerland achieving an A1c target of <7%. The dual glucagon-like peptide-1 receptor agonists (GLP-1RAs) and glucose-dependent insulinotropic polypeptide (GIP)/GLP-1RAs has emerged as promising therapy in T1D. Retrospective studies have shown people with T1D can significantly improve glycemic control with a reduction in insulin dose and body weight when long-acting GLP-1RAs or GIP/GLP-1RAs are added to insulin therapy. However, randomized controlled trials (RCT) are still lacking.
**Objective(s):**	**Primary Objective**<br>Evaluate the efficacy of tirzepatide (*Mounjaro*®) 5mg as adjunctive therapy to automatic insulin delivery (AID) system on continuous glucose monitor (CGM)-based percent time in the target range (TIR) in adults with T1D.<br>**Secondary Objectives**<br>Evaluate the efficacy of tirzepatide (Mounjaro®) 5 mg as adjunctive therapy to automated insulin delivery (AID) on CGM-based glucose metrics, HbA1c, and insulin delivery in adults with type 1 diabetes (T1D).<br>Evaluate the efficacy of tirzepatide (Mounjaro®) 5 mg as adjunctive therapy to AID on anthropometric measures and body composition in adults with T1D.<br>Evaluate the efficacy of tirzepatide (Mounjaro®) 5 mg as adjunctive therapy to AID on cardio-renal outcomes in adults with T1D.<br>Evaluate the safety of tirzepatide (Mounjaro®) 5 mg as adjunctive therapy to AID in adults with T1D.

<table>
<tr><td>Outcome(s):</td><td>
Primary Outcome
Mean difference in 24/7 CGM percentage time spent between 3.9 and 10 mmol/L (TIR) between groups at week 16 of treatment.
Secondary Outcomes
Secondary endpoints will be tested hierarchically to maintain the type I error at 5% in the following order
Mean difference between groups at week 16 of treatment in
1. Glycated Hemoglobin (HbA1C)
2. CGM-measured TIR between 0700 and 2300 ($TIR_{7\text{-}23}$)
3. CGM-measured time in the tight range (TTR, 3.9 – 7.8 mmol/L)
4. 24/7 Total Daily Insulin (TDI)
5. 24/7 CGM-measured percent time >10.0 mmol/L
6. 24/7 CGM-measured percent time <3.9 mmol/L
7. 24/7 CGM-measured percent time >13.9 mmol/L
8. 24/7 CGM-measured percent time <3.0 mmol/L
Exploratory Outcomes
Mean difference between groups at weeks 8, 12, and 16 of treatment in
CGM-measured TIR between 0700 and 2300 ($TIR_{7\text{-}23}$)
24/7 CGM-measured percent time >10 mmol/L
24/7 CGM-measured percent time >13.9 mmol/L
24/7 CGM-measured percent time <3.9 mmol/L
24/7 CGM-measured percent time <3.0 mmol/L
CGM-measured glucose variability measured with the coefficient of variation (CV)
24/7 Total Daily Insulin (TDI)
24/7 Total Daily Bolus (TDBo)
Mean difference between groups at week 16 of treatment in
Anthropometric-Body Composition:
- Body weight
- Waist circumference
- Hip circumference
- Waist-to-hip ratio
- % fat mass
- Fat mass index (FMI)
- Visceral adipose tissue (VAT)
- Lean mass index (LMI)
- Appendicular lean mass index (ALMI)
Cardio-Renal:
- Low-density cholesterol lipoprotein (LDL)
- High-density cholesterol lipoprotein (HDL)
- Cholesterol non-HDL
- Triglycerides (TG)
- TG-glucose index in fasting.
- Systolic and diastolic blood pressure
- Urine albumin to creatinine ratio (uACR)
- Hepatic steatosis and fibrosis assessed by FibroScan (CAP-value and LSM)
- MASLD biomarkers, hepatic steatosis index (HIS), and fibrosis score (FIB-4).
- MASLD biomarkers, hepatic steatosis index (HIS), and fibrosis score (FIB-4).
Other Outcomes of Interest
- Describe reported eating behavior and food intake (using the SNAQ App).
- Describe patient-reported outcomes: DTSQc (baseline)/DTSQc (last visit-end of treatment)
</td></tr>
<tr><td>Study design:</td><td>Parallel, open-label; randomized, clinical trial</td></tr>
</table>

**Inclusion / Exclusion criteria:**	**Inclusion criteria**:<br>Participants with diagnosed T1D for at least 12 months, aged between 18 to 65 years old (inclusive), currently on AID therapy for at least three months, with A1C ≥6.5% and ≤10% and BMI ≥23 kg/m^2, will be included in the study.<br>**Key exclusion criteria:**<br>Use of SGLT2i, GLP-1 receptor agonist (exenatide, liraglutide, or other), dual GIP/GLP-1RA, pramlintide or weight loss medications within 90 days before screening, use of any dipeptidyl peptidase 4 (DPP-IV) inhibitors within 30 days before screening, history of proliferative retinopathy, diabetes ketoacidosis (DKA) within six months before screening, chronic pancreatitis or idiopathic acute pancreatitis, serum triglycerides ≥5.7 mmol/L (500mg/dL), personal or family history of multiple endocrine neoplasia type 2 (MEN2) or familial medullary thyroid carcinoma - Screening calcitonin above or equal to 35 ng/L, people with diabetic gastropathy, renal chronic disease (eGFR <30 mL/min/1.73 m^2 by CKD-EPI), impaired liver function, history of severe hypoglycemic event (defined as seizure or loss of consciousness) in the past six months will be excluded.
**Measurements and procedures:**	The study consists of a screening, and eligibility assessment, followed by two weeks of baseline (run-in) period, randomization, four weeks of IMP up-titration, and 16 weeks of assessment as follows:<br>Visit 1 – Screening<br>Visit 2 – Randomization<br>Phone visit 1 – Escalation to target dose (intervention arm) and follow-up (control arm)<br>Visits 3 – Outcomes evaluation<br>Phone visit 2 - Outcomes evaluation<br>Visit 4 - Outcomes evaluation (Last visit)<br>Phone Visit 4 – Follow-up (intervention arm)
**Study Product / Intervention:**	Tirzepatide (*Mounjaro*®) is a clear, colorless to slightly yellow solution available in cartons containing four pre-filled single-dose pens or one single-dose vial: 2.5 mg, 5 mg, 7.5 mg, 10 mg, 12.5 mg, or 15 mg per 0.5 mL in a single-dose pen or vial.<br>In this study, we will start with the recommended starting dosage of tirzepatide (Mounjaro®): 2.5 mg injected subcutaneously once weekly (for four weeks). After four weeks, the dosage will be increased to 5 mg once weekly injected subcutaneously for 16 weeks.
**Control Intervention (if applicable):**	The participants in the control intervention will be asked to continue using their AID+ standard of care (SoC).
**Number of Participants with Rationale:**	The number of participants for the study is 42 (21 participants in each arm). Sample size estimation based on an analysis of covariance (ANCOVA) of the change in the Continuous Glucose Monitor (CGM)-based percentage time in the target range (TIR) from baseline to the end of a three-month period.
**Study Duration:**	The estimated duration of the main investigational plan is one year, from the screening until the last follow-up visit.
**Study Schedule:**	First-Participant in Q4-2024 (planned)<br>Last-Participant-Out in Q1-2026 (planned)
**Investigator(s):**	PD Dr. med. Thomas Züger<br>Chefarzt – Kantonsspital Olten<br>Baslerstrasse 150<br>CH-4600 Olten<br>Tel: 062 311 44 94<br>E-mail: thomas.zueger@spital.so.ch
**Study Centre(s):**	Solothurner Spitäler AG<br>c/o Kantonsspital Olten<br>Baslerstrasse 150<br>4600 Olten<br>062 311 48 19
**Statistical Considerations:**	For the main outcome, we estimated a sample size of 42 participants (21 in each arm), assuming 80% power and a two-sided type I error rate of 5% to reject the null hypothesis. The following assumptions were made: a baseline standard deviation (SD) for TIR of 15%, a pre-post difference in TIR of 0.2% in the control group, a pre-post difference of 10.5% in the treatment group, a correlation of 0.7, and a dropout rate of 20%.
**GCP Statement:**	This study will be conducted in compliance with the protocol, the current version of the Declaration of Helsinki, ICH-GCP, and all national legal and regulatory requirements.

ABBREVIATIONS

AE	Adverse Event
ASR	Annual Safety Report
AID	Automatic Insulin Delivery
BASEC	Business Administration System for Ethical Committees, (https://submissions.swissethics.ch/en/)
BIA	Bioelectrical Impedance Analysis
BMI	Body Mass Index
BP	Blood Pressure
CA	Competent Authority (e.g. Swissmedic)
CAP	Controlled Attenuation Parameter
CEC	Competent Ethics Committee
CRF	Case Report Form
CRA	Clinical Research Associate
ClinO	Ordinance on Clinical Trials in Human Research *(in German: KlinV, in French: OClin, in Italian: OSRUm)*
CTCAE	Common terminology criteria for adverse events
CGM	Continuous Glucose Monitoring
CVD	Cardiovascular Disease
DCB	Diabetes Center of Bern
DIO	Diet-induced obese
DKA	Diabetic ketoacidosis
DTSQ	Diabetes Treatment Satisfaction Questionnaire
ECG	Electrocardiogram
eCRF	Electronic Case Report Form
eGFR	Estimated Glomerular Filtration Rate
GCP	Good Clinical Practice
GIP	glucose-dependent insulinotropic polypeptide
GIPR	GIP receptor
GLP	Glucagon-like Peptide
GMP	Good Manufacturing Practice
Ha	Alternative Hypothesis
Ho	Null hypothesis
HR	Heart Rate
HRA	Federal Act on Research involving Human Beings *(in German: HFG, in French: LRH, in Italian: LRUm)*
IB	Investigator's Brochure
IMP	Investigational Medicinal Product
IIT	Investigator-initiated Trial

ISF	Investigator's Site File
ITT	Intention to treat
LSM	Liver Stiffness Measurement
MASH	Metabolic dysfunction-associated steatohepatitis
MASLD	Metabolic Dysfunction-Associated Steatotic Liver Disease
PI	Principal Investigator
PP	Per Protocol
SC	Subcutaneous
SDV	Source Data Verification
SoC	Standard of Care
SOP	Standard Operating Procedure
SPC	Summary of product characteristics
SUSAR	Suspected Unexpected Serious Adverse Reaction
TDI	Total Daily Insulin
TMF	Trial Master File
T1D	Type 1 diabetes
T2D	Type 2 diabetes
UPN	Unique Patient Number

Docusign Envelope ID: 11555059-DCA3-47FE-9701-55DBADEB3B9E

SUMMARY OF THE REVISION HISTORY IN CASE OF AMENDMENTS

Version Nr, Version Date	Chapter	Description of change	Reason for the change
1.0 (date: 18.09.2024)	Not applicable	Initial document	Not applicable
1.1 (date 04.11.2024)	Not applicable	Harmonization, AE/SAE reporting, and outcomes (updated)	Swissmedic and BASEC ethics requests

STUDY SCHEDULE

Table 1. Visit Schedule.

	Screening	Randomization	Treatment Period				Follow-up
Visit (V) or Phone (P)	V1	V2	P1	V3	P2	V4	P3[a]
Weeks	-2	0	4	8	12	16	18
Window (days)	-7	14 up to 21 days after V1	±3	±3	±3	±3	±7
Subject-related information/ assessments							
Informed consent	X						
Inclusion/Exclusion	X						
Randomization		X					
Screen fail/withdrawal criteria	X	X	X	X	X	X	
Medical history/demography[b]	X						
Concomitant medications	X						
Trial-related outcomes measures and downloads							
Weight, Height, Waist & Hip circumference	X			X		X	
Vital signs (BP, HR, Temperature[c])	X	X		X		X	
ECG	X			X		X	
Body composition (BIA)		X				X	
Hepatic elastography (*FibroScan®*)		X				X	
Device downloads (CGM and pump data)		X	X	X	X	X	
SNAQ app data downloads		X		X		X	
Activity tracker downloads		X		X		X	
Questionnaires (DTSQs-c)		X				X	
AE/SAE assessment including assessment for clinical significance and severe	X	X	X	X	X	X	X

hypoglycemia and DKA							
Laboratory							
HbA1c (central)	X					X	
Hematology (hb-hct)	X						
Serum/urine pregnancy test (for child-bearing potential women)[d]	X	X		X		X	
Hepatic chemistry/coagulation	X[e]			X[f]		X[f]	
Endocrine	X[g]					X[h]	
Pancreas (exocrine) panel	X			X		X	
Lipid panel (fasting)	X					X	
Glucose fasting	X						
Urine chemistry	X[i]			X[j]		X[i]	
Supply, re-supply and instructions/training							
IMP dispensing/re-supply		X[a]		X			
Study phones[k] and activity trackers dispensing	X						
Ketone meter dispensing		X					
Dexcom G7 system dispensing/re-supply	X	X		X			
Supplies[l] dispensing/re-supply		X		X			
Medication logbook handover		X[a]		X[a]			
Hypoglycemia diary handover	X	X					
Study handbook	X						
IMP instructions/training		X[a]					
Ketone meter instructions/training		X					
Study apps and activity tracker installation/instructions/training	X						
Others							
IMP accountability/compliance check				X		X	
Medication logbook review and collection				X		X	
Hypoglycemia diary review		X	X	X	X	X	
Handout of documents/reminders		X	X	X	X	X	
Return non-used pens of the IMP						X	
De-installation study apps						X	
Return study devices (activity tracker, phones, and ketone meters)						X	

Abbreviations: BMI= body mass index; BG= blood glucose; BP = blood pressure; HR= heart rate; BIA= bioelectrical impedance analysis; CGM=continuous glucose monitoring; DTSQs-c= diabetes treatment satisfaction questionnaire status and change versions; ECG= electrocardiogram; U/S=unscheduled; P/D= premature discontinuation; DKA=diabetes ketoacidosis; HbA1c = hemoglobin A1c; Hb= hemoglobin; HCT= hematocrit; IMP=investigational medicinal product; FDA=Food and Drug

Administration.

[a]Only for the participants in the intervention arm.
[b]Included: gender, race/ethnicity, and year of birth.
[c]The temperature should be measured at the screening visit (V1) and randomization visit (V2) to exclude infection.
[d]A serum pregnancy test will be performed at the screening visit, the result must be available before randomization and the first injection of the study drug for women of childbearing potential only. Additional pregnancy in urine tests will be repeated during the study (**See annex 6-6.1**).
[e]Include: total bilirubin, direct bilirubin, alkaline phosphatase (ALP), partial thromboplastin time (PTT), prothrombin time (PT), international normalized ratio (INR), alanine aminotransferase (ALT), aspartate aminotransferase (AST), and gamma-glutamyl transferase (GGT). (**See annex 6-6.1**).
[f]Include: total bilirubin, direct bilirubin, ALT, AST. (**See annex 6-6.1**).
[g]Include: thyroid stimulating hormone (TSH), free thyroxine (FT4), calcitonin, C-peptide (fasting), estradiol, and follicle-stimulating hormone (FSH) (**See annex 6-6.1**).
[h]Only calcitonin (**See annex 6-6.1**).
[i]Include: creatinine (with eGFR by CKD-EPI), and urine microalbumin/creatinine ratio (uACR) (**See annex 6-6.1**).
[j]Only creatinine (with eGFR by CKD-EPI) (**See annex 6-6.1**).
[k]The participant also can use her/his phone if it is compatible with Dexcom G7 and the study apps.
[l]Include: test strips, lancing device, and lancets.

Docusign Envelope ID: 11555059-DCA3-47FE-9701-55DBADEB3B9E

1. STUDY ADMINISTRATIVE STRUCTURE

Sponsor, Sponsor-Investigator

The Sponsor is involved in the study design and data collection and interpretation.

University of Bern
Hochshulstrasse 6
CH-3012 Bern

Contact: José F. Garcia-Tirado, PhD
Assistant Professor, University of Bern.
Freiburgstrasse 3, Sitem/Insel Building, 4OG, Diabetes Center Berne
CH-3010 Bern
Tel: 078 267 52 12
E-mail: jose.garcia@unibe.ch

Principal Investigator(s)

PD Dr. med. Thomas Züger
Chefarzt – Kantonsspital Olten
Baslerstrasse 150
CH-4600 Olten
Tel: 062 311 44 94
E-mail: thomas.zueger@spital.so.ch

Statistician ("Biostatistician")

Jaime Alberto Gallo Villegas. MD. PhD
Full Professor, University of Antioquia
Medellín, Colombia
Tel: +57 3006183590
E-mail: jaime.gallo@udea.edu.co

Laboratory

Central Laboratory
Institut für Labormedizin
Solothurner Spitäler AG
c/o Kantonsspital Olten
Baslerstrasse 150
4600 Olten
Tel: 062 311 48 19

Monitoring institution

DCB Research AG
Freiburgstrasse 3
CH-3010 Bern

Data Safety Monitoring Committee

Not applicable.

Co-Investigator / Clinical Researcher / Clinical Project Manager

Dr. med. María Carolina Fragozo-Ramos
MD PhD student, University of Bern.
Freiburgstrasse 3, Sitem/Insel Building, 4OG, Diabetes Center Berne
CH-3010 Bern
Tel: 078 267 1298
E-mail: maria.fragozoramos@students.unibe.ch

2. ETHICAL AND REGULATORY ASPECTS

Type 1 diabetes (T1D) is a chronic disease with an increasing incidence and prevalence (1–3). Intensified insulin treatments have led to a significant reduction in the risk of development and progression of long-term microvascular complications in T1D (4,5). However, this intensification also leads to more hypoglycemia events and weight gain with an increase in cardiovascular risk factors (6,7).

This study will test the safety and efficacy of the dual glucose-dependent insulinotropic polypeptide/glucagon-like peptide-1 receptor agonist (GIP/GLP1-RA) (*Mounjaro®* [tirzepatide]) as an adjunct therapy in people with T1D with existing insulin therapy through automated insulin delivery (AID). The results of this study will make an important contribution to the care of patients with T1D by improving their self-management, reducing the T1D burden, and improving their quality of life. The trial may result in improved glucose control and weight loss for the participants, indicating the potential for this molecule to become part of standard care for T1D in the future. Participants will be provided with written informed consent and fully informed about the study, including the risk-benefit ratio (**See Section 3.1**). Their right to stop the procedures and withdraw the informed consent at any time during the study will be respected.

The sponsor will submit the protocol, the proposed patient information and consent form, and other study-specific documents to the "Ethikkommission Nordwest-und Zentralschweis" (EKNZ) and Swissmedic before the study commencement. The clinical study will only begin once approval from all required authorities has been received. Any additional requirements imposed by the authorities will be implemented.

2.1 Study registration

This study will be registered at www.clinicaltrials.gov and in the Swiss National Clinical Trial Portal (SNCTP) at www.kofam.ch.

2.2 Categorization of Study

This study examines the safety and feasibility of adding a medicinal product as adjunctive therapy to AID in T1D. The investigational medicinal product (IMP) is authorized in Switzerland for treating people with type 2 diabetes (T2D) but will not be used as intended in Paragraph 1 of Chapter 2, Article 19 of ClinO (8,9). Therefore, the study is classified under Category B according to the Swiss HRA and its corresponding Ordinance Clin-O on clinical trials.

2.3 Competent Ethics Committee (CEC)

The Sponsor will submit the investigation to the corresponding CEC to obtain Ethical approval before the investigation starts. The Sponsor and the principal investigator (PI) will also ensure that approval from the CEC is obtained and filed in the investigator site file (ISF) before starting the trial.

No protocol changes are made without the sponsor's and the CEC's prior approval, except when necessary to eliminate immediate hazards to study participants.

The premature end or interruption of the study will be reported within 15 days. The regular end of the study will be reported to the CEC within 90 days; the final study report will be submitted within one year after the study ends. Amendments are reported according to **Section 2.10**.

2.4 Competent Authorities (CA)

The Sponsor will submit the investigation to the CA for approval before the clinical trial starts. The Sponsor ensures that approval from the CA is obtained and filed in the investigator site file before the commencement of the study (**See Section 2.2**).

Premature study termination or interruption and the regular end of the study will be reported within the same timeline as for CEC (**See Section 2.3**), except for non-substantial amendments that will be reported as soon as possible. For Amendments, **see Section 2.10**.

2.5 Ethical Conduct of the Study

The study will be carried out following the protocol and with principles enunciated in the current version of the Declaration of Helsinki (10), the guidelines of Good Clinical Practice (GCP) issued by the International Conference on Harmonization (ICH) (11), and Swiss Law and Swiss regulatory authority's requirements (8). The CEC and regulatory authorities will receive the Annual Safety Report (ASR) and interim reports and be informed about the study stop/end in agreement with local requirements.

2.6 Declaration of Interest

This study is an MD-PhD thesis. The Sponsor supervises the MD-PhD student, who has been actively involved in the study design, protocol development, and documentation preparation for ethics submission. The student will be present at the study site and will participate in recruitment, data collection, study assessments, data analysis, and scientific writing for future publications.

2.7 Patient Information and Informed Consent

The PI (or his designee) will explain to each participant the nature of the study, its purpose, the procedures involved, the expected duration, the potential risks and benefits, and any discomfort it may entail. Each participant will be informed that participation in the study is completely voluntary, that he/she may withdraw from the study at any time, and that withdrawal of consent will not affect his/her subsequent medical assistance and treatment.

The participants will be informed that they can ask any questions and consult with family members, friends, their treating physicians, or other experts before deciding to participate in the study. The study participants will be given enough time (about 1 to 3 days). If the participant needs further time, it will be conceded (12).

The participants will be informed that authorized individuals other than their treating physician may examine his/her medical records.

All participants will receive a participant information sheet and a consent form, which will describe the study and provide sufficient information for them to make an informed decision about their participation. The formal consent of a participant, using the approved consent form, will be obtained before the participant is subject to any investigation procedure.

The participant should read, understand, and voluntarily agree before signing and dating the informed consent form. The consent form will be also signed and dated by the PI (or her/his designee). The signed consent form will be retained as part of the investigation records. The participant receives a copy of the signed document.

It is the responsibility of the PI (or her/his designee) to document in detail the information procedure and consenting process in the participant’s medical record or other suitable source data.

2.8 Participant privacy and confidentiality

The Sponsor and PI affirm and uphold the principle of the participant's right to privacy and that they shall comply with applicable privacy laws. The participants' anonymity shall be guaranteed when presenting the data at scientific meetings or publishing them in scientific journals.

Medical information obtained because of this study will be considered confidential. Disclosure to third parties will be prohibited.

Confidentiality will be ensured by utilizing a unique participant identification code consisting of two consecutive numbers starting from 01, as given by the REDCap software. Signed informed consent forms and participant enrolment logs will be kept strictly confidential to enable participant identification at the study site. The code list with the participants’ personally identifiable information will be kept strictly confidential at the study site, and only authorized personnel will have access to it during the trial and after its completion (13) (**See Section 12.2**).

For data verification purposes, authorized Sponsor, CA, or CEC representatives may require direct access to parts of the medical records relevant to the study, including participants’ medical history.

2.9 Early termination of the study

The Sponsor may terminate the study prematurely according to certain circumstances, for example:

- ethical concerns,
- insufficient participant recruitment,
- when the safety of the participants is doubtful or at risk, respectively,
- alterations in accepted clinical practice that make the continuation of a clinical trial unwise,
- early evidence of the benefit or harm of the experimental intervention
- evidence of inefficacy of the experimental intervention

2.10 Protocol amendments

Substantial amendments will be only implemented after approval by the CEC and CA.

Under emergency circumstances, deviations from the protocol may proceed without the authorities'

approval (CEC and CA) to protect the participants' rights, safety, and well-being. Such deviations will be documented and reported to the Sponsor and the CEC/CA immediately (within two days).

All non-substantial amendments will be communicated to the CEC in the ASR and to the CA as soon as possible. The ASR shall include any deviations from the protocol that may have affected the rights, safety, or well-being of the participants or the scientific integrity of the investigation.

2.11 Deviation from the CIP

Any deviations from the protocol will be recorded in a specific form in the eCRF and reported in the final study report. The sponsor, notified automatically by REDCap, will assess each recorded deviation's impact on the study results or the safety of participants. The protection of the participants and data quality and security must be guaranteed in all cases, and mitigation actions will be taken if needed.

3. BACKGROUND AND RATIONALE

3.1 Background and Rationale

T1D is an autoimmune condition resulting in an insulin deficiency and a life-long need for insulin replacement (14). Glycemic control in T1D remains a challenge, with only ~32% of adults in Switzerland (~20% of American adults) achieving an A1c target of <7%(15,16), despite the availability of modern insulin analogs(17), the improving accuracy of glucose monitoring(18), the widening use of ITT(5), and the emerging use of closed-loop systems(19). These new technologies have proven benefits in improved glycaemic outcomes and reduced hypoglycemia when compared with multiple daily injections (MDI)(20). However, excess mortality, weight gain, dyslipidemia, and cardiovascular complication rates remain higher in people with T1D (6,21).

The increase in the prevalence of overweight and obesity in people living with T1D has also been documented in recent studies(22,23). Many patients with T1D nowadays meet the metabolic syndrome's criteria. This combination of T1D with insulin resistance led to the concept of "double diabetes" (DD). Typically, these patients have higher HbA1c values, increased body weight, and receive higher insulin doses(24). In addition, epidemiological data indicate that people with T1D have lifespans ~11-13 years shorter(25) and experience cardiovascular disease (CVD) events on average more than a decade earlier than the general population(26). Various studies have identified CVD as the main cause of death in T1D(27,28) and worrisomely found that T1D confers substantial CVD risk even when conventional treatment targets are achieved(29). Diabetic kidney disease is also one of the most devastating complications of T1D and is strongly linked to CVD in this population(26).

Studies with adjunct therapies to intensified insulin treatments have been conducted to improve glucose control and reduce the risk of microvascular and macrovascular complications. Agents recently evaluated include GLP-1 RAs (30), dipeptidyl peptidase-4 (iDPP-4) (31), amylin analog (32), and sodium-glucose transporter-2 inhibitors (SGLT2i) (33). The GLP-1RAs and (GIP)/GLP1-RAs (or dual incretins) analogs have emerged as a promising therapy in T1D(30,34,35). These medications allow the reduction of plasma glucose with a very low risk of hypoglycemia (36). The principal mechanisms leading to reductions in plasma glucose concentrations are (i) glucose-dependent insulinotropic actions, (ii) suppression of glucagon hypersecretion except during episodes characterized by hypoglycemia, and (iii) a deceleration of gastric emptying, which is associated with marked effects on post-meal glycemic excursions (37). The GLP-1RA and (GIP)/GLP1-RAs, also induce early acute changes in appetite and satiety, a corresponding reduction in caloric intake, and weight loss (38,39).

The combination of GLP1Ras and GIP/GLP-1RAs with insulin therapy has been extensively evaluated in T2D (40,41). However, several randomized controlled clinical trials have also suggested a positive impact in T1D as adjunctive therapy to insulin (42–44). in a Metanalysis published in 2017, Wang et al. showed that GLP-1RAs in combination with insulin improve glycemic control and reduce insulin bolus dose and weight in T1D (45). In the same direction, The Lira Pump trial, a randomized, double-blinded, placebo-controlled trial, demonstrated that Liraglutide (a short-acting GLP1-RA) treatment reduced HbA1c, total daily insulin (TDI) dose, and body weight without increasing the risk of hypoglycemia in continuous subcutaneous insulin infusion (CSII)-treated patients with T1D and insufficient glycemic control (46)

The combination of insulin therapy with GLP-1RAs also showed promising results in mitigating postprandial excursions in T1D. A study by Sherr et al. evaluated pramlintide and liraglutide as adjuvant to automated insulin delivery in scenarios including unannounced meals. Results indicated meaningful mitigation in the postprandial excursion; liraglutide demonstrated the additional benefit of weight loss with insulin dose reduction (47). Similar findings were also found with exenatide in patients with T1D and AID (48). Recently, Karakus et al., in a retrospective observational study, combined GIP/GLP-1RA (tirzepatide) with the Control-IQ system in 11 obese adults with T1D over eight months(49). Results showed significant reductions in the TDI in the first two months, which lasted until the end of the observation period. However, results from prospective randomized studies with long-acting GLP1-RAs and (GIP/GLP-1RAs) as adjunctive agents to AID have yet to be presented. Further prospective studies are needed to evaluate the efficacy and safety of these therapies.

GLP-1RAs have demonstrated the reduction in major adverse cardiovascular events (MACE) outcomes in T2D in several large clinical trials (CTs) (50). In case of T1D, there is no current treatment holding both glycemic and cardiovascular indications despite the increased CVD risk in this population (26,51) GLP-1RAs and GIP/GLP1RAs may improve glucose control and potentially mitigate cardiovascular risk

in the T1D population.

We will conduct a prospective, parallel clinical trial to evaluate the safety and efficacy of AID systems combined with tirzepatide (GIP/GLP-1RA) in people with T1D on all time-in-range consensus criteria during the assessment period. The outcome of this study will provide unique data about the safety and efficacy of using GIP/GLP1-RAs as adjuvant therapy to AID.

3.2 Investigational Product (treatment) and Indication

Tirzepatide (*Mounjaro®*) is the first-class incretin mimetic with dual GIP and GLP-1 receptor agonism; its synthetic chemical structure is based on the GIP sequence consisting of 39-amino acid peptide but has been modified to include (i) substitution of the second amino acid with aminoisobutyric acid to avoid DPP-4 proteolysis and, (ii) addition of a C20 fatty di-acid acyl chain to enable non-covalent albumin binding and once-weekly subcutaneous dosing (52). Tirzepatide exhibits comparable binding affinity and potency at the human GIP receptor (GIPR) relative to the native GIP peptide. However, tirzepatide binds with 5-fold less affinity and is 13-fold less potent at the human GLP-1R than native GLP-1 (52).

The peptide is well absorbed after subcutaneous (SC) administration with a mean absolute bioavailability of 80%. Following an SC administration, tirzepatide plasma concentrations reach a peak serum concentration (Tmax) between 24 and 48 hours. The average half-life (t1/2) is ~5 days with an expected steady state within 4–5 weeks of therapy (53).

Tirzepatide bound in vitro highly to serum albumin (99%). In vivo metabolism and excretion studies with [14^{C}] tirzepatide showed that tirzepatide excretion is generated via proteolytic cleavage of the peptide backbone, β-oxidation of the C20 fatty acid moiety with and without taurine conjugation, and/or amide hydrolysis. Drug-related material was excreted via urine and feces (49% via urine, 35% via feces) (53,54).

Tirzepatide is currently authorized in Switzerland to treat adults with T2D or obesity in addition to diet and exercise. There is no current indication to use tirzepatide in T1D from the local authorities.

3.3 Preclinical Evidence

GLP-1 released from gut enteroendocrine cells controls meal-related glycemic excursions through insulin augmentation and glucagon secretion inhibition. GLP-1 also inhibits gastric emptying and food intake, maximizing nutrient absorption while limiting weight gain (55). Human GIP is a 42 amino acid protein that is secreted from K cells in the mucosa of the duodenum and jejunum and, similar to GLP1, also exerts its actions through a G protein-coupled receptor and as an incretin hormone potentiating insulin release from beta cells in a glucose-dependent manner (56).

The insulinotropic effect of GIP and GLP-1 is additive in healthy humans but is impaired in people with type 2 diabetes (T2D) (57) and T1D (58). Preclinical studies have revealed that GIP has protective effects on pancreatic beta cells' survival (59,60). Furthermore, GIP has been shown to have additional actions beyond its effect on insulin secretion, including the modulation of fatty acid-stimulated lipogenesis and lipid uptake, as well as the reduction of lipolysis (61). Additionally, eliciting effects on osteoclasts and osteoblasts involved in bone remodeling through their receptors expressed in bone tissue have also been reported (62). Consequently, GIP receptor (GIPR) agonism became an attractive pharmacotherapy agent to pair with GLP-1R agonists, especially since GIPR agonist treatment protects against illness-like behaviors manifested as anorexic and nausea phenotypes in mice that received GLP-1R agonists (61). These beneficial effects of a GIPR agonist help to tolerate drug delivery, enhance efficacy, and reduce emesis induced by GLP-1R agonists.

A non-clinical study demonstrated that GLP-1R/GIPR dual agonist, tirzepatide (10 nmoL/kg) daily for 14 days in diet-induced obese (DIO) male mice, reduced the food intake, with subsequent losses in body and fat tissue weight. In parallel, this study showed reductions in circulating leptin, triglycerides, free fatty acids, fed and fasting glucose, and insulin, as well as improved insulin tolerance, independently of the weight loss (63). In an intra-islet signaling study conducted by El et al., it was found that tirzepatide stimulates insulin secretion in mice predominantly through the GLP-1R, while in humans, it stimulates insulin secretion through both the GLP-1R and GIPR, and further enhances glucagon secretion and

somatostatin secretion(64). This provides an important advance in the understanding of tirzepatide pharmacology and highlights the differences in rodent versus human biology (64). Additional studies have reinforced the discoveries mentioned above by using tirzepatide. These studies have revealed that tirzepatide can enhance other metabolic functions such as raising blood flow to adipose tissues, improving glucose and lipid uptake, and increasing insulin sensitivity. These effects occur through the activation of receptors that are expressed in adipose tissues (61,65).

Beyond the metabolic effects, one study compared the effects of exogenous GIPR (300 nmoL/kg), GLP-1R (30 nmoL/kg), and GLP-1R + GIPR agonism on the development of atherosclerosis in the aorta of female ApoE*3-Leiden CETP transgenic mice every 2 days for 10 weeks. The study found that combined GLP-1R and GIPR mono-agonism attenuated aortic lesion development (66). When GIPR and GLP-1R agonists were co-administered, they further reduced lesion severity, coinciding with lower markers of inflammation and circulatory triglycerides suggesting a strong synergistic action (67). The potential cardiovascular benefits have also been tested in human cardiac AC16 cell lines by Taktaz et al. in (68). This study revealed that tirzepatide has beneficial effects on cardiac cells by positively modulating cardiomyocyte death, fibrosis, and hypertrophy in the presence of high glucose concentrations in vitro, which supports the potential benefit of tirzepatide against diabetes-related damage to cardiomyocytes (68).

In addition, another substitute of CVD as metabolic dysfunction associated with steatotic liver disease (MASLD) has been tested in rodent models, db/db, DIO mice, and/or diet-induced metabolic dysfunction-associated steatohepatitis (MASH). The chronic administration of GLP-1R/GIPR co-agonists exhibited greater weight loss, improvements in liver and blood lipid profiles, reduced liver fibrosis, and improved glucose control compared to controls (69). These preclinical data have been examined in a clinical setting through the SURPASS clinical trial program in T2D (70) and SURMOUNT clinical program for people living with obesity(71).

3.4 Clinical Evidence to Date

Tirzepatide is a first-in-class dual GIP and GLP-1 RA that is approved for use in adults with T2D in the EU (72), and USA (73). In patients with T2D, tirzepatide improved markers of β-cell function and insulin sensitivity to a greater extent than semaglutide (74), dulaglutide (75), and placebo (76). The glucose-lowering efficacy of tirzepatide in adults with T2D as evaluated in large, randomized, double-blind or open-label, phase III trials conducted globally (SURPASS 1–6), in Asia-Pacific (SURPASS-AP-combo; ≈ 83% of patients were of Chinese origin) and Japan (SURPASS J-mono and J-combo) (77). In the SURPASS trials, patients were randomized to tirzepatide 5, 10, or 15 mg (initiated at a dose of 2.5 mg once-weekly and increased by 2.5 mg every four weeks until the assigned dose was achieved), placebo or active comparators in addition to diet and exercise for 40 or 52 weeks. The primary endpoint was noninferiority or superiority evaluated for mean change from baseline in HbA1c at the study end, except in SURPASS J-combo where the primary endpoint was safety and tolerability (77).

In the SURPASS trials, tirzepatide improved glycaemic control and body weight in adults with inadequately controlled T2D, including when used as a monotherapy, as an add-on to oral hypoglycemic agents, or as an add-on to titrated basal insulin. Reductions in HbA1c levels were seen as early as week four, nearing the maximum about week 24; fasting serum glucose (FSG) levels followed a similar temporal pattern. Treatment with tirzepatide also improved 7-point self-monitored blood glucose profiles relative to both placebo and active comparators and resulted in mean 2-h postprandial glucose levels that were generally within the normal range (≤ 140 mg/dL) at the study end (78). Tirzepatide weight loss began by week four, did not plateau, was sustained for the duration of the trials, and was dose-dependent (78,79). These findings led to a significant increase in the number of patients achieving the target HbA1c proposed by diabetes clinical guidelines (80). Regarding safety and tolerability, tirzepatide was generally well tolerated as monotherapy or add-on therapy to oral hypoglycemic agents or basal insulin in adults with T2D in nine SURPASS trials, with a safety profile consistent with that of selective GLP-1Ras (81). In a head-to-head study that compares tirzepatide vs semaglutide (a potent long-acting GLP1RA); tirzepatide was non-inferior and superior to semaglutide for the mean change in the HbA1c level from baseline to 40 weeks with favourable tolerability and safety (82).

In TD1 the clinical evidence of tirzepatide is still insufficient. A retrospective proof of concept conducted by Kaan Akturk, et al. in (34) demonstrated that tirzepatide was associated with significant improvement in glycemic outcomes and reduction in weight without an increase in hypoglycemia or ketosis. Glycemic improvements were seen within the first three months. There was no clinically significant improvement

in HbA1C beyond three months, despite continuous weight reduction (dose-dependent). These data suggest that glycemic efficacy can be achieved in a short time frame with a 2.5 to 5 mg/week dose without further significant improvement with higher dosage and with adequate tolerability and safety. Consequently, randomized clinical trials are required to test the efficacy and safety of tirzepatide in T1D.

Furthermore, cardiovascular risk is also a concern in T1D. Epidemiological data indicate that people with T1D have lifespans ~11-13 years shorter and experience CVD events on average more than a decade earlier than the general population (83). Currently, no T1D therapy holds both glycemic and cardiovascular indications. Tirzepatide has demonstrated a beneficial effect on other cardiometabolic risk markers including lipid profiles and blood pressure, as well as improving liver fat content and reducing visceral and subcutaneous abdominal adipose tissue volume (84). Moreover, the effect of tirzepatide on MACE is currently being evaluated in SURPASS-CVOT (85). Since cardiovascular risk factors are increasing their prevalence among people with T1D and CVD is the leading cause of death in people with T1D (86), exploring the effect of tirzepatide on cardio-renal outcomes, lipids, and biomarkers of MASLD is also the special importance.

We plan to conduct a pilot trial to compare tirzepatide+AID and the standard of care (SoC)+AID in patients with T1D. This study will provide unique data about the safety and efficacy of using tirzepatide as adjuvant therapy to AID. This knowledge may significantly improve glucose control and represent a true paradigm shift in clinical practice re-directing the attention beyond insulin therapy.

3.5 Rationale for the dosage, route, and regimen

In this study, we will commence with the recommended initial dosage of tirzepatide (Mounjaro®), which is 2.5 mg administered subcutaneously once weekly for four weeks. The 2.5 mg dosage is for treatment initiation and is not intended for glycemic control (titration phase) (53).

After four weeks of dose escalation, the dosage will be increased to 5 mg once-weekly injected s.c. and kept stable for 12 weeks (treatment period). In case of intolerance to the medication, the dose can be scaled back. All participants will be encouraged to use a maximally tolerable dose of up to 5 mg/week (the lowest treatment dose of tirzepatide currently approved for T2D).

All patients will receive verbal and written education on using the tirzepatide (*Mounjaro®*) pen.

3.6 Explanation for choice of comparator (or placebo)

This study is a randomized parallel study comparing participants under tirzepatide+AID with AID alone (control) over three months. Participants under AID will continue with their home treatment according to international guidelines (14,87) and the criteria of their treating physician (14,86).

3.7 Risks / Benefits

The risks/benefits are detailed below. The planned study-related interventions involve similar risks to those faced by people with T1D in their daily lives and are less than the incidence of acute complications in patients with T1D reported by the Type 1 Diabetes Exchange Clinic Registry (88).

a) *Blood Drawing Risks*
The risks of drawing blood from a vein include temporary discomfort from the needle stick (common), bruising (common), excessive bleeding (unlikely), light-headedness (rare), infection (rare), and fainting (rare). The participant will be informed that the discomfort will be minimal, and no health concerns will be expected.

b) *Fingerstick Risks*
A fingerstick will require about one drop of blood to measure blood glucose. This standard method is used to obtain blood for routine hospital laboratory tests. Pain is not unusual at the time of lancing. In about 1 in 10 cases, a small amount of bleeding under the skin will produce a bruise. A small scar may persist for several weeks. The risk of local infection is less than 1 in 1000. This should not be a significant contributor to risks in this study, as fingerpick tests are part of the usual care for people with diabetes.

c) *Study procedure-related discomfort.*
Participants may feel some discomfort during height, weight, waist, and hip circumferences and blood pressure measurements, and during the electrocardiogram (ECG). All care will be taken to reduce discomfort.

d) *CGM Sensor Risks*
Participants using the CGM will be at low risk for developing a local skin infection at the site of the sensor placement. There is a risk of infection at the insertion site, which may cause swelling, redness, and pain. These occur infrequently, but oral and/or topical antibiotics can be used if infection occurs. There may be bleeding where the sensor is put in, and bleeding under the skin causes a bruise (1 in 10 risk). Study staff will verbally alert the participant that, on rare occasions, the CGM may break and leave a small portion of the sensor under the skin, which may cause redness, swelling, or pain at the insertion site. The participant will be further instructed to notify the study team immediately if this occurs.

e) *Risk of Device Reuse*
The study CGM system is intended for single use only. The study CGM system will be single-use only and will not be reused. The study phones (if applicable) and activity trackers may be reused during the study after cleaning the device using a hospital-approved cleaning procedure. The study blood glucose-ketone meter is labeled for single-patient use and will not be shared.

f) *Side-effects related to tirzepatide (Mounjaro®)*
The most recognized adverse reactions associated with tirzepatide (*Mounjaro®)* are gastrointestinal disorders, which are deemed to be dose-dependent. Nausea and vomiting are the most common (about 48.9% of the patients with the highest dose of 15 mg weekly) (89). Constipation, abdominal pain, diarrhea, and dyspepsia are also relatively common in clinical trials. The frequency of these adverse effects was more pronounced at the beginning of the treatment but decreased gradually as therapy continued (89). In this study, we will start with the recommended initial dosage of tirzepatide to improve the medication's tolerance. The dose will be gradually scaled up to 5 mg weekly, and in case of intolerance, the dose will be scaled back.

Tirzepatide (*Mounjaro®*) is a synthetic peptide that may lead to antibody formation, similar to other subcutaneously injected peptides. Participants treated with tirzepatide can develop antibodies and immune-related and hypersensitivity reactions, but their incidence is in general low and mostly mild to moderate in severity (90). If suspected, the study team will assess and refer the participant for appropriate care, and the medication will be discontinued. The study physician will contact the participant's physician.

Injection site reactions, such as rash, erythema, or itching, are common with GLP-1RAs and GIP/GLP1RAs. These reactions are most often transient and generally do not cause treatment discontinuation (91).

The possible association between tirzepatide (*Mounjaro®*) and the risk of pancreatitis has also been described. This association must be considered, especially in participants with several risk factors for pancreatitis, such as severe hypertriglyceridemia or alcohol intake (89,92). These participants will be excluded from the study (**See Section 7.1**). Other adverse effects of these drugs include headache and nasopharyngitis, but these effects do not usually lead to discontinuation of the drug. This medication has a rare risk of severe hypoglycemia, cholelithiasis, and cholecystitis (≤1%) (91,92).

An increase in heart rate has also been described with a dose-response relationship in the following order: Tirzepatide 15 mg > 10 mg > 5 mg(93). Participants with uncontrolled CVD and a personal history of arrhythmias will be excluded from this study (**See Section 7.1**). An ECG will be performed periodically during the study.

Tirzepatide is contraindicated in patients with a history or family history of medullary thyroid cancer and patients with multiple endocrine neoplasia type 2 (MEN-2) (53) (**See Section 7.1**).

Animal reproduction studies suggest there may be risks to the fetus from exposure to tirzepatide during pregnancy. Additionally, tirzepatide may reduce the effectiveness of oral hormonal contraceptives due to delayed gastric emptying, which is most pronounced after the first dose and diminishes over time. Female participants of childbearing potential using oral contraceptives should switch to a non-oral method (e.g., vaginal ring or hormonal patch) or use a barrier method for four weeks after starting tirzepatide and for four weeks after each dose escalation (**See annex 7** for study contraceptives guidelines).

g) *Risk of Hypoglycemia (Low Blood Glucose)*

As with any person with diabetes who uses insulin, there is always a risk of having low blood sugar (hypoglycemia) (88). GIP/GLP-1RA, such as tirzepatide (Mounjaro®), is known to improve insulin action and, therefore, may increase the risk for low blood glucose (**See Annexes 1 to 3** for the adjustment in the AID systems during the drug up-titration and treatment period). Symptoms of low blood glucose can include sweating, jitteriness, and not feeling well. There is also the possibility of fainting or seizures (convulsions), brain damage, or death with level 3 of hypoglycemia (80). The participants will be strictly monitored (as a part of the clinical study) for these symptoms. The frequency of hypoglycemia should be no more and possibly less than it would be as part of daily living. Low glucose levels can be quickly resolved with a hypoglycemic treatment (fast-acting carbohydrates). Severe low blood glucose may require the administration of intranasal glucagon (*BAQSIMI*®) or vial injection (*GlucaGen*® *HypoKit*®) and/or have emergency services to help raise the participant's blood glucose level (**See Annex 4 and 10** for the hypoglycemia risk mitigation plan and hypoglycemia classification).

h) *Risk of Hyperglycemia (High Blood Glucose)*
Hyperglycemia and ketone formation are always a recognized risk in people with T1D. Hyperglycemia and ketonemia could occur if insulin administration is attenuated or suspended for an extended period (88). Hyperglycemia usually does not cause obvious symptoms, but participants may become thirsty, fatigued, or have higher glucose levels in their urine. In severe cases of hyperglycemia, diabetic ketoacidosis (DKA) or coma may occur. Hyperglycemia leading to DKA can lead to renal failure (kidney failure), cardiac arrhythmia (irregular heartbeat), myocardial infarction (heart attack), rhabdomyolysis (muscle breakdown), and even death (94). A serious effect from hyperglycemia is not expected to occur in any study participant, as we will be monitoring blood glucose levels frequently (**See Annex 5** for Guidance on checking for ketones).

i) *Whole-body composition using bioelectrical impedance analysis (BIA)*
During the study, we will assess the body composition using a BIA (*Seca mBCA 514*®). BI measures body composition based on the electrical conductive properties of the body(95). The evaluation is noninvasive, quick, and needs little technical skills and preparation by the individual. The BIA has an accuracy comparable with the gold standard when is performed under standardized conditions(96). The participants will be instructed about the technique before the procedure.

j) *Hepatic elastography (FibroScan®)*

Liver steatosis and fibrosis will be assessed by vibration-controlled transient elastography (VCTE) utilizing *FibroScan*®. The technique uses an ultrasound transducer probe to create an elastic shear wave through vibrations of mild amplitude and low frequency (50 Hz), which are transmitted through liver tissue, and provide a liver stiffness measurement (LSM) and controlled attenuation parameter (CAP). The LSM is expressed in kilo pascals (kPa) that correlate with the fibrosis stage and the CAP value is expressed in decibels per meter that correlates with liver steatosis grade(97).*FibroScan*® assessment is noninvasive, safe, and easy to perform. Before the procedure, the participants will be instructed about the technique.

k) *Confidentiality*
There is a risk of confidentiality breach. The study team will capture the participants' information via a secure web application (REDCap). Each participant will be assigned an identification number, which will be used to code and identify all participant records. All participant records will be stored at the study site in a locked in a locked place and all relevant computer study files will be input into staff computers, which are password-protected. Data access will be limited to selected study personnel. (**See Chapter 12**).

Study results may be presented in posters, abstracts, oral presentations, or publications at academic meetings or in journals. Participant identification will not be disclosed in public reports. A study participant may access his/her protected health information at any time by requesting said information in writing from the investigator. The study team has been trained in GCP and

will maintain confidentiality and protect health information.

3.8 Justification of choice of the study population

This study will be conducted on individuals living with T1D. Only people with T1D using a Swiss-approved AID system will be included. No vulnerable populations, such as neonates, pregnant women, children, prisoners, institutionalized individuals, or others who may be considered vulnerable populations, will be included in this study. More details about the eligibility criteria and recruitment of the participants are described in the following sections (**See Chapters 7 and 11**).

4. STUDY OBJECTIVES

4.1 Overall Objective

i. This study aims to evaluate the safety and efficacy of tirzepatide (*Mounjaro®*) 5mg as adjunctive therapy to AID in adults with T1D in comparison with the SoC.

4.2 Primary Objective

i. Evaluate the efficacy of tirzepatide (Mounjaro®) 5mg as adjunctive therapy to automatic insulin delivery (AID) system on continuous glucose monitor (CGM)-based percent time in the target range (TIR) in adults with T1D.

4.3 Secondary Objectives

i. Evaluate the efficacy of tirzepatide (Mounjaro®) 5 mg as adjunctive therapy to automated insulin delivery (AID) on CGM-based glucose metrics, HbA1c, and insulin delivery in adults with type 1 diabetes (T1D).
ii. Evaluate the efficacy of tirzepatide (Mounjaro®) 5 mg as adjunctive therapy to AID on anthropometric measures and body composition in adults with T1D.
iii. Evaluate the efficacy of tirzepatide (Mounjaro®) 5 mg as adjunctive therapy to AID on cardio-renal outcomes in adults with T1D.
iv. Evaluate the safety of tirzepatide (Mounjaro®) 5 mg as adjunctive therapy to AID in adults with T1D.

4.4 Safety Objectives

The study aims to assess preliminary data about the safety of the investigational medical product (IMP) (tirzepatide [*Mounjaro®*]) 5mg and its tolerability in terms of incidence of gastrointestinal side effects, hypoglycemia episodes, severe persistent hyperglycemia with ketosis, or DKA, serious adverse events (SAE) with a possible or more significant relationship to the use of the IMP (including expected and unexpected adverse drug reactions), other serious adverse events not related to the use of the IMP, and medical events of special interest (MESI) (**See Section 5.3**).

5. STUDY OUTCOMES

The primary endpoint will be evaluated by computing TIR over the 12 weeks after dose-escalation (See the treatment period in Table 1). Group A: tirzepatide+AID; Group B: SoC+AID (See Figure 1).

Primary Outcome

Mean difference in 24/7 CGM percentage time spent between 3.9 and 10 mmol/L (TIR) between groups at week 16 of treatment.

Secondary Outcomes

Secondary endpoints will be tested hierarchically to maintain the type I error at 5% in the following order

Mean difference between groups at week 16 of treatment in

Glycated Hemoglobin (HbA1C)
CGM-measured TIR between 0700 and 2300 (TIR_{7-23})
CGM-measured time in the tight range (TTR, 3.9 – 7.8 mmol/L)
24/7 CGM-measured percent time >10.0 mmol/L
24/7 CGM-measured percent time >13.9 mmol/L
24/7 CGM-measured percent time <3.9 mmol/L
24/7 CGM-measured percent time <3.0 mmol/L
24/7 Total Daily Insulin (TDI)

Exploratory Outcomes

Mean difference between groups at weeks 8, 12, and 16 of treatment in

CGM-measured TIR between 0700 and 2300 (TIR7-23)
24/7 CGM-measured percent time >10 mmol/L
24/7 CGM-measured percent time >13.9 mmol/L
24/7 CGM-measured percent time <3.9 mmol/L
24/7 CGM-measured percent time <3.0 mmol/L
CGM-measured glucose variability measured with the coefficient of variation (CV)
24/7 Total Daily Insulin (TDI)

24/7 Total Daily Bolus (TDBo)

Mean difference between groups at week 16 of treatment in

Anthropometric-Body composition:

- Body weight
- Waist circumference
- Hip circumference
- Waist-to-hip ratio
- % fat mass
- Fat mass index (FMI)
- Visceral adipose tissue (VAT)
- Lean mass index (LMI)
- Appendicular lean mass index (ALMI)

Cardio-Renal:

- Low-density cholesterol lipoprotein (LDL)
- High-density cholesterol lipoprotein (HDL)
- Cholesterol non-HDL
- Triglycerides (TG)
- TG-glucose index in fasting.
- Systolic and diastolic blood pressure
- Urine albumin to creatinine ratio (uACR)
- Hepatic steatosis and fibrosis assessed by FibroScan (CAP-value and LSM)
- MASLD biomarkers, hepatic steatosis index (HIS), and fibrosis score (FIB-4).

Other Outcomes of Interest

- Describe reported eating behavior and food intake (using the SNAQ application).
- Describe patient-reported outcomes: DTSQc (baseline)/DTSQc (last visit-end of treatment)

Safety Outcomes

- Occurrence of clinically significant hypoglycemia and severe hypoglycemia events (see **Annex 10** for hypoglycemia classification).
- Incidence of initiation of rescue therapy for severe persistent hyperglycemia and diabetic ketoacidosis episodes.
- Incidence of gastrointestinal side effects.
- Dehydration or renal acute events.
- Adjudicated hepatic or pancreatic AEs.
- Incidence of allergic and hypersensitivity reactions.
- Mean change in heart rate from baseline to 16 weeks.
- Treatment-emergent adverse events (TEAEs).
- Early discontinuation of study drug due to AEs.

Docusign Envelope ID: 11555059-DCA3-47FE-9701-55DBADEB3B9E

6. STUDY DESIGN

6.1 General study design and justification of design

This randomized, parallel, open-label study evaluates the safety and efficacy of tirzepatide (*Mounjaro*®) 5mg as adjunctive therapy to AID in adults with T1D. The study consists of an eligibility assessment, screening, and randomization, followed by two weeks of a baseline (run-in) period, where participants will be requested to use their SoC+AID, wear a study activity tracker (Fitbit sense 2), and log meals using the SNAQ application (98). After baseline, participants in **Group A** will undergo an up-titration phase with 2.5 mg SC of tirzepatide once a week to assess drug-related tolerability. Participants in **Group B** will continue with their SoC+AID. After the four weeks of titration phase, participants assigned to Group A will hold stable adjunctive doses of tirzepatide (5 mg SC once a week) for up to 12 weeks. During the up-titration and assessment phases, participants will continue using the activity tracker and SNAQ app as instructed by the study team (**See Figure 1**). The primary and secondary outcomes will be assessed at 16 weeks of treatment and safety and exploratory endpoints will be evaluated at weeks 8, 12, and 16 (**See Chapter 5**).

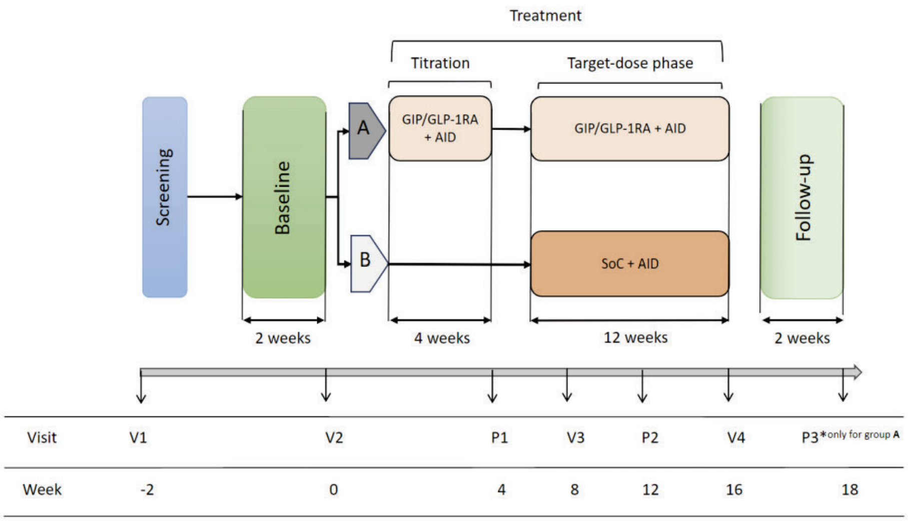

Figure 1. Study Design.

6.2 Methods of Minimizing Bias

6.2.1 Randomization

Randomization will be performed after checking the quality of the baseline data. Participants will be randomly allocated 1:1 to tirzepatide+AID or SoC+AID using the minimization method (using *Minimpy*® software, version 0.3), considering the values of the following variables: BMI (<27 and ≥27 kg/m^2), and HbA1C <7.5 and ≥7.5%).

6.2.2 Blinding procedures

Not applicable

6.2.3 Other methods of minimizing bias

Not applicable

6.3 Unblinding Procedures (Code break)

Not applicable

7. STUDY POPULATION

7.1 Eligibility criteria

7.1.1 Inclusion criteria

Participants fulfilling all the following inclusion criteria are eligible for the investigation:

1) Participants with diagnosed T1D for at least 12 months.
2) Aged between 18 to 65 years old (inclusive).
3) Currently on AID therapy for at least three months.
4) HbA1C ≥6.5% and ≤10%.
5) BMI ≥23 kg/m^2.
6) Willing to use once-weekly tirzepatide for at least 16 weeks (including up-titration)
7) Willing to wear a Dexcom G7 Sensor and share devices (AID) data uploads.
8) Willingness not to start any new non-insulin glucose-lowering agent during the trial (including metformin/biguanides, pramlintide, DPP-4 inhibitors, sodium-glucose cotransporter 2 inhibitors [SGLT2 inhibitors], and nutraceuticals).
9) A stable weight (± 5%) in the last 90 days or more before the screening and agree to not initiate a diet and/or exercise program during the study to reduce body weight other than the lifestyle and dietary measures for diabetes treatment.
10) Females with childbearing potential and males (if apply) must be willing to use reliable contraceptive methods (for the contraceptives study guidelines. **See Annex 7**)
11) An understanding and willingness to follow the protocol and signed informed consent.

7.1.2 Exclusion criteria

The presence of any of the following exclusion criteria will lead to the exclusion of the participant:

<u>Medical Conditions.</u>

1) History of diabetic ketoacidosis requiring hospitalization in the past six months.
2) History of severe hypoglycemic event (Level 3, defined as seizure or loss of consciousness) in the past six months.
3) Uncontrolled Diabetic retinopathy or maculopathy
4) Severe gastroparesis.
5) Less than 12 months of insulin treatment.
6) Estimated glomerular filtration rate (eGFR) lab value below 30 mL/min/1.73 m^2 by the CKD-EPI formula(99).
7) Pregnancy or intention to become pregnant during the trial (**See annex 7**).
8) Currently breastfeeding or planning to breastfeed.
9) Currently uncontrolled seizure disorder.
10) History of allergy to GLP-1RAs/GIP-RA, or its excipients.
11) Personal or family history of multiple endocrine neoplasia type 2 (MEN-2) or medullary thyroid carcinoma.
12) Screening calcitonin above or equal to 35 ng/L.
13) Planned any surgery during the study duration.
14) Have uncontrolled hypertension (systolic BP above or equal to 160 mmHg and/or diastolic BP above or equal to 100 mmHg). If a participant is on anti-hypertensive therapies, doses must be stable for 30 days before screening. For participants with uncontrolled hypertension at the screening visit, antihypertensive medication may be started or adjusted.
15) Personal history of one of the following cardiovascular conditions (within 2 months before the screening): acute myocardial infarction, cerebrovascular accident (stroke), unstable angina, or hospitalization due to congestive heart failure (CHF).
16) Conditions that may increase the risk of induced hypoglycemia, such as CHF with NYHA Functional Classification III or IV or adrenal insufficiency.
17) Have a history of documented human immunodeficiency virus (HIV) infection.
18) Have an uncontrolled cardiac arrhythmia based on an electrocardiogram (ECG) at the screening time and investigator’s discretion.
19) Cystic fibrosis.
20) Patient with a history of gastric bypass (bariatric) surgery, sleeve gastrectomy, or restrictive bariatric surgery, such as *Lap-Band®* or gastric banding.
21) Uncontrolled thyroid disease as judged by the investigator

22) Serum triglycerides >5.7 mmol/L (500 mg/dL) at the screening. If a participant is on lipid-lowering therapies, doses must be stable for 30 days before screening.
23) Personal history of acute or chronic pancreatitis. A participant with a history of acute pancreatitis caused by gallstones may be included in the study if the participant has had a cholecystectomy to resolve the problem.
24) Acute or chronic hepatitis other than MASLD.
25) Have a history of symptomatic gallbladder disease within the past 2 years (unless the participant has had a cholecystectomy to resolve the problem).
26) History of malignancy requiring chemotherapy, surgery, or radiation (other than basal or squamous cell skin cancer, in situ carcinomas of the cervix, or in situ prostate cancer) in the previous five years.
27) Active or unstable major depressive disorder (MDD) or other severe psychiatric disorder (such as known drug or alcohol abuse, diagnosed eating disorder, or any other uncontrolled psychiatric disorder) that, in the investigator's opinion, may preclude the participant from following and completing the protocol.

<u>Prior/Concomitant Therapy:</u>

28) Treatment with non-insulin glucose-lowering agents other than metformin (on a stable dose 30 days before the study)
29) Weight loss medications in the past three months.
30) Participants who are anticipated to receive, are receiving or have received within three months before the screening (>2 weeks and ≥10mg prednisolone-equivalent) chronic systemic glucocorticoid therapy (excluding topical, intraocular, intranasal, single intraarticular injection, or inhaled preparations).
31) Have current treatment with (or history of, within three months before screening) medications that may significantly affect glucose metabolism.
32) Use of investigational drugs within five half-lives before screening.

<u>Other exclusions</u>

33) Participation in another study with an investigational drug within the 30 days preceding and during the present study.
34) Current enrollment in another clinical trial unless approved by the investigator of both studies and if the clinical trial is a non-interventional registry trial.
35) Have evidence of a significant active, uncontrolled medical condition or a history of any medical problem capable of constituting a risk when using the study devices or interfering with following study procedures or the interpretation of data, as judged by the study physician at screening.
36) The enrolment of the investigator, his/her family members, employees, and other dependent persons.

The study physician/investigator will assess each eligibility criteria and decide to include the participant in the trial (ICH GCP 4.3.1). This decision will be documented before the participant receives the first study intervention.

7.2 Recruitment and screening

Participants will be recruited at the investigator’s clinic. Recruitment flyers targeting patients will be placed in waiting rooms and other suitable locations. Interested people can connect directly with the study team to express their interest in participating. The study team will perform a short interview based on the “recruitment checklist” on the phone or during a clinical visit to evaluate their eligibility. This data is not recorded in the electronic case report form (eCRF) since eligible and motivated individuals will be invited to a screening visit. During this visit, open questions related to the study will be clarified. After signing the informed consent form, eligibility will be assessed. Then, baseline data is collected (these will be included in eCRF). For more information about the screening procedures **see Section 9.3**.

Participants will receive compensation of CHF 200 for their participation, split into 50 CHF for each on-site visit. Travel fees (public transport 2nd class and/or kilometers driven by car, including parking fees) will be reimbursed separately, with an upper limit of about 50 CHF per visit.

7.3 Assignment to Study Groups

Randomization will be done by an independent person (not belonging to the study team) after confirming the quality of the data collected at baseline. We will perform the randomization at a ratio of 1:1 (tirzepatide+AID=21; SoC+AID=21), with the minimization method (100) using *Minimpy*® software,

version 0.3, considering the values of the following variables: BMI (<27 and ≥27 kg/m^2) and HbA1C <7.5 and ≥7.5%).

7.4 Criteria for withdrawal/discontinuation of participants

Participation in this research is voluntary. Participants may withdraw at will at any time. When withdrawing from the study, the participant should let the research team know that he/she wishes to withdraw. The participant is not required to provide a reason.

The investigator must stop the study intervention for the individual participant if any of the following criteria are met:

- Withdrawal of informed consent
- Confirmed pregnancy.
- Actively trying to become pregnant.
- Developing an allergic reaction to tirzepatide.
- If the inclusion and exclusion criteria are no longer met.
- Non-compliance with the study procedures, or
- At the investigators' judgment due to safety concerns (**See Subsection 9.4.1** for the definition of adverse events and other safety-related events).

After withdrawal, the participant will be given instructions on how to safely stop using the IMP and, eventually, on how to return to the previous treatment regimen correctly and safely. Instructions are also given on who to contact if any questions or concerns arise after study withdrawal. Any data collected until study withdrawal will remain coded for the analysis.

Withdrawn participants will not be replaced. However, at the investigator's discretion, re-screening is allowed within the recruitment period to complete 42 participants. Further information about the follow-up procedures is available in **Subsection 9.2.5**.

8. STUDY INTERVENTION

8.1 Identity of Investigational Products

8.1.1 Experimental Intervention

In this study, the IMP is tirzepatide (*Mounjaro®)*. This medication has marketing authorization in Switzerland. Tirzepatide (*Mounjaro®*) is a dual glucose-dependent insulinotropic polypeptide (GIP) receptor and glucagon-like peptide-1 (GLP-1) receptor agonist.

The recommended starting dosage is 2.5 mg injected SC once weekly. After four weeks, the dose could be increased to 5 mg injected SC once weekly until an upper limit dosage of 15 mg SC once weekly (in 2.5 mg increments after at least four weeks on the current dose) (53). For this study, the target dose is 5 mg, which is the minimum approved dose for glucose control.

8.1.2 Control Intervention (standard/routine/comparator treatment)

The participants in the control group will be requested to continue to use their (automated) insulin-related standard of care (SoC) and concomitant medication (if it does not represent an exclusion criterion).

8.1.3 Packaging, Labelling, and Supply (re-supply)

The IMP (tirzepatide) will be administered in its primary package. Tirzepatide (Mounjaro®) is a clear, colorless to slightly yellow solution available in cartons containing four pre-filled single-dose vials as follows: 2.5 mg, 5 mg, 7.5 mg, 10 mg, 12.5 mg, or 15 mg per 0.5 mL in a single-dose pen or single-dose vial.

Tirzepatide will be labeled in all required local languages according to annex 13 from Eudralex-Good Manufacturing Practice (GMP). Labeling will be performed by authorized personnel.

Participants will be provided with insulated bags containing cooling gel packs to transport the tirzepatide from the site to home.

The study team will have a stock of tirzepatide (for supply and re-supply) at the clinical recruitment site (**See Subsection 8.1.4**).

8.1.4 Storage Conditions

Tirzepatide (*Mounjaro®*) will be stored in a secure refrigerator at 2°C to 8°C (36°F to 46°F) at the study site and in the original carton to protect from light. The temperature log will be monitored at the site, and any temperature fluctuation will be reported as a deviation (in this case, the IMP is not given to the participant and is kept until destruction). If needed, each single-dose pen or vial can be stored unrefrigerated at temperatures not exceeding 30ºC (86ºF) for up to 21 days. Tirzepatide should not be frozen or used if it is frozen.

The principal investigator and the study team will be responsible for confirming that appropriate temperature conditions have been maintained during transit for all study treatments received and that any discrepancies are reported and resolved before using the study treatment. All the provided treatments will be stored in a secure (locked), environmentally controlled, and monitored (manual or automated) place following the labeled storage conditions per the manufacturer. Access will be limited to the investigators and authorized study members. All the participants will be instructed about the storage and handling conditions to be fulfilled at their place. Refer to the Swiss product information for detailed information on the handling and safety of *Mounjaro®* (9)

8.2 Administration of experimental and control interventions

8.2.1 Experimental Intervention

The IMP (tirzepatide) is administered weekly by the participant at any time of the day, with or without meals. It should be injected subcutaneously in the abdomen, thigh, or upper arm, rotating the injection sites for each dose. A maximum dose of 5 mg SC weekly will be administered as a treatment dose (after the titration phase with 2.5 mg SC/week), and all participants will receive verbal and written education on tirzepatide pen use(53).

8.2.2 Control Intervention

The control intervention consists of the (automated) insulin-related SoC and concomitant medication (if it does not represent an exclusion criterion). Participants in the control group will continue with their

home treatment according to international guidelines and the criteria of their treating physician(14).

8.3 Dose modifications

In this study, participants will start with an initiation dose of 2.5 mg weekly, and after four weeks, they will receive a stable dose of 5 mg weekly. In case of intolerance to the medication, the dose can be scaled back. However, the tirzepatide dose escalation scheme has been designed to minimize the development of GI symptoms. During the dose escalation period, every effort should be made by the investigator to escalate and maintain participants on the corresponding tirzepatide dosage (maximally 5mg/week). After the stabilization, the dose modification for tirzepatide is not permitted. **See Annex 8** for the plan to mitigate gastrointestinal symptoms.

If a dose is missed, the participant will be instructed to administer tirzepatide as soon as possible, within four days (96 hours) after the missed dose. If more than four days have passed, the missed dose should be skipped, and the next dose should be administered on the regularly scheduled day. The day of weekly administration can be changed (if necessary) if the time between the two doses is at least three days (72 hours).

8.4 Compliance with Study Intervention

The IMP compliance will be determined by the following:

- IMP administration data will be recorded by the participant weekly in a logbook and reviewed by the investigator at each on-site study visit while the participant is still present. If necessary, the participant should be asked to fill in any missing answers.
- If a dose is missed, the missed dose will be recorded as "not taken." A participant will be considered non-compliant if he or she is judged by the investigator to have intentionally or repeatedly taken less than the prescribed amount of medication.
- In addition to the assessment of a participant's compliance with the IMP administration, other aspects of compliance with the study procedures will be assessed at each visit based on the patient's adherence to the visit schedule, completion of the medication logbook, and any other parameters the investigator considers necessary.

Participants considered to be poorly compliant with their medication and/or the study procedures will receive additional training and instruction as required. They will be reminded of the importance of compliance. At the end of the study, treatment compliance will be defined as taking at least 75% of the required doses of the study drug.

8.5 Data Collection and Follow-up for Withdrawn Participants

The medical follow-up of prematurely withdrawn or dropped-out participants from the investigation is described in **Subsections 9.2.5 and 9.2.6**.

8.6 Trial-specific Preventive Measures

A serum pregnancy (serum beta-Hcg) test will be performed for all female participants of childbearing potential at the screening visit as part of the eligibility assessment. The result must be available before randomization and the first injection of the study drug. Additional pregnancy in urine tests will be repeated during the study.

If pregnancy occurs after inclusion and during the ongoing study, it will be reported to the Sponsor within a maximum of 24 hours. Consequently, the pregnant participant will be withdrawn from the study (**See Annex 7**).

In addition, the study team will encourage the participants to obtain glucagon Intranasal (*BAQSIMI®*) or vial injection (*GlucaGen® HypoKit®*) through prescription. All participants must have glucagon products as part of the standard of diabetes care. The participant and their caregiver (if any) must be trained on using glucagon products (**See Annex 4**, hypoglycemia risk mitigation plan).

The study participants will receive a ketone meter (**See Annex 5** for Guidance on checking for ketones).

The use of any rescue medication and concomitant medications will be recorded in the eCRFs.

8.7 Concomitant Interventions (treatments)

Section 7.1 describes the medications not allowed before and/or during the study. **Section 8.6** and **Annex 4** describe rescue treatments and relevant interventions for hypoglycemia. Participants will be encouraged to continue their chronic treatment and medications if these do not fall within the exclusion

criteria. Concomitant medications will be recorded in the eCRFs.

8.8 Study Drug Accountability

Only the study team can provide the IMP (tirzepatide). Study staff will regularly assess whether the patient is correctly administering the IMP and storing it according to the provided instructions.

The study site must report the dispensing and return of the IMP (including the unique patient number [UPN], date, vials dispensed/returned, and batch number) on an IMP accountability log. The study's site clinical coordinator will sign this log, which is stored in the investigator's site file (ISF) and checked by the clinical research associate (CRA).

8.9 Return or Destruction of Study Drug

Participants will be instructed to dispose of the pens in their original boxes (as there is no risk of puncture) and return all used pens and boxes to the study center during their next on-site visit. The returned used pens will be reconciled with the information recorded in the participant's logbook for IMP accountability and compliance evaluation. At the study site, all used pens will be safely disposed of per local regulations.

Unused or partly unused investigation product must be checked and reconciled with the participant's logbook. Unused, partly unused, or expired investigation products must be destroyed at the site according to local guidelines but only after the inspection of the CRA and receipt of instructions from the Sponsor. The destruction should be documented using the IMP accountability log.

8.10 Study accessories

8.10.1 CGM system

The Dexcom G7 CGM system, Dexcom Suisse GmbH, Allmendstr. 18, 6048 Horw, Switzerland (CE Cert. No: CE 591560), is a commercially available device that will be used in the study to collect CGM values. The sensor and transmitter are integrated in this model.

Data from a Dexcom G7 CGM system will be collected and used as the reference for blood glucose levels. The Dexcom G7 CGM system is a CE-marked device indicated for the management of diabetes mellitus in people aged two years and older. To standardize and harmonize glucose data collection independently of the AID system, all participants will be provided with study CGMs (Dexcom G7). Participants must wear the Dexcom G7 sensor in addition to their existing sensor connected to the AID system (unless Dexcom G7). Data from the study sensor will not be blinded and will be used solely for research purposes. The CGM sensor should be replaced at least once every ten days. The investigator will provide the necessary sensors (for re-supply) at visits 2 and 3.

Coded data (i.e., not containing identifiable patient information) from the CGM and AID system will be continuously or intermittently uploaded to a cloud-based platform accessible only to study team members.

The (study) CGM values are for monitoring only and should not be used for diabetes management during the study.

8.10.2 Ketone and Glucose Meter

The Abbott FreeStyle Optimum Ketone monitoring system is intended for in vitro diagnostic use for the quantitative measurement of β-ketone (β-hydroxybutyrate) in fresh capillary whole blood samples. The meter uses single-use test strips to measure the concentration of β-ketones in fresh capillary and venous whole blood samples. Blood ketone strips measure β-Hydroxybutyrate are considered as 'Gold Standard' in the management of Diabetic Ketoacidosis. The necessary Ketone meters (and corresponding material (test strips, lancing device, and lancets) will be provided (free of charge) by the investigator at visits 2 and 3.

The participant also can use the same device to self-monitoring blood glucose (SMBG) according to the medical routine. The SMBG is a routine process part of the SoC. These values are used to verify the measured CGM data and to calibrate the CGM sensor if necessary.

8.10.3 Physical activity trackers

The Fitbit sense 2, Fitbit LLC, 199 Fremont Street, 14th Floor, San Francisco, CA 94105 USA is a commercially available device that will be used in the study to collect activity data. The Fitbit sense 2

uses state-of-the-art technology to track sleep and heart monitoring. Patients will be equipped with a Fitbit sense 2 at visit 1 and will wear the device throughout the study. The Fitbit sense 2 is used to collect additional physiological data:

- Heart rate and heart-rate variability
- Oxygen saturation
- Respiration rate
- Stress level
- Sleep stages
- Steps
- Skin temperature
- All-day body-response

8.10.4 SNAQ application

During the study, the participants will document their food intake using the SNAQ App installed on a compatible iPhone/Android phone. The app will be installed and connected by the study team. The participants will be instructed to set up the CGM sensor and the SNAQ App on the phone. This instruction will either take place in person or via a phone call. After setting the CGM sensor, the Participants will be instructed to document their food intake using the SNAQ App for five days before each on-site study visit, starting during the run-in period (before visit 2), 3, and 4. This procedure is simple: the participant can introduce the data by taking a picture of the food/beverage, scanning a barcode (if available), or manually entering a particular food item into the app. This app has been used in previous clinical trials.

8.10.5 Smartphones

iPhone/Android phones compatible with the Dexcom G7, Fitbit App, and SNAQ App will be used in this study.

Study participants will be equipped with a study smartphone with pre-installed apps to connect to the wearable devices and instructed for their use. A pseudonym will be used for all created study-specific accounts.

The participant also can use her/his phone if it is compatible with Dexcom G7 and the study apps. The apps will be installed and connected by the study team.

The smartphones will only be used to synchronize the data measured with the wearable devices and SNAQ App. The synchronization will be done with the support of the study site at each study visit and will be checked by a study's member every week to guarantee the quality of the data collection. At the end of the study, the participants will return the study smartphones, physical activity trackers, and glucose-ketone meters.

8.10.4 Accountability

The study site must report the dispensing and return of the study accessories on the study accessories accountability log. Any damaged or unusable study accessory will be documented on this log. The investigator or designee must notify the Sponsor of any damaged or unusable study accessories.

The study accessories accountability log will be signed by the study coordinator of the clinical study site. This log is stored in the ISF and checked by the CRA.

Usage of the study accessories (including serial number) will be recorded in the eCRFs.

9. STUDY ASSESSMENTS

9.1 Study flow chart(s) / table of study procedures and assessments.

All visits are consigned in Table 1 (**See pages 12-14**).

9.2 Assessments of Outcomes

9.2.1 Assessment of primary outcome

The primary outcome of the study is the Mean difference in 24/7 CGM percentage time spent between 3.9 and 10 mmol/L (TIR) between groups at week 16 of treatment. The primary endpoint will be evaluated by computing TIR over the 12 weeks after dose-escalation (See the treatment period in Table 1). Group A: tirzepatide+AID; Group B: SoC+AID (See Figure 1).

9.2.2 Assessment of secondary (exploratory) outcomes

The secondary and exploratory outcomes will be assessed as follows:

The secondary outcomes based on CGM metrics and AID systems will be collected as described for the primary outcome and as repeated measurements from baseline to weeks 4, 8, and 16 of escalated treatment.

The anthropometric measurements and the record of the vital signs (blood pressure and heart rate, measured by pulse) will be performed according to the protocols for the World Health Organization's STEPwise approach to Surveillance (STEPS) (WHO 2017) (101). Height and body weight will be measured with *Seca Digital Ultrasonic Measuring Rod®* (Seca, Germany), with an accuracy of 0.1 kg. Waist circumference (WC) will be measured with a fiberglass anthropometric tape at the intermediate point between the lower edge of the last rib and the iliac crest in the horizontal plane (approximately 2.54 cm above the navel). The hip circumference will be measured around the widest part of the buttocks, making sure that the tape is snug but not tight, and is parallel to the floor. Body mass index (BMI) will be auto-calculated with the formula weight/(height)^2 in kg/m^2.

Vital sign measurements will be taken before obtaining an electrocardiogram (ECG) tracing and collecting blood samples for laboratory testing. The participant should sit quietly for five minutes before taking vital signs measurements. For each parameter, two measurements will be taken using the same arm, preferably the nondominant arm. The recordings should be taken at least one minute apart. Each pulse and blood pressure measurement will be performed sitting and recorded in the eCRF. Blood pressure will be taken with an automated blood pressure instrument.

Body composition: bioelectrical impedance analysis will assess global and regional fat mass (*Seca mBCA 514®)*. For this study, fat mass percentage (FM%), fat mass index kg/m^2 (FMI [total FM/height^2]), visceral adipose tissue liter ([l]VAT), lean mass index kg/m^2 (LMI [total LM/height^2]) and appendicular lean mass index (ALMI [total LM/height^2]) will be considered relevant because they have been associated with an elevated risk of cardiometabolic diseases and mortality (102,103).

The laboratory analysis (for assessing eGFR, uACR, and lipids) will be taken according to the study schedule (**See Table 1**). The samples will be processed in the study site's central laboratory and reported in the International System of Units (the central laboratory reference ranges will determine the high and low laboratory limits). The HbA1c level will be measured using the DCA Vantage or a similar point-of-care device/local lab. The CKD-EPI equation will be used by the central laboratory to estimate and report eGFR(99).

MASLD will be assessed by laboratory analysis (HSI, FIB-4) and hepatic Elastography (*FibroScan®*).

The overall diabetes satisfaction will be assessed through the Diabetes Treatment Satisfaction Questionnaires. These questionnaires are for the evaluation of thoughts, concerns, and distress related to diabetes and general quality of life assessments. the status version (DTSQs) will be self-applied to the participants in a paper form at visit 2 (for the baseline) and the change version (DTSQc) at visit 4 (last visit). The provided information will be recorded in the corresponding eCRF by the study team.

9.2.3 Assessment of other outcomes of interest

Patient-reported eating behavior and food intake will be assessed with the data from the SNAQ App. Participants will be instructed to document their food intake using the SNAQ App for five days before each on-site study visit, starting during the run-in period (before visit 2), 3, and 4.

9.2.4 Assessment of safety outcomes

Adverse events

All adverse events and serious adverse events (SAEs) that occur during the entire duration of the study (from the time the participant signs the informed consent until the last protocol-specific procedure has been completed, including the safety follow-up period) will be documented and reported according to **Chapter 10**. The time of onset, duration, resolution, action taken, assessment of intensity, relationship to the study treatment and investigational procedures, expectedness, and seriousness will be collected and subsequently entered into the eCRF.

At the beginning of the procedure, the participants will be asked to mention any kind of discomfort. A physician will assess whether the discomfort qualifies as an adverse event. As a physician will be present during the clinical visits, the participant events will be evaluated continuously.

Laboratory parameters

Laboratory tests are detailed in **Annex 6** and should be conducted according to the schedule of activities for each participant (**Table 1**). Any clinically significant findings from laboratory tests that result in a diagnosis and that occur after the patient receives the first dose of the investigational product should be reported by the PI/sponsor as an AE via eCRF. The samples processed and reported will be conducted as described for the secondary (and exploratory) outcomes. **See Subsection 9.4.1** for the definitions of the adverse events and when abnormal laboratory parameters will be considered adverse events.

Vital signs

Vital signs: heartbeat by pulse and blood pressure will be assessed as described for the secondary (and exploratory outcomes). In addition, the temperature will be measured at visits 1 and 2 to rule out infection.

For each participant, a 12-lead ECG will be collected. The electrocardiograms will be recorded after the participant has been supine for five minutes in a quiet room. Electrocardiograms will initially be interpreted by a qualified physician, the investigator, or a qualified designee at the site as soon after ECG collection as possible. Ideally, when the participant is still present for immediate action, should any clinically relevant findings be identified. Any clinically significant findings from ECGs that result in a diagnosis after the participant receives the first dose of the investigational will be reported as an AE via the eCRF. Copies of ECGs collected on thermal paper should source data and be properly stored.

9.2.5 Assessments in participants who prematurely stop the study.

Individual participant-stopping criteria.

Study drug administration may be stopped for any of the following reasons:

- Three or more episodes (separated by at least 120 minutes) of clinically significant hypoglycemia (Level 2) within one day (defined as 00:00 – 23:59) of the study, after excluding other causes such as the omission of meals, unexpected increases in physical activity, or exercise (or any other contributing factor more likely than the IMP) (**See Annex 10**).
- One or more episodes of severe hypoglycemia (Level 3) (**See Annex 10**).
- One or more DKA events unrelated to the participant's AID system malfunction.
- Adjudicated hepatic or pancreatic AEs.
- Incidence of allergic and hypersensitivity reactions.
- If a participant misses three or more consecutive doses of tirzepatide due to intolerable GI symptoms (e.g., nausea, vomiting, or diarrhea) that could be reasonably attributed to the study drug, despite the mitigation plan (**See Annex 8**).
- If a participant is diagnosed with an active or untreated malignancy (other than basal or squamous cell skin cancer, in situ carcinomas of the cervix, in situ, or grade group 1 [that is, Gleason 6 or lower] prostate cancer) after randomization.
- Dehydration or renal acute events.
- Post randomization calcitonin value ≥35 ng/L and increased at least 50% over baseline.
- Supraventricular arrhythmias and cardiac conduction disorders.
- If a female participant becomes pregnant.
- Investigator decides that, in the interest of the patient, it is not medically acceptable to continue participation in the study.
- Participants with a normal BMI at baseline will be closely monitored during clinical visits. The medication will be reduced to 2.5 mg or discontinued if there is a weight gain that exceeds 5% of total body weight or if weight reduction is too rapid (defined as greater than 1% of total body weight per week) following clinical evaluation

Table 1 describes the time points of assessment. For the definition of these AEs/SAEs and for follow-up and procedures for participants who prematurely stop the study, see **Subsection 10.1.2.**

Criteria for suspending or stopping the study.

More than three cases of severe hypoglycemia (level 3, see **Annex 10** for hypoglycemia classification) or more than one case of DKA which were not deemed due to the participant's AID system malfunction (For definitions, **see Subsection 10.1.2**). These criteria are based on exceeding the average incidence of severe hypoglycemia (11.8%) and DKA (4.8%) in patients with T1D, as reported in the Type 1 Diabetes Exchange Clinic Registry (88).

9.3 Procedures at each visit

9.3.1 Visit 1 – Screening.

After informed consent is obtained, the study staff will conduct vital signs and physical evaluation (including height and weight). The following procedures may be performed, data collected, and eligibility criteria checked and documented:

- Inclusion and exclusion criteria assessed.
- Demographics (year of birth, race/ethnicity, and gender).
- Diabetes and medical history (including severe hypoglycemia, DKA history, and microvascular complications).
- Substance use history (drinking, smoking, and drug habits).
- Prior and/or concomitant medications.
- Physical examination including weight, height, waist & hip circumference, waist-to-hip ratio, and vital signs (blood pressure, heart rate, and temperature).
- Electrocardiogram (ECG)
- Serum pregnancy test (serum beta-Hcg) for all females of child-bearing potential.
- Laboratory tests including Hb, hct, total serum bilirubin, direct bilirubin, indirect bilirubin, ALT, AST, ALP, PTT, PT, INR, GGT, serum albumin, pancreatic serum amylase, serum lipase, creatinine, eGFR (calculated by CKD-EPI), urine albumin/creatinine ratio (uCR), calcitonin, TSH, FT4, fasting plasma glucose (FPG) C-peptide (fasting), estradiol and FSH (if apply), TC, LDL, HDL, and triglycerides.
- HbA1c level measured using the DCA Vantage or similar point-of-care device/local lab.
- The study participant will receive a hypoglycemia diary to document clinically significant or severe (see **Annex 10** for hypoglycemia classification) hypoglycemia events, related symptoms, and precipitating activities such as meal omission, increased exercise frequency/intensity, or any other potential causes of hypoglycemia.
- AE/SAE assessment according to **Section. 10.**

Diabetes management information: We will record the participant's average total daily insulin use and total bolus insulin use (calculated over approximately two weeks), carbohydrate ratio, and correction factor (insulin sensitivity factor). Data from the participant's glucometer and CGM may be downloaded or recorded.

The participant will receive a study phone (if needed), an activity tracker (Fitbit sense 2), and the Dexcom G7 CGM system. The study apps (Fitbit app/SNAQ app) will be installed, and participants will receive training on their use. The study handbook also will be given to the participants.

If an exclusionary condition is identified, the study participant will be excluded from participation with follow-up and referral to their primary care physician as needed.

2-Weeks Run-in Phase (baseline collection period)

This phase may begin immediately after enrollment is complete or be deferred for (maximum) 30 days if needed. This observational segment will collect data to compute baseline glycemic control metrics. Dietary history will be collected using the SNAQ app (for five days before the next on-site visit [V2]) to determine daily macronutrient consumption. The baseline data collection period will last for about two weeks up to 21 days after screening. Participants will be under their insulin-related standard of care treatment and wear their personal insulin pump and study-provided CGM. They will be asked to follow their usual care regimen with no restrictions. If needed, one or more interim visits or phone contacts may occur to assist the participant with any issues. Interim visit procedures will include the following:

- Assessment of compliance with the insulin-related standard of care treatment.
- Assessment of skin reaction in areas where a CGM sensor (and infusion set if applicable) was

worn.
- Assessment of eligibility to continue in the study.

To be eligible for randomization, participants must collect in at least 70% of the run-in phase time. If a participant fails to meet this criterion, or if it is determined that the participant will benefit from additional time with equipment use, the run-in period may be extended at the investigator's discretion. Additional visits and phone contacts for further training are at the investigator's discretion.

9.3.2 Visit 2 – Randomization.

Participants will be randomized 1:1 tirzepatide+AID or SoC+AID (**See Figure 1**).

Randomization arm A. Tirzepatide (*Mounjaro*®) +AID

We will dispense the study drug and start the titration phase of tirzepatide (*Mounjaro*®) for the intervention arm. A dose of 2.5 mg SC will be self-administered weekly for four weeks. The study participant will receive instructions on how to use the IMP (in the study handbook adapted for the intervention group) and the medication logbook for compliance check (**See section 8.4**). After four weeks of titration, the study drug will be up-titrated to 5.0 mg SC and kept for 16 weeks.

The study physician will configure the systems and pump parameters to mitigate the risk of hypoglycemia (**See Annex 2**). The study staff will review with the participants any possible parameter adjustments and frequency and how to identify when a parameter is being adjusted.

Randomization arm B. Standard of Care+AID

Participants in arm B will continue with the SoC for diabetes management and AID during the titration and assessment period (16 weeks).

Both randomization arms:

During this visit, the study team will also perform the following evaluations:

- Vital signs (blood pressure, heart rate, and temperature)
- ECG
- Body composition (BIA)
- MAFLD and liver fibrosis assessment (*FibroScan*®)
- Urine pregnancy test
- Device downloads (CGM and pump data)
- Apps Downloads (SNAQ App, activity tracker)
- Questionnaires (DTSQs), the participant will complete a paper questionnaire, and the study staff will enter the data in REDCAp.
- The hypoglycemia diary will be collected and reviewed
- AE/SAE assessment according to **Section. 10**

Additionally, all participants will receive the CGM Dexcom G7 system (re-supply), a Ketone meter, and additional supplies (test strips, lancing devices, and lancets).

9.3.3 Phone Visit 1

Intervention arm:

The study staff will contact the participant to give instructions for escalation to the target dose (5 mg SC weekly). The investigators judge that participants might need more time in the titration phase. This visit can also be used to adjust the insulin pump's parameters if needed (**See Annex 3**).

Both randomization arms:

The study staff ensures that the data from the devices (CGM and pump data) have been downloaded, the hypoglycemia diary reviewed, and performs the AE/SAE assessment according to **Section. 10.**

9.3.4 Visit 3

Both randomization arms:

During this visit, the study team will perform the following evaluations.

- Weight, height, waist & hip circumference, waist-to-hip ratio
- Vital signs (blood pressure and heart rate)
- ECG
- Urine pregnancy test
- Device downloads (CGM and pump data)
- Apps Downloads (SNAQ App, activity tracker)

- AE/SAE assessment according to **Section. 10** (including assessment for clinically significant hypoglycemia, severe hypoglycemia, and DKA)
- The hypoglycemia diary will be collected and reviewed.
- Laboratory tests including total bilirubin, indirect and direct bilirubin, ALT, AST, pancreatic serum amylase, serum lipase, and creatinine with eGFR (calculated by CKD-EPI).

<u>Intervention arm:</u>

The participant's medication logbook will be reviewed, the IMP's used pen will be collected, and the study physician will configure the systems and pump's parameters if needed (**See Annex 3**).

9.3.5 Phone Visit 2

<u>Both randomization arms:</u>

During this visit, the study team will contact the participants by phone. The study staff ensures that the data from the devices (CGM and pump data) have been downloaded, the hypoglycemia diary reviewed, and perform the AE/SAE assessment, according to **Section 10**.

<u>Intervention arm:</u>

If needed, the study physician will configure the systems and pump's parameters (**See Annex 3**).

9.3.6 Visit 4 – End of intervention

<u>Both randomization arms:</u>

During this visit, the study team will perform the following evaluations:

- Weight, height, waist & hip circumference, waist-to-hip ratio
- Vital signs (blood pressure and heart rate)
- ECG
- Body composition (BIA)
- MAFLD and Liver fibrosis assessment (FibroScan®)
- Urine pregnancy test
- Device downloads (CGM and pump data)
- AE/SAE assessment according to **Section. 10** (including assessment for clinically significant hypoglycemia, severe hypoglycemia, and DKA).
- The hypoglycemia diary will be collected and reviewed.
- Questionnaires (DTSQc), the participant will complete a paper questionnaire, and the study staff will enter the data in REDCAp.
- Laboratory tests including total bilirubin, indirect and direct bilirubin, ALT, AST, pancreatic serum amylase, serum lipase, creatinine with eGFR (calculated by CKD-EPI), uACR, calcitonin, HbA1c, CT, LDL, HDL, and triglycerides.

At visit 4, all the study devices will be returned.

<u>Intervention arm:</u>

The participant's medication logbook will be reviewed and collected. As well as the IMP's used pens.

9.3.7 Phone Visit 4 – Follow-up

<u>Intervention arm:</u>

This visit will be conducted about two weeks after the end of the treatment period for follow-up and safety outcomes (AE/SAE assessment), only for the participant in the intervention arm.

9.3.8 Others

Additional visits may be conducted in the event of premature discontinuation of the IMP. The assessments for the premature discontinuation (PD) visit will mirror those of visit 4 (end of intervention) and should be scheduled at the time of IMP discontinuation. A phone follow-up should be conducted two weeks after the PD visit to monitor safety outcomes (AE/SAE assessment). Additionally, at any point in the study, an unscheduled visit may be performed, if needed, at the investigator's discretion (the assessments for this unscheduled visit will be oriented/performed under clinical criteria).

10. SAFETY

10.1 Drug studies

10.1.1 Definition and Assessment of (Serious) Adverse Events and Other Safety-Related Events

An **Adverse Event (AE)** is any unfavorable medical event in a participant in a clinical investigation who has been administered a pharmaceutical product, and it does not necessarily have a causal relationship with the study procedure. An AE can, therefore, be any unfavorable and unintended sign (including an abnormal laboratory finding), symptom, or disease temporally associated with the use of a medicinal (investigational) product, whether related to the medicinal (investigational) product. [ICH E6 1.2]

A **Serious Adverse Event (SAE)** is classified as any untoward medical occurrence that:

- results in death,
- is life-threatening,
- requires in-patient hospitalization or prolongation of existing hospitalization,
- results in persistent or significant disability/incapacity, or
- is a congenital anomaly/birth defect.

In addition, important medical events that may not be immediately life-threatening or result in death or require hospitalization but may jeopardize the participant or may require intervention to prevent one of the other outcomes listed above should also usually be considered serious. [ICH E2A]

SAEs should be followed until resolution or stabilization. Participants with ongoing SAEs at study termination (including safety visit) will be further followed up until recovery or until stabilization of the disease after termination.

Examples of such events are intensive treatment in an emergency room or at home for allergic bronchospasm, blood dyscrasias, or convulsions that do not result in hospitalization or the development of drug dependency or drug abuse.

Adverse Drug Reaction (ADR)

An adverse reaction is an adverse event for which the causal relationship between the study drug and the Adverse Event is suspected.

Serious Adverse Reaction (SAR)

An adverse event that fulfills both the criteria for SAE and ADR.

Medical event of special interest (MESI)

A MESI is an event that, in evaluating safety, has a special focus. A MESI is an AE (SAE or non-serious AE) that fulfills one or more of the below-defined MESI criteria:

Medication errors concerning trial products:

- Administration of the wrong drug
- Wrong route of administration, such as intramuscular instead of subcutaneous
- Accidental administration of a lower or higher dose than intended; however, the administered dose must deviate from the intended dose to an extent where clinical consequences for the trial participant were likely to happen as judged by the investigator, although not necessarily did happen. Overdose and missed insulin bolus resulting in severe hypoglycemia or hyperglycemia are considered AE or SAE, depending on severity.

Unexpected Adverse Drug Reaction

An “unexpected” adverse drug reaction is an adverse reaction, the nature or severity of which is not consistent with the applicable product information [ICH E2A].

Suspected Unexpected Serious Adverse Reactions (SUSARs)

The Sponsor/Investigator evaluates any SAE that has been reported regarding seriousness, causality, and expectedness. If the event is related to the study drug and is both serious and unexpected, it is classified as a SUSAR.

<u>Assessment of Causality</u>

Both Investigator and Sponsor-investigator will make a causality assessment of the event to the study drug based on the criteria listed in the ICH E2A guidelines:

Relationship	Description
Definitely	Temporal relationship<br>Improvement after dechallenge*<br>Recurrence after rechallenging.<br>(or other proof of drug cause)
Probably	Temporal relationship<br>Improvement after dechallenge<br>No other cause evident
Possibly	Temporal relationship<br>Other cause possible
Unlikely	Any assessable reaction that does not fulfill the above conditions
Not related	A causal relationship can be ruled out
*Improvement after dechallenge only taken into consideration, if applicable to the reaction	

Assessment of Severity

The investigator will assess the severity of each AE/SAE reported during the study and assign it to 1 of the following categories:

- Mild: An event that is easily tolerated by the participant, causing minimal discomfort and
- not interfering with everyday activities.
- Moderate: An event that causes sufficient discomfort and interferes with everyday
- activities.
- Severe: An event that prevents everyday activities. An AE assessed as severe should not be confused with an SAE. Severe is a category utilized for rating the intensity of an event, and both AEs and SAEs can be assessed as severe (an event is defined as 'serious' when it meets at least 1 of the predefined outcomes as described in the definition of a SAE, NOT when it is rated as severe.

10.1.2 Reporting of serious adverse events (SAE) and other Safety-Related events

During the entire duration of the study, all AEs and all serious adverse events SAEs will be collected, fully investigated, and documented in source documents and eCRF. Study duration encompasses when the participant signs the informed consent until the last protocol-specific procedure has been completed, including a safety follow-up period.

Reportable AEs for Hypoglycemia and Hyperglycemia/Diabetic Ketoacidosis.

Hypoglycemia: Hypoglycemia is common in people with T1D. Clinically significant hypoglycemia (Level 2), defined as a blood glucose concentration of <3.0 mmol/L (<54 mg/dL) for at least 15 consecutive minutes in the CGM, typically leading to neuroglycopenic symptoms, will be reported as an AE if other causes, such as meal omission, unexpected increases in physical activity, or exercise, miscalculations of boluses by the participant or any other contributing factors, have been excluded. A diary for hypoglycemia events will be provided to participants for recording BG levels measured before treatment administration if taken, associated symptoms, treatment administered, and information about contributing factors, if applicable, as described previously.

Only severe hypoglycemia (or hypoglycemia level 3), defined as "a hypoglycemia event requiring medical assistance of another person due to altered consciousness and requiring another person to actively administer carbohydrate, glucagon, or other resuscitative actions" is reportable as an SAE. This means that the participant was impaired cognitively to the point that he/she was unable to treat himself/herself, was unable to verbalize his/ her needs, was incoherent, disoriented, and/or combative, or experienced seizure or coma. These episodes may be associated with sufficient neuroglycopenia to induce seizure or coma. If plasma glucose measurements are unavailable during such an event, neurological recovery attributable to restoring plasma glucose to normal is considered sufficient evidence that a low plasma glucose concentration induced the event.

Hyperglycemic events/Diabetic Ketoacidosis (DKA): Hyperglycemic event is only reportable as an adverse event when one of the following four criteria is met:

1) The event involved DKA, as defined by the Diabetes Control and Complication Trial (DCCT) and described here:

   Hyperglycemic events are classified as DKA if the following are present (meeting all 4 criteria):

   a) Symptoms such as polyuria, polydipsia, nausea, or vomiting
   b) Serum ketones >1.5 mmol/L or large/moderate urine ketones
   c) Either arterial pH <7.30 or venous pH <7.24 or serum bicarbonate <15
   d) Treatment provided in a healthcare facility.
2) Evaluation of treatment was obtained at a healthcare provider facility for an acute event involving hyperglycemia or ketosis.
3) Blood ketone level ≥ 1.5 mmol/L and communication occurred with a health care provider during the event.
4) Blood ketone level ≥ 2.5 mmol/L (even) without communication with a health care provider.

Adjudicated hepatic or pancreatic AEs (Pancreatitis)

The participants with a history of pancreatitis for any cause and risk factor for pancreatitis (e.g., alcoholism, triglycerides (≥5.7 mmol/L [≥500 mg/dL]) will be excluded from this trial. However, acute pancreatitis is defined as an AE of interest in all trials with tirzepatide, including this trial. Acute pancreatitis is an acute inflammatory process of the pancreas that may also involve peripancreatic tissues and/or remote organ systems(104). The diagnosis of acute pancreatitis requires two of the following three features:

1) abdominal pain, characteristic of acute pancreatitis (generally located in the epigastrium and radiates to the back in approximately half the cases; the pain is often associated with nausea and vomiting).
2) serum amylase (total and/or pancreatic) and/or lipase ≥3X ULN.
3) characteristic findings of acute pancreatitis on computed tomography (CT) scan or magnetic resonance imaging (MRI).

If acute pancreatitis is suspected, appropriate laboratory tests (including levels of pancreatic amylase [p-amylase] and lipase) should be obtained via the central laboratory (and locally, if needed). Imaging studies, such as abdominal CT scans with or without contrast, MRI, or gallbladder ultrasound, should be performed. If laboratory values and/or abdominal imaging support the diagnosis of acute pancreatitis, the patient must discontinue therapy with the study drug. The patient's concomitant medications should be reviewed to assess any other potential causal.

Each case of an AE of pancreatitis will be reported. If typical signs and/or symptoms of pancreatitis are present and confirmed by laboratory values (lipase or amylase [total and/or pancreatic]) and imaging studies, the event must be reported as an SAE. For a potential case that does not meet all these criteria, it is up to the investigator to determine the seriousness of the case (AE or SAE) and the relatedness of the event to the study drug.

Adjudicated hepatic or pancreatic AEs (Hepato-biliary disorders)

Hepato-biliary events will be considered AEs. All events of treatment, emergent biliary colic, cholecystitis, or other suspected events related to gallbladder disease will be evaluated, and additional diagnostic tests will be performed as needed. If a study participant experiences elevated ALT ≥3X ULN, ALP ≥2X ULN, or elevated TBL ≥2XULN, liver testing (**See Annex 9**) will be repeated within three to five days, including ALT, AST, ALP, TBL, direct bilirubin, and GGT to confirm the abnormality and to determine if it is increasing or decreasing. If the abnormality persists or worsens, clinical and laboratory monitoring will be initiated by the investigator and in consultation with the study medical monitor. ALT, AST, TBL, and ALP monitoring should continue until levels normalize or return to approximate baseline levels.

Additional safety data will be collected via the eCRF, and the IMP should be stopped if one or more of the following conditions occur:

- Elevation of serum ALT or AST >8 times ULN
- Elevation ALT or AST >5 times ULN for more than 2 weeks
- Elevation ALT or AST >3 times ULN and TBL >2 times ULN or INR >1.5
- Elevation ALT or AST >3 times ULN with the appearance of fatigue, nausea, vomiting, right upper-quadrant pain or tenderness, fever, rash, and/or eosinophilia (>5%)
- Elevation ALP >3 times ULN
- Elevation ALP >2.5 times ULN and TBL >2 times ULN
- Elevation ALP >2.5 times ULN with the appearance of fatigue, nausea, vomiting, right quadrant pain or tenderness, fever, rash, and/or eosinophilia (>5%)
- Elevated serum TBL to ≥ 2X ULN (except for cases of known Gilbert's syndrome).

Dehydration and Acute Renal Events

Renal safety will be assessed based on repeated renal functional assessment and assessment of AEs suggestive of acute or worsening chronic renal failure. Gastrointestinal AEs, including nausea, diarrhea, and vomiting, have been reported with tirzepatide. These are consistent with other GLP-1 receptor

agonists. The events may lead to dehydration, which could cause a deterioration in renal function, including acute renal failure. Patients will be advised to notify investigators in case of severe nausea, frequent vomiting, or symptoms of dehydration.

Severe gastrointestinal adverse events

Tirzepatide may cause severe GI AEs, such as nausea, vomiting, and diarrhea. The eCRF will collect information about any intolerance or persistent GI AEs and antiemetic/antidiarrheal use. For detailed information concerning the management of GI AEs, please **see Annex 8**.

Thyroid Malignancies and C-cell hyperplasia

Individuals with a personal or family history of MTC and/or MEN-2 or screening calcitonin >35ng/L will be excluded from the study. The assessment of thyroid safety during the study will include reporting any case of thyroid malignancy, including MTC and papillary carcinoma, and calcitonin measurements. This data will be captured in specific eCRFs. The purpose of calcitonin measurements is to assess the potential of tirzepatide to affect thyroid C-cell function, which may indicate the development of C-cell hyperplasia and neoplasms. Tirzepatide should be discontinued (after first confirming the value) if the post-randomization calcitonin value is ≥35 ng/L and has increased at least 50% over baseline. We will evaluate the concomitant medication for other potential causes. If the increased calcitonin value (≥35 ng/L and increases by ≥50% compared with baseline) is observed in a patient who has administered a medication that is known to increase serum calcitonin, this medication should be stopped, and calcitonin levels should be measured after an appropriate washout period. If the confirmed calcitonin value is <35 ng/L, tirzepatide should be restarted when safe (assessed by the study PI).

Hypersensitivity Events

The investigator will report all allergic or hypersensitivity reactions as either AEs or SAEs if any serious criterion is met. Additional data, such as the type of reaction and treatment received, will be collected on any AEs or SAEs that the investigator deems related to the study drug via an eCRF created for this purpose. The study drug should be temporarily interrupted in any individual suspected of having a severe allergic reaction to the study drug. At the investigator's discretion, the study drug may be restarted when/if deemed safe. If the study drug is permanently discontinued, the patient will continue in the trial to collect all planned efficacy and safety measurements.

Injection Site Reactions

Injection site reactions will be collected on the eCRF separate from the hypersensitivity reaction eCRF.

Supraventricular Arrhythmias and Cardiac Conduction Disorders

Treatment-emergent cardiac conduction disorders will be further evaluated. Additional diagnostic tests to determine the exact diagnosis will be performed as needed. The specific diagnosis will be recorded as an AE. Events that meet the criteria for serious conditions, as described in **Section 10.1.1**, will be reported as SAEs.

10.1.2.1 Foreseeable adverse events

Hypoglycemia or hyperglycemia events not meeting the above criteria are not required to be reported as an adverse event.

Participant’s own devices (e.g. insulin pump, CGM, BG meters) related issues and skin issues arising from using these devices (such as skin rash due to adhesive or infusion site issues) are not reportable unless the event meets the definition of an SAE.

10.1.2.2 Reporting of SAEs

All SAEs will be reported to the study Sponsor immediately and within a maximum of 24 hours. The Sponsor-Investigator will re-evaluate the SAE in REDCap. SAEs resulting in death will be reported to the Ethics Committee via BASEC within seven days.

10.1.2.3 Reporting of SUSARs

A SUSAR needs will be reported to the Ethics Committee (local event via local Investigator) via BASEC and Swissmedic via the Sponsor-Investigator within seven days if the event is fatal or within 15 days (all other events).

10.1.2.4 Reporting of immediate safety and protective measures

All suspected new risks and relevant new aspects of known adverse reactions that require immediate safety-related measures will be reported to the Sponsor-Investigator within 24 hours. The Sponsor/Investigator will report these measures within seven days to the Ethics Committee (local event

via local Investigator) via BASEC and Swissmedic.

10.1.2.5. Reporting and Handling of Pregnancies

Pregnant participants will be immediately withdrawn from the clinical study. Any pregnancy during the treatment phase of the study and within 30 days after discontinuation of study medication will be reported to the Sponsor/Investigator within 24 hours. The course and outcome of the pregnancy will be followed up carefully, and any abnormal outcome regarding the mother or the child will be documented and reported.

10.1.2.6 Periodic reporting of safety

The ASR is written in compliance with ICH Harmonised Guideline E2F for a non-commercial sponsor.

An ASR is submitted once a year to the Ethics Committee and to Swissmedic via the Sponsor-Investigator.

The start date for the ASR is the date of the sponsor's first authorization to conduct a clinical trial

The ASR is submitted to the Ethics Committee and Swissmedic throughout the clinical study in Switzerland, and the last ASR submission will cover the last patient-last visit.

10.1.3 Follow-up of (Serious) Adverse Events

Follow-up of adverse events: The investigator will provide adequate medical care to the study participants for any study-related adverse events, including clinically significant laboratory values related to the study. Medical care should be provided to the participants regardless of their insurance status. AE classified as serious or possibly/probably related to the study drug will be followed until the participant has recovered and all queries have been resolved or until the end of the post-treatment follow-up stated in the protocol, whichever comes first.

The medical assessment, vital signs, and laboratory measurements are documented and reported as applicable to the specific event. Participants will be called in case otherwise cannot be reached.

10.2 Assessment, notification, and reporting on the use of radiation sources

Not applicable

11. STATISTICAL METHODS

11.1 Hypothesis

The primary outcome of this study is the Mean difference in 24/7 CGM percentage time spent between 3.9 and 10 mmol/L (TIR) between groups at week 16 of treatment (**Figure 1**). The difference in TIR will be established in a parallel design using a statistical significance of α=0.05.

The null/alternative hypotheses are:

- Null hypothesis: There is no mean 24/7 CGM-based TIR difference between tirzepatide+AID (intervention) vs. SoC+AID (control) at 16 weeks of treatment.

- Alternative hypothesis: The mean for 24/7 CGM-based TIR differs for tirzepatide+AID (intervention) and SoC+AID (control) at 16 weeks of treatment.

11.2 Determination of Sample Size

Assuming a 1:1 randomization ratio, we calculated that a sample size of 42 participants (21 in each arm) would provide 80% power with a type I error rate (two-sided) of 5% to reject the null hypothesis with the following assumption: a standard deviation (SD) at baseline for TIR of 15%, a pre-post difference of TIR in the control group of 0.2%, a pre-post difference of TIR in the treatment group of 10.5%, a correlation of 0.7, and an attrition rate of 20%. The assumptions were taken from retrospective studies in T1D using tirzepatide (34,35).

11.3 Statistical criteria for termination of trial

As described in **Subsection 9.2.5**.

11.4 Planned Analyses

This study will assess the safety and efficacy of tirzepatide (*Mounjaro®*) added to existing AID systems through a change in TIR and secondary and exploratory endpoints, including other CGM-based metrics, HbA1c, and surrogate endpoints for CVD, MAFLD, and renal markers. All variables will be tested for normality with the Shapiro-Wilk and/or the Kolmogorov-Smirnov tests. Continuous and normally distributed data will be presented as mean±SD and categorical as N/%. Non-normally distributed variables will be analyzed using non-parametric tests and presented as median [IQR]. Stata software version 18 and IBM SPSS statistics version 30.0.0 will be used for the statistical analyses.

11.4.1 Datasets to be analyzed, analysis populations

We will prepare two data sets for the analysis:

- The per-protocol (PP) analysis set: includes only participants who complete the treatment according to the protocol (from the end of titration until 16 weeks of study).
- Intention-to-treat (ITT) analysis set: includes all randomized participants as originally allocated after randomization. ITT data set will be used for safety and efficacy analyses.

Due to the small sample size, no subgroup analysis is planned.

11.4.2 Primary Analysis

The study design allows repeated measures (before and after) of the primary outcome and exploratory outcomes. We will use repeated measures analysis of covariance (ANCOVA) for the primary outcome while adjusting for HbA1c, BMI, and baseline TIR. A point estimate 95% confidence interval and two-sided p-value will be reported for the interventions (tirzepatide+AID vs SoC+AID), and a 5% level will be used to declare statistical significance. Residual values will be examined for an approximate normal distribution.

11.4.3 Secondary Analyses

The secondary outcomes will be assessed using a mixed-effect linear model for repeated measures (MMRM) from baseline through 8, 12, and 16 weeks of treatment, respectively, with HbA1c and BMI as fixed effects, and baseline TIR as a covariate. A point estimate 95% confidence interval and two-sided p-value will be reported for the intervention (tirzepatide+AID vs. SoC+AID), and a 5% level will be used to declare statistical significance. Residual values will be examined for an approximate normal distribution.

11.4.4 Interim analyses

As a clinical study for 16 weeks, no interim analysis is planned.

11.4.5 Safety analysis

The entire ITT population will be included for safety analysis (such as AE/SAE). A table will be populated with the frequency of system-wide adverse events between two groups. Differences in safety outcomes will be examined between ITT groups using Chi-Square tests for categorical outcomes (any AE/SAE, clinically significance hypoglycemia, severe hypoglycemia, DKA) and non-parametric tests for continuous variables with non-normal distribution such as time spent in hypoglycemia on CGM.

11.4.6 Deviation(s) from the original statistical plan

Any deviation(s) from the original statistical plan will be described and justified in the final report as appropriate.

11.5 Handling of missing data and drop-outs

No imputation methods for missing data will be used. The drop-outs will be replaced by new participants until 42 participants (21 in each arm) have been randomized.

12. QUALITY ASSURANCE AND CONTROL

12.1 Data handling and record keeping/archiving

12.1.1 Case Report Forms

The Sponsor and investigator will maintain appropriate medical and research records for this study in compliance with ICH-GCP and regulatory and institutional requirements to protect the confidentiality of participants. The investigators and study team members will have access to the records.

The eCRFs will be done using REDCap electronic data capture tools hosted at the Diabetes Center Berne (DCB) cloud servers. All data requested on the eCRF must be recorded and should be consistent with the source documents. Any discrepancies should be explained. The Investigator should ensure the accuracy, completeness, and timeliness of the data recorded in the eCRF and all other required reports. Available data should be entered into the eCRF within two weeks.

All study team members are authorized to enter data into the eCRF. The Sponsor is responsible for properly training and instructing the study team members to enter data into the eCRF. All study-related data will be collected in a coded manner by assigning each participant a study ID (unique, consecutive numbered).

A source document is where the collected data is originally recorded. Source documents in this study entail the following:

- Reports of diagnostic test results
- Signed and dated informed consent forms.
- Electronic food intake (SNAQ App downloads) and symptoms diary.
- CGM downloads (Dexcom G7 downloads)
- Insulin pump downloads
- Activity tracker (Fitbit sense 2 downloads)
- Medical reports, including medication logs.
- eCRFs on which data are entered directly rather than extracted from another source document.

Any change or correction to source data should be dated, marked with staff initials, and explained (if necessary) and not obscure the original entry. All data captured in the eCRF will be itemized on a source data location list, which will be stored in the investigator site file. This list should indicate the source data location corresponding to each eCRF entry. The priority order must be specified in the list if several sources are available for one eCRF entry. If certain data are directly entered into the eCRF (and are thus considered source data), this must be specified on the source data location list accordingly.

If a participant withdraws his/her consent from the study, their data will be kept coded until the analysis is complete. After the analysis, they will be blacked out on the identification log. **See Section 2.8** for participant privacy and confidentiality.

12.1.2 Specification of source documents

Source data are all information in original records and certificated copies of original records of clinical findings, observations, or other activities in a clinical investigation necessary for the reconstruction and evaluation of the investigation. This includes study-relevant participant data, data captured from the eCRFs, and data downloaded from the AID systems and CGM devices. The source data and source documents for each key data point during the study will be captured in a source data identification log.

12.1.3 Record keeping/archiving

The data and specimens obtained from the participants will be identified with a unique number (identifier). The sponsor will retain all data generated during the study. Data management is the responsibility of the sponsor and investigator. All electronic data will be stored at the Sponsor's secure cloud in a de-identified manner. The data will be accessible only by the study team, and if data transfer is needed, appropriate measures, including encryption of data files, will be used to ensure security and participant confidentiality.

After the study's completion, all the study documents and data will be stored as required by applicable national and international legislation. The records will be stored securely and kept for a minimum of 10 years after a regular or premature termination of the investigation.

12.2 Data management

Data Management System

REDCap will be used as our clinical data management system (CDMS). Data will only be accessible to authorized personnel who require it to fulfill their duties within the scope of the research project. In the eCRFs and other project-specific documents, participants are only identified by study identifier. Any identifying information will be kept locked and only accessible to dedicated study team members at the clinic. The investigators will maintain appropriate medical and research records for this trial, in compliance with the International Conference on Harmonisation and Good Clinical Practice (ICH-GCP) and regulatory and institutional requirements for the protection of the confidentiality of participants.

12.2.1 Data security, access, and back-up

Data is protected from unauthorized or accidental disclosure, alteration, deletion, copying, and theft. Traceability is guaranteed with the use of REDCap and proper documentation on paper. A role concept with personal passwords (site investigator, statistician, monitor, administrator, etc.). All data entered the eCRFs are transferred to the database using Transport Layer Security (TLS) encryption. Each data point has attributes attached to it, identifying the user who entered it with the exact time and date. Retrospective alterations of data in the database are recorded in an audit table. The time, table, data field, and altered value, ante the person are all recorded (audit trail). A multi-level backup system is implemented. Back-ups of the whole system, including the database, are run internally several times per day and on external tapes once a day.

12.2.2 Analysis and archiving

Health data collected during the study will be downloaded from the respective device and stored in the dedicated database for 10 years. From there, the data will be downloaded and stored locally for analysis and will be removed afterward.

12.2.3 Electronic and central data validation

The electronic data questionnaires mostly contain multiple-choice questions, which make data validation easier. Data entry fields will be controlled to allow only adequate formats when possible.

12.3 Monitoring

During the clinical study, monitoring visits will be conducted by authorized, qualified representatives of the Sponsor. The monitors will review all aspects of the study to ensure that the protocol and applicable regulatory requirements are adhered to and to assure participants' safety. Monitoring activities, as described in a study-specific monitoring plan, will include checking eCRFs for completeness and plausibility and verifying data against source documentation, reviewing the informed consent forms, checking the device accountability log, and ensuring that the investigator site file up to date and contains all required documentation. All source data must be accessible to the monitoring personnel. Monitors must maintain patient confidentiality.

12.4 Audits and Inspections

Study sites, competent authorities, and ethics committees have the right to perform inspections, and the sponsor has the right to perform on-site auditing during working hours upon reasonable prior notice. The investigation documentation and source data/documents must be accessible to auditors/inspectors. Auditors/inspectors will maintain patient confidentiality.

12.5 Confidentiality, Data Protection

All data obtained in the context of the clinical study are subject to data protection. The PI and his/her team, as well as the Sponsor, must assure the participants' privacy and protect their identities from unauthorized parties. On the eCRFs or other documents submitted to the Sponsor, participants must not be identified by their names but by a unique study identifier (pseudonymisation). The PI will maintain any study documents with subject names, e.g., subjects' written consent forms, in strict confidence and as part of the Investigator's Site File. Direct access to source documents will be permitted for the purpose of monitoring (**Section 12.3**), audits, and inspections (**Section 12.4**). Only the study team will have access to the source data during the study.

The study data will be stored in a coded manner in Switzerland. In the frame of the study, data may be shared in a coded manner with a designated collaborator at the University of Antioquia in Medellín, Colombia, for statistical analysis purposes. Access to the coded data will be granted only to the

designated collaborating researcher. The data will be transferred using a secure file-sharing platform that complies with Swiss and international data protection standards. This platform ensures end-to-end encryption to safeguard the data during transfer. The collaborating researcher is responsible for ensuring the secure storage and access of data on their end and must also use encrypted devices or systems approved by his institution. The collaborating researcher will undergo data protection training as per Swiss regulations. Participants are informed of the possible data transfer and agree to it by consenting to participate in the study. The sponsor is responsible for ensuring that the same standards are maintained abroad as in Switzerland.

12.6 Storage of biological material and related health data

Paper-based trial documents (e.g., informed consent) will be adequately stored for at least ten years after completion of the study. The study database with all archive tables will be securely stored at the study research center, and the retention duration of study documents will follow local legal requirements. Biological samples will be analyzed for study outcomes but not stored.

13. PUBLICATION AND DISSEMINATION POLICY

The study results will be submitted for publication in internationally peer-reviewed scientific journals; members of the study team and collaborators will all be co-authors. The privacy and confidentiality of each participant shall be preserved in reports and data publication. Once results have been published, trial data will be accessible to external researchers. Investigators wishing to replicate the analyses or to do an individual patient meta-analysis may request the data from the Sponsor/Investigator.

14. FUNDING AND SUPPORT

14.1 Funding

This study will be fully financed by the Sponsor.

14.2 Other Support

Not applicable.

15. INSURANCE

The insurance is provided by the Sponsor and fulfills the legal provision of Section 4 (Art 13. ClinO). For this purpose, the Sponsor has taken out a special insurance for clinical trials; Chubb Insurance (Switzerland) Ltd, Bärengasse 32, 8001 Zurich. A copy of the certificate is filed in each investigator site file and the trial master file.

16. REFERENCES

1. Diabetes International Federation. IDF [Internet. 2021 [cited 2024 Apr 11]. Type 1 diabetes estimates in children and adolescents. Available from: https://diabetesatlas.org/data/en/indicators/12/
2. E. J. Schoenle, M. Lang-Muritano, S. Gschwend, J. Laimbacher, P.E. Mullis, T. Torresani, et al. Epidemiology of Type I diabetes mellitus in Switzerland: steep rise in incidence in under 5-year-old children in the past decade. Diabetologia. 2001 Mar;44(3):286-9.
3. Mobasseri M, Shirmohammadi M, Amiri T, Vahed N, Fard HH, Ghojazadeh M. Prevalence and incidence of type 1 diabetes in the world: A systematic review and meta-analysis. Health Promot Perspect. 2020 Mar 30;10(2):98-115.
4. Writing Team for the Diabetes Control and Complications Trial/Epidemiology of Diabetes Interventions and Complications Research Group. Sustained effect of intensive treatment of type 1 diabetes mellitus on development and progression of diabetic nephropathy the epidemiology of diabetes interventions and complications (EDIC) study. JAMA. 2003 Oct 22;290(16):2159-67
5. The Diabetes Control and Complications Trial Research Group. The effect of intensive treatment of diabetes on the development and progression of long-term complications in insulin-dependent diabetes mellitus. N Engl J Med. 1993 Sep 30;329(14):977-86.
6. Purnell JQ, John EH, Cleary PA, Nathan DM, Lachin JM, Zinman B, et al. The effect of excess weight gain with intensive diabetes mellitus treatment on cardiovascular disease risk factors and atherosclerosis in type 1 diabetes mellitus: Results from the Diabetes Control and Complications Trial/Epidemiology of Diabetes Interventions and Complications Study (DCCT/EDIC) study. Circulation. 2013 Jan 15;127(2):180–7.
7. Beck RW, Bergenstal RM, Riddlesworth TD, Kollman C. The association of biochemical hypoglycemia with the subsequent risk of a severe hypoglycemic event: analysis of the DCCT data set. Diabetes Technol Ther. 2019 Jan 1;21(1):1–5.
8. Ordinance on Clinical Trials with the exception of Clinical Trials of Medical Devices [Internet]. [cited 2024 Apr 11]. Available from: https://www.fedlex.admin.ch/eli/cc/2013/643/en
9. Eli Lilly (Suisse) SA. Mounjaro ® Fertigpen [Internet]. [cited 2024 Jun 28]. Available from: https://www.swissmedicinfo.ch/ViewMonographie
10. World Medical Association. WMA Declaration of Helsinki – ethical principles for medical research involving human subjects [Internet]. 1964 [cited 2023 Oct 2]. Available from: https://www.wma.net/policies-post/wma-declaration-of-helsinki-ethical-principles-for-medical-research-involving-human-subjects/
11. International Conference on Harmonization (ICH) E6(R2) Guideline for Good Clinical Practice [Internet]. 2016 [cited 2023 Oct 2]. International Conference on Harmonization (ICH) E6(R2) Guideline for Good Clinical Practice. Available from: oducts/Guidelines/Efficacy/E6/E6_R2__Step_4_2016_1109.pdf
12. Schweizerische Ethikkommissionen für die Forschung am Menschen. Leitfaden Bedenkfrist [Internet]. [cited 2024 Apr 11]. Available from: https://swissethics.ch/assets/pos_papiere_leitfaden/bedenkzeit_final_d.pdf
13. Harris PA, Taylor R, Thielke R, Payne J, Gonzalez N, Conde JG. Research electronic data capture (REDCap)-A metadata-driven methodology and workflow process for providing translational research informatics support. J Biomed Inform. 2009 Apr;42(2):377–81.
14. Holt RIG, DeVries JH, Hess-Fischl A, Hirsch IB, Kirkman MS, Klupa T, et al. The management of type 1 diabetes in adults. A consensus report by the American Diabetes Association (ADA) and the European Association for the Study of Diabetes (EASD). Diabetologia. 2021 Dec 1;64(12):2609–52.
15. Wolfsdorf JI, Ratner RE. SGLT inhibitors for type 1 diabetes: Proceed with extreme caution. Diabetes Care. 2019 Jun;42(6):991-993.
16. Schimke KE, Renström F, Meier S, Stettler C, Brändle M. Compliance with guidelines for disease management in diabetes: Results from the SwissDiab Registry. BMJ Open Diabetes Res Care. 2018 Feb 1;6(1).
17. Mathieu C, Gillard P, Benhalima K. Insulin analogs in type 1 diabetes mellitus: Getting better all the time. Nat Rev Endocrinol. 2017 Jul;13(7):385-399.

18. Ray PP. Continuous glucose monitoring: a systematic review of sensor systems and prospects. Sensor Review; 2018 (38). p. 420–37.

19. Nwokolo M, Hovorka R. The Artificial Pancreas and Type 1 Diabetes. J Clin Endocrinol Metab. 2023 Jun 16;108(7):1614-1623.

20. Boughton CK, Hovorka R. Is an artificial pancreas (closed-loop system) for Type 1 diabetes effective? Diabet Med. 2019 Mar;36(3):279-286.

21. Lind M, Svensson AM, Kosiborod M, Gudbjörnsdottir S, Pivodic A, Wedel H, et al. Glycemic Control and Excess Mortality in Type 1 Diabetes. N Engl J Med. 2015 Feb 26;372(9):880-1.

22. Dubose SN, Hermann JM, Tamborlane W V., Beck RW, Dost A, Dimeglio LA, et al. Obesity in Youth with Type 1 Diabetes in Germany, Austria, and the United States. J Pediatr. 2015 Sep;167(3):627-32.e1-4.

23. Haas J, Andersson Franko M, Lindholm Olinder A, Nyström T, Persson M. Time-trends in body mass index, and overweight and obesity as independent risk factors for diabetes angiopathy in young females with type 1 diabetes – A nationwide study in Sweden. Diabetes Res Clin Pract. 2023 Oct 1;204.

24. Bielka W, Przezak A, Molęda P, Pius-Sadowska E, Machaliński B. Double diabetes—when type 1 diabetes meets type 2 diabetes: definition, pathogenesis and recognition. Cardiovasc Diabetol. 2024 Feb 10;23(1):62.

25. Livingstone SJ, Levin D, Looker HC, Lindsay RS, Wild SH, Joss N, et al. Estimated life expectancy in a scottish cohort with type 1 diabetes, 2008-2010. JAMA. 2015 Jan 6;313(1):37–44.

26. De Ferranti SD, De Boer IH, Fonseca V, Fox CS, Golden SH, Lavie CJ, et al. Type 1 diabetes mellitus and cardiovascular disease: A scientific statement from the American Heart Association and American Diabetes Association. Circulation. 2014 Sep 23;130(13):1110-30.

27. Secrest AM, Becker DJ, Kelsey SF, LaPorte RE, Orchard TJ. All-cause mortality trends in a large population-based cohort with long-standing childhood-onset type 1 diabetes: The Allegheny County type 1 diabetes registry. Diabetes Care. 2010 Dec;33(12):2573–9.

28. Jørgensen ME, Almdal TP, Carstensen B. Time trends in mortality rates in type 1 diabetes from 2002 to 2011. Diabetologia. 2013 Nov;56(11):2401–4.

29. Kanter JE, Shao B, Kramer F, Barnhart S, Shimizu-Albergine M, Vaisar T, et al. Increased apolipoprotein C3 drives cardiovascular risk in type 1 diabetes. J Clin Invest. 2019 Jul 11;129(10):4165-4179.

30. Park J, Ntelis S, Yunasan E, Downton KD, Yip TCF, Munir KM, et al. Glucagon-Like Peptide 1 Analogues as Adjunctive Therapy for Patients With Type 1 Diabetes: An Updated Systematic Review and Meta-analysis. J Clin Endocrinol Metab. 2023 Dec 21;109(1):279-292.

31. Underland LJ, Ilkowitz JT, Katikaneni R, Dowd A, Heptulla RA. Use of Sitagliptin with Closed-Loop Technology to Decrease Postprandial Blood Glucose in Type 1 Diabetes. J Diabetes Sci Technol. 2017 May 1;11(3):602–10.

32. Haidar A, Tsoukas MA, Bernier-Twardy S, Yale JF, Rutkowski J, Bossy A, et al. A novel dual-hormone insulin- and-pramlintide artificial pancreas for type 1 diabetes: A randomized controlled crossover trial. Diabetes Care. 2020 Mar 1;43(3):597–606.

33. Maffei P, Bettini S, Busetto L, Dassie F. SGLT2 Inhibitors in the Management of Type 1 Diabetes (T1D): An Update on Current Evidence and Recommendations. Diabetes Metab Syndr Obes. 2023 Nov 9:16:3579-3598.

34. Akturk HK, Dong F, Snell-Bergeon JK, Karakus KE, Shah VN. Efficacy and Safety of Tirzepatide in Adults with Type 1 Diabetes: A Proof of Concept Observational Study. J Diabetes Sci Technol. 2024 Feb 5:19322968231223991.

35. Garg SK, Akturk HK, Kaur G, Beatson C, Snell-Bergeon J. Efficacy and Safety of Tirzepatide in Overweight and Obese Adult Patients with Type 1 Diabetes. Diabetes Technol Ther. 2024 Jun;26(6):367-374

36. Drucker DJ. Mechanisms of Action and Therapeutic Application of Glucagon-like Peptide-1. Cell Metab. 2018 Apr 3;27(4):740-756.

37. Maselli DB, Camilleri M. Effects of GLP-1 and Its Analogs on Gastric Physiology in Diabetes Mellitus and Obesity. In: Advances in Experimental Medicine and Biology. Adv Exp Med Biol. 2021:1307:171-192

38. Tschöp M, Nogueiras R, Ahrén B. Gut hormone-based pharmacology: novel formulations and future possibilities for metabolic disease therapy. Diabetologia. 2023 Oct;66(10):1796-1808.
39. Kanoski SE, Hayes MR, Skibicka KP. GLP-1 and weight loss: unraveling the diverse neural circuitry. Am J Physiol Regul Integr Comp Physiol. 2016;310:885–95.
40. Eng C, Kramer CK, Zinman B, Retnakaran R. Glucagon-like peptide-1 receptor agonist and basal insulin combination treatment for the management of type 2 diabetes: A systematic review and meta-analysis. The Lancet. 2014 Dec 27;384(9961):2228–34.
41. Nauck MA, Mirna AEA, Quast DR. Meta-analysis of head-to-head clinical trials comparing incretin-based glucose-lowering medications and basal insulin: An update including recently developed glucagon-like peptide-1 (GLP-1) receptor agonists and the glucose-dependent insulinotropic polypeptide/GLP-1 receptor co-agonist tirzepatide. Diabetes Obes Metab. 2023 May 1;25(5):1361–71.
42. Mathieu C, Zinman B, Hemmingsson JU, Woo V, Colman P, Christiansen E, et al. Efficacy and safety of liraglutide added to insulin treatment in type 1 diabetes: The adjunct one treat-to-target randomized trial. Diabetes Care. 2016 Oct 1;39(10):1702–10.
43. Johansen NJ, Dejgaard TF, Lund A, Schlüntz C, Frandsen CS, Forman JL, et al. Efficacy and safety of meal-time administration of short-acting exenatide for glycaemic control in type 1 diabetes (MAG1C): a randomised, double-blind, placebo-controlled trial. Lancet Diabetes Endocrinol. 2020 Apr 1;8(4):313–24.
44. Ghanim H, Batra M, Green K, Abuaysheh S, Hejna J, Makdissi A, et al. Liraglutide treatment in overweight and obese patients with type 1 diabetes: A 26-week randomized controlled trial; mechanisms of weight loss. Diabetes Obes Metab. 2020 Oct 1;22(10):1742–52.
45. Wang W, Liu H, Xiao S, Liu S, Li X, Yu P. Effects of Insulin Plus Glucagon-Like Peptide-1 Receptor Agonists (GLP-1RAs) in Treating Type 1 Diabetes Mellitus: A Systematic Review and Meta-Analysis. Diabetes Ther. 2017 Aug;8(4):727-738.
46. Dejgaard TF, Schmidt S, Frandsen CS, Vistisen D, Madsbad S, Andersen HU, et al. Liraglutide reduces hyperglycemia and body weight in overweight, dysregulated insulin-pump-treated patients with type 1 diabetes: The Lira Pump trial—a randomized, double-blinded, placebo-controlled trial. Diabetes Obes Metab. 2020 Apr 1;22(4):492–500.
47. Sherr JL, Patel NS, Michaud CI, Palau-Collazo MM, Van Name MA, Tamborlane W V., et al. Mitigating meal-related glycemic excursions in an insulin-sparing manner during closed-loop insulin delivery: The beneficial effects of adjunctive pramlintide and liraglutide. Diabetes Care. 2016 Jul;39(7):1127-34.
48. Renukuntla VS, Ramchandani N, Trast J, Cantwell M, Heptulla RA. Role of Glucagon-like peptide-1 analogue versus Amylin as an adjuvant therapy in type 1 diabetes in a closed loop setting with ePID algorithm. J Diabetes Sci Technol. 2014 Jan 1;8(5):1011–7.
49. Karakus KE, Klein MP, Akturk HK, Shah VN. Changes in Basal and Bolus Insulin Requirements with Tirzepatide as an Adjunctive Therapy in Adults with Type 1 Diabetes Using Tandem Control-IQ. Diabetes Ther. 2024 Jul;15(7):1647-1655.
50. Sattar N, Lee MMY, Kristensen SL, Branch KRH, Del Prato S, Khurmi NS, et al. Cardiovascular, mortality, and kidney outcomes with GLP-1 receptor agonists in patients with type 2 diabetes: a systematic review and meta-analysis of randomised trials. Lancet Diabetes Endocrinol. 2021 Oct 1;9(10):653–62.
51. Huxley RR, Peters SAE, Mishra GD, Woodward M. Risk of all-cause mortality and vascular events in women versus men with type 1 diabetes: A systematic review and meta-analysis. Lancet Diabetes Endocrinol. 2015 Mar 1;3(3):198–206.
52. Coskun T, Sloop KW, Loghin C, Alsina-Fernandez J, Urva S, Bokvist KB, et al. LY3298176, a novel dual GIP and GLP-1 receptor agonist for the treatment of type 2 diabetes mellitus: From discovery to clinical proof of concept. Mol Metab. 2018 Dec 1;18:3–14.
53. Eli Lilly and Company. MOUNJARO- tirzepatide injection, solution [Internet]. [cited 2024 Apr 11]. Available from: https://www.lilly.com/news/media/media-kits/mounjaro
54. Swiss Agency for Therapeutic Products. Swiss Public Assessment Report - MOUNJARO. [Internet]. [cited 2024 Apr 11]. Available from: https://www.swissmedic.ch/swissmedic/de/home/humanarzneimittel/authorisations/new-medicines/mounjaro_injektionsloesung_fertigpen_tirzepatidum.html.

Docusign Envelope ID: 11555059-DCA3-47FE-9701-55DBADEB3B9E

55. Tschöp M, Nogueiras R, Ahrén B. Gut hormone-based pharmacology: novel formulations and future possibilities for metabolic disease therapy. Diabetologia. 2023 Oct;66(10):1796-1808.
56. Baggio LL, Drucker DJ. Glucagon-like peptide-1 receptor co-agonists for treating metabolic disease. Mol Metab. 2021 Apr: 46:101090.
57. Osinski C, Le Gléau L, Poitou C, de Toro-Martin J, Genser L, Fradet M, et al. Type 2 diabetes is associated with impaired jejunal enteroendocrine GLP-1 cell lineage in human obesity. Int J Obes. 2021 Jan 1;45(1):170–83.
58. Zibar K, Ćuća JK, Blaslov K, Bulum T, Smirčić-Duvnjak L. Difference in glucagon-like peptide-1 concentrations between C-peptide negative type 1 diabetes mellitus patients and healthy controls. Ann Clin Biochem. 2015 Mar 16;52(2):220–5.
59. Maida A, Hansotia T, Longuet C, Seino Y, Drucker DJ. Differential Importance of Glucose-Dependent Insulinotropic Polypeptide vs Glucagon-Like Peptide 1 Receptor Signaling for Beta Cell Survival in Mice. Gastroenterology. 2009;137(6):2146–57.
60. Yusta B, Baggio LL, Estall JL, Koehler JA, Holland DP, Li H, et al. GLP-1 receptor activation improves β cell function and survival following induction of endoplasmic reticulum stress. Cell Metab. 2006 Nov;4(5):391–406.
61. Finan B, Ma T, Ottaway N, Müller TD, Habegger KM, Heppner KM, et al. Unimolecular Dual Incretins Maximize Metabolic Benefits in Rodents, Monkeys, and Humans. Sci Transl Med. 2013 Oct 30;5(209):209ra151
62. Hammoud R, Drucker DJ. Beyond the pancreas: contrasting cardiometabolic actions of GIP and GLP1. Vol. 19, Nature Reviews Endocrinology. Nat Rev Endocrinol. 2023 Apr;19(4):201-216.
63. Samms RJ, Christe ME, Collins KAL, Pirro V, Droz BA, Holland AK, et al. GIPR agonism mediates weight-independent insulin sensitization by tirzepatide in obese mice. J Clin Invest. 2021 Jun 15;131(12):e146353.
64. El K, Douros JD, Willard FS, Novikoff A, Sargsyan A, Perez-Tilve D, et al. The incretin co-agonist tirzepatide requires GIPR for hormone secretion from human islets. Nat Metab. 2023 Jun 1;5(6):945–54.
65. Samms RJ, Zhang GF, He W, Ilkayeva O, Droz BA, Bauer SM, et al. Tirzepatide induces a thermogenic-like amino acid signature in brown adipose tissue. Mol Metab. 2022 Oct 1;64.
66. van Eenige R, Ying Z, Tramper N, Wiebing V, Siraj Z, de Boer JF, et al. Combined glucose-dependent insulinotropic polypeptide receptor and glucagon-like peptide-1 receptor agonism attenuates atherosclerosis severity in APOE*3-Leiden.CETP mice. Atherosclerosis. 2023 May 1;372:19–31.
67. Kahles F, Liberman A, Halim C, Rau M, Möllmann J, Mertens RW, et al. The incretin hormone GIP is upregulated in patients with atherosclerosis and stabilizes plaques in ApoE−/− mice by blocking monocyte/macrophage activation. Mol Metab. 2018 Aug 1;14:150–7.
68. Taktaz F, Scisciola L, Fontanella RA, Pesapane A, Ghosh P, Franzese M, et al. Evidence that tirzepatide protects against diabetes-related cardiac damages. Cardiovasc Diabetol. 2024 Dec 1;23(1).
69. Kannt A, Madsen AN, Kammermeier C, Elvert R, Klöckener T, Bossart M, et al. Incretin combination therapy for the treatment of non-alcoholic steatohepatitis. Diabetes Obes Metab. 2020 Aug 1;22(8):1328–38.
70. Min T, Bain SC. The Role of Tirzepatide, Dual GIP and GLP-1 Receptor Agonist, in the Management of Type 2 Diabetes: The SURPASS Clinical Trials. Vol. 12, Diabetes Therapy. Adis; 2021. p. 143–57.
71. le Roux CW, Zhang S, Aronne LJ, Kushner RF, Chao AM, Machineni S, et al. Tirzepatide for the treatment of obesity: Rationale and design of the SURMOUNT clinical development program. Obesity. 2023 Jan 1;31(1):96–110.
72. European Medicines Agency (EMA). Mounjaro [Internet]. [cited 2024 Apr 11]. Available from: https://www.ema.europa.eu/en/medicines/human/EPAR/mounjaro
73. Mounjaro-Tirzepatide [Internet]. [cited 2024 May 8]. Available from: https://dailymed.nlm.nih.gov/dailymed/drugInfo.cfm?setid=d2d7da5d-ad07-4228-955f-cf7e355c8cc0
74. Heise T, Mari A, DeVries JH, Urva S, Li J, Pratt EJ, et al. Effects of subcutaneous tirzepatide versus placebo or semaglutide on pancreatic islet function and insulin sensitivity in adults with

type 2 diabetes: a multicentre, randomised, double-blind, parallel-arm, phase 1 clinical trial. Lancet Diabetes Endocrinol. 2022 Jun 1;10(6):418–29.

75. Thomas MK, Nikooienejad A, Bray R, Cui X, Wilson J, Duffin K, et al. Dual GIP and GLP-1 Receptor Agonist Tirzepatide Improves Beta-cell Function and Insulin Sensitivity in Type 2 Diabetes. J Clin Endocrinol Metab. 2021 Jan 23;106(2):388-396.

76. Pedersen SD, Giorgino F, Umpierrez G, Thieu VT, Rodríguez A, Nicolay C, et al. Relationship between body weight change and glycaemic control with tirzepatide treatment in people with type 2 diabetes: A post hoc assessment of the SURPASS clinical trial programme. Diabetes Obes Metab. 2023 Sep 1;25(9):2553–60.

77. Nauck MA, D'Alessio DA. Tirzepatide, a dual GIP/GLP-1 receptor co-agonist for the treatment of type 2 diabetes with unmatched effectiveness regrading glycaemic control and body weight reduction. Cardiovasc Diabetol. 2022 Sep 1;21(1):169.

78. Wong E, Cope R, Dima L, Nguyen T. Tirzepatide: A Dual Glucose-dependent Insulinotropic Polypeptide and Glucagon-Like Peptide-1 Agonist for the Management of Type 2 Diabetes Mellitus. Am J Ther. 2023 Jan-Feb;30(1):e26-e35.

79. Viljoen A, Pantalone KM, Galindo RJ, Cui X, Huh R, Hemmingway A, et al. Time to Reach Glycaemic and Body Weight Loss Thresholds with Tirzepatide in Patients with Type 2 Diabetes: A Pre-planned Exploratory Analysis of SURPASS-2 and SURPASS-3. Diabetes Therapy. 2023 May 1;14(5):925–36.

80. 6. Glycemic Goals and Hypoglycemia: Standards of Care in Diabetes—2024. Diabetes Care. 2024 Jan 1;47:S111–25.

81. Karagiannis T, Avgerinos I, Liakos A, Prato S Del, Matthews DR, Tsapas A, et al. Management of type 2 diabetes with the dual GIP/GLP-1 receptor agonist tirzepatide: a systematic review and meta-analysis. Diabetologia. 2022 Aug;65(8):1251-1261.

82. Frías JP, Davies MJ, Rosenstock J, Pérez Manghi FC, Fernández Landó L, Bergman BK, et al. Tirzepatide versus Semaglutide Once Weekly in Patients with Type 2 Diabetes. N Engl J Med. 2021 Aug 5;385(6):503-515

83. Schofield Jan Ho Handrean Soran J. Cardiovascular Risk in Type 1 Diabetes Mellitus. Diabetes Ther. 2019 Jun;10(3):773-789

84. Sattar N, McGuire DK, Pavo I, Weerakkody GJ, Nishiyama H, Wiese RJ, et al. Tirzepatide cardiovascular event risk assessment: a pre-specified meta-analysis. Nat Med. 2022 Mar 1;28(3):591–8.

85. Nicholls SJ, Bhatt DL, Buse JB, Prato S Del, Kahn SE, Lincoff AM, et al. Comparison of tirzepatide and dulaglutide on major adverse cardiovascular events in participants with type 2 diabetes and atherosclerotic cardiovascular disease: SURPASS-CVOT design and baseline characteristics. Am Heart J. 2024 Jan 1;267:1–11.

86. Jørgensen ME, Almdal TP, Carstensen B. Time trends in mortality rates in type 1 diabetes from 2002 to 2011. Diabetologia. 2013 Nov;56(11):2401–4.

87. Pharmacologic Approaches to Glycemic Treatment: Standards of Care in Diabetes—2024. Diabetes Care. 2024 Jan 1;47:S158–78.

88. Weinstock RS, Xing D, Maahs DM, Michels A, Rickels MR, Peters AL, et al. Severe hypoglycemia and diabetic ketoacidosis in adults with type 1 diabetes: Results from the T1D exchange clinic registry. J Clin Endocrinol Metab. 2013 Aug;98(8):3411-9.

89. Tong K, Yin S, Yu Y, Yang X, Hu G, Zhang F, et al. Gastrointestinal adverse events of tirzepatide in the treatment of type 2 diabetes mellitus A meta-analysis and trials sequential analysis. Medicine (Baltimore). 2023 Oct 27;102(43):e35488

90. Cai W, Zhang R, Yao Y, Wu Q, Zhang J. Tirzepatide as a novel effective and safe strategy for treating obesity: a systematic review and meta-analysis of randomized controlled trials. Front Public Health. 2024 Jan 31:12:1277113.

91. France NL, Syed YY. Tirzepatide: A Review in Type 2 Diabetes. Drugs. 2024 Feb;84(2):227-238.

92. Zeng Q, Xu J, Mu X, Shi Y, Fan H, Li S. Safety issues of tirzepatide (pancreatitis and gallbladder or biliary disease) in type 2 diabetes and obesity: a systematic review and meta-analysis. Front Endocrinol (Lausanne). 2023 Oct 16:14:1214334.

93. Yang Y, He L, Liu P, Wang J, Yang N, Li Z, et al. Impact of a dual glucose-dependent insulinotropic peptide/glucagon-like peptide-1 receptor agonist tirzepatide on heart rate among

Docusign Envelope ID: 11555059-DCA3-47FE-9701-55DBADEB3B9E

patients with type 2 diabetes: A systematic review and pairwise and network meta-analysis. Diabetes Obes Metab. 2024 Feb 1;26(2):548–56.

94. Umpierrez G, Korytkowski M. Diabetic emergencies-ketoacidosis, hyperglycaemic hyperosmolar state and hypoglycaemia. Nat Rev Endocrinol. 2016 Apr;12(4):222-32.

95. Fosbøl MO, Zerahn B. Contemporary methods of body composition measurement. Clin Physiol Funct Imaging. 2015 Mar;35(2):81-97.

96. Ward LC. Bioelectrical impedance analysis for body composition assessment: reflections on accuracy, clinical utility, and standardisation. Eur J Clin Nutr. 2019 Feb;73(2):194-199.

97. Zhang YN, Fowler KJ, Ozturk A, Potu CK, Louie AL, Montes V, et al. Liver fibrosis imaging: A clinical review of ultrasound and magnetic resonance elastography. J Magn Reson Imaging. 2020 Jan;51(1):25-42.

98. Herzig D, Nakas CT, Stalder J, Kosinski C, Laesser C, Dehais J, et al. Volumetric food quantification using computer vision on a depth-sensing smartphone: Preclinical study. JMIR Mhealth Uhealth. 2020 Mar 25;8(3):e15294

99. Michels WM, Grootendorst DC, Verduijn M, Elliott EG, Dekker FW, Krediet RT. Performance of the Cockcroft-Gault, MDRD, and new CKD-EPI formulas in relation to GFR, age, and body size. Clin J Am Soc Nephrol. 2010 Jun;5(6):1003-9.

100. Scott NW, McPherson GC, Ramsay CR, Campbell MK. The method of minimization for allocation to clinical trials: A review. Control Clin Trials. 2002;23(6):662–74.

101. World Health Organization. [Internet. 2021 [cited 2024 Apr 11]. Available from: https://cdn.who.int/media/docs/default-source/ncds/ncd-surveillance/steps/part3-section5.pdf?sfvrsn=a46653c7_2. Noncommunicable Disease Surveillance, Monitoring and Reporting.

102. Jin B, Lin H, Yuan J, Dong G, Huang K, Wu W, et al. Abdominal Adiposity and Total Body Fat as Predictors of Cardiometabolic Health in Children and Adolescents with Obesity. Front Endocrinol (Lausanne). 2020 Sep 4;11.

103. Chung JY, Kang HT, Lee DC, Lee HR, Lee YJ. Body composition and its association with cardiometabolic risk factors in the elderly: A focus on sarcopenic obesity. Arch Gerontol Geriatr. 2013;56(1):270–8.

104. Banks PA, Bollen TL, Dervenis C, Gooszen HG, Johnson CD, Sarr MG, et al. Classification of acute pancreatitis - 2012: Revision of the Atlanta classification and definitions by international consensus. Gut. 2013 Jan;62(1):102–11.

Annex 1. Guidance on the functionality of the automatic insulin delivery systems (AID).

Insulin delivery may be reduced by 20-30% during tirzepatide initiation by adjusting the specific AID modifiable parameters (carbohydrate–insulin ratio, correction factor, active insulin time or target glucose, etc) (**See Annexes 1-2**).

The study physician will be updated on the basic functionality of commercial AID systems, their adjustable settings, and troubleshooting per manufacturer's instructions. For further information please **see Table A1** and https://www.pantherprogram.org/device-comparison-chart

Table A1

	**Medtronic 780G**	**Medtronic 780G**	**Tandem Control IQ**	**YpsoPump**
**How it works**	Automated basal insulin delivery every 5 minutes based on total daily dose from the past 2-6 days.	"Auto Basal" calculated from total daily insulin, which is updated each day at midnight. Auto Basal is adjusted every 5 min based on recent CGM glucose trends, aiming for the target glucose value.	Automated basal insulin delivery that increases or decreases the patient's programmed basal rate based on CGM glucose trends	The algorithm works by auto-calculating basal rates and correction factors and delivers as an extended bolus every 8-12 minutes
**Algorithm target glucose level/range**	6.7 mmol/L (120 mg/dL) (fixed)	3 target options: 5.5, 6.1, 6.7 mmol/L (100, 110, 120 mg/dL)	6.25-8.9 mmol/L (112.5-160 mg/dL)(fixed day-time)<br>6.2 - 6.7 mmol/L(112-120 mg/dL) (fixed night-time)	Default target: 5.8 mmol/L (104 mg/dL).<br>Customizable personal target: 4.4 -11.0 mmol/dL (80-198 mg/dL)
**What can be adjusted? (modifiable settings in AID)**	Insulin: Carb ratio.<br>Active insulin time.	Insulin: Carb ratio.<br>Active insulin time.<br>Auto-correction switch.	Basal rate.<br>Insulin: Carb ratio.<br>Correction factor.	Target system glucose.<br>Insulin: Carb ratio.<br>"Boost" or "Ease off mode"—more or less aggressive algorithm.
**Auto bolus feature**	No	Yes, auto-correction boluses (max. every 5 min) if glucose is >6.7mmol/L (120 mg/dL).<br><br>Auto corrections can be turned on or off.	Yes, automated boluses every 1 hour (60% of calculated bolus) if needed.<br>If the patient takes a bolus by him/herself, the auto bolus clock resets to 60 minutes from the	Standard, extended, combination, and blind bolus.<br>Stacked bolus possible.

			patient's manual bolus.	
**Additional features on closed-loop mode**	Temp target (during exercise or stress): increases the glucose target to 8.3 mmol/L (150 mg/dL).	Exercise: Temporary Target can turn on 1-2 hours before starting exercise and consider leaving it on for several hours after exercise ends if delayed hypoglycemia is a concern.<br><br>Sleep: Can adjust Target as needed (only 1 target setting for 24-hr period). Could also consider the use of Temp Target if hypoglycemia is occurring during sleep (will disable auto-correction boluses and raise auto basal target to 8.3 mmol/L [150 mg/dL]).	Exercise: Increase the glucose target to 7.8 mmol/L (140-160 mg/dL).<br><br>Sleep: changes the target to 6.25 mmol/L (112.5 mg/dL) to 6.7 mmol/L (120 mg/dL) but stops auto bolusing during this mode.	Exercise: Lower basal rate one or two hours before exercising ("Ease-off" mode reduces insulin delivery depending on glucose levels and stops insulin delivery if the glucose level is predicted to fall below target).

Annex 2. General Guidance on AID settings adjustment during drug up-titration.

Investigators will train the participants on diabetes self-management principles, including hypoglycemia recognition, prevention, and treatment. This includes training on hypoglycemia and hyperglycemia prevention and AID-related troubleshooting.

At randomization and for tirzepatide titration (four weeks), investigators will adjust the AID settings based on baseline glycemic control (time in range) and risk of hypoglycemia (time below range). A general guidance is provided below.

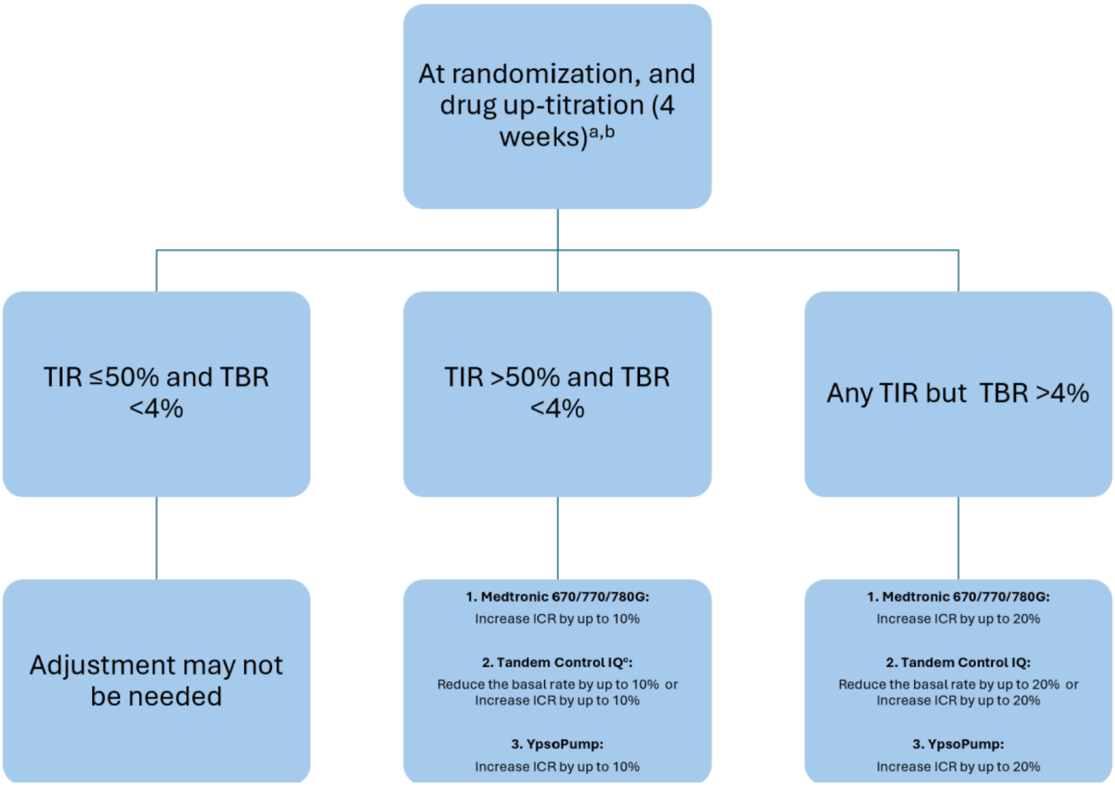

Abbreviations: TIR = percent time in range 3.9–10.0 mmol/L (70-180mg/dL); TBR = percent time below 3.9 mmol/L (70 mg/dL); ICR= insulin to carbohydrate ratio.

[a]Each participant with T1D is different in terms of response to GIP/GLP1Ra (tirzepatide) and risk of hypoglycemia. This general guidance does not replace the clinical judgment of the study physician (PI). The PI will adjust the settings to mitigate the risk of hypoglycemia based on the participant-individualized risk factors and clinical experience.

[b]Insulin reduction may depend on baseline A1c. People with higher HbA1c (A1c >8%) may require a 15 to 20% reduction in insulin dose while people with A1c close to 7% may require up to 30% reduction in insulin dose. Patients with higher A1c (> 9%) may not need any dose reduction, especially during the first month of adjunct treatment with GIP/GLP-1RA (tirzepatide).

[c]If needed, investigators can modify the correction factor (insulin sensitivity) accordingly in addition to changes in basal rate or ICR.

Moreover, investigators may recommend that participants use temporary manual mode or exercise mode/temporary target if the above changes are insufficient to mitigate hypoglycemia risk.

Annex 3. General Guidance on AID settings adjustments after tirzepatide titration.

The glycemic goal for most patients with T1D is TIR >70% and TBR <4%. Investigators are encouraged to review the AID settings and adjust if the participant is not within the glycemic goal.

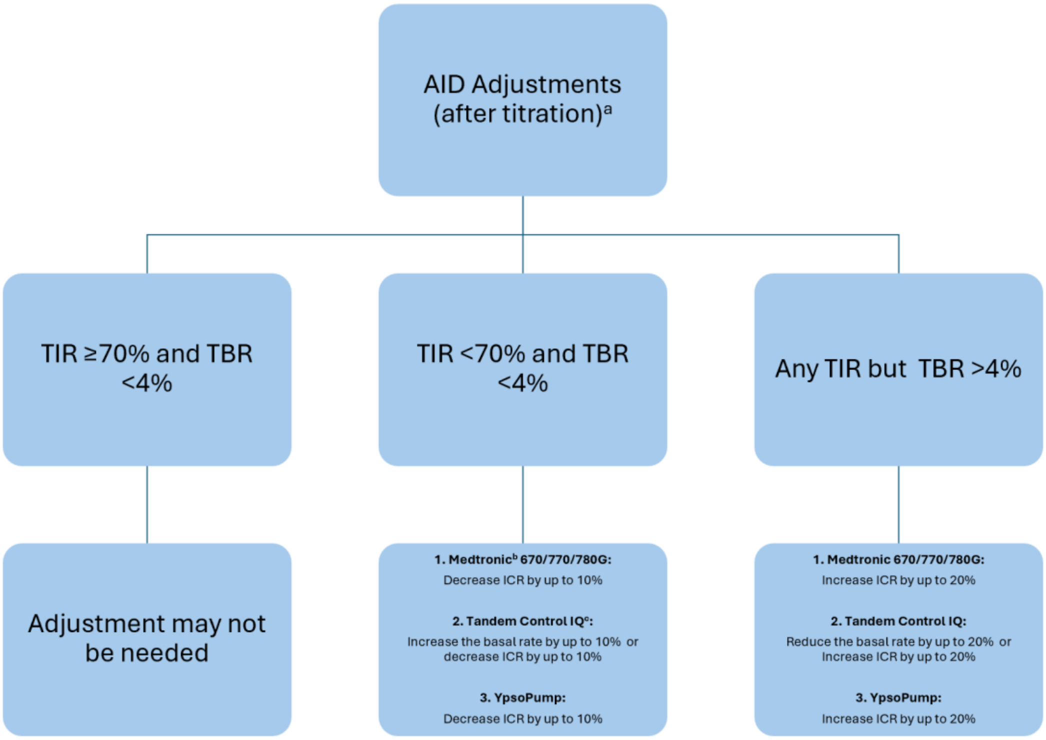

Abbreviations: TIR = percent time in range 3.9–10.0 mmol/L (70-180mg/dL); TBR = percent time below 3.9 mmol/L (70 mg/dL); ICR= insulin to carbohydrate ratio.

[a]Each participant with T1D is different in terms of response to GIP/GLP1RA (tirzepatide) and risk of hypoglycemia. This general guidance does not replace the clinical judgment of the study physician. The PI will adjust the AID settings to mitigate the risk of hypoglycemia based on the participant-individualized risk factors and clinical experience.

[b,c]If needed, investigators can modify the correction factor (insulin sensitivity) accordingly or active insulin time (for 780G) in addition to changes in basal rate or ICR.

Moreover, the PI may recommend participants to use manual mode or exercise mode/different target temporarily if the above changes are not sufficient to mitigate hypoglycemia risk.

Annex 4. General guidance on hypoglycemia management

Participants will be encouraged to follow the guidance provided in **Annexes 2 and 3** to adjust the AID settings during the drug up-titration and observation period to minimize the risk for hypoglycemia.

- Each participant must have glucagon products with them as a part of the standard of diabetes care. The participants and caregivers (if any) will be trained on using glucagon products.
- Participants should be advised to follow treatment for hypoglycemia. if glucose <3.9 mmol/L (70 mg/dL), take 15 grams of carbs (or less as AID may have insulin suspension for anticipated hypoglycemia) and repeat after 15 minutes until glucose is >3.9 mmol/L (70 mg/dL).
- If a participant experiences clinically significant hypoglycemia events or severe hypoglycemia, it should be reported as AE (if criteria are met according to the study protocol) or SAE respectively . The AID settings should be adjusted to prevent another episode of hypoglycemia.

Annex 5. Guidance on ketone monitoring

All participants will receive the above guidelines in their mother tongue (German) and the study team's contact information (name and phone) in case of emergency.

The device can also cause high blood sugar. Participants should contact the device's customer service to resolve any issues, such as the pump or CGM failure.

All participants must have a ketone meter

When to check for ketones

*Nausea/vomiting and unable to drink liquids for more than 1 hour

*Temperature >38°C

*If you are going to the Hospital for any reason

*If you are sick for any reason

*Blood glucose >16.6 mmol/L (300 mg/dL) for more than 2 hours or >22.2 mmol/L (400 mg/dL) at any point

Elevated Ketones

*If ketones **<0.6 mmol/L**, treat high blood glucose as you would normally treat

*If Ketones **<0.6-0.9 mmol/L**: make sure that the infusion site is in place. Change infusion site if blood glucose >13.9 mmol/L (250 mg/dL) for more than 2 hours. You may give insulin injection by insulin syringe or insulin pen till blood glucose is <10 mmol/L (180 mg/dL) (keep the pump off and restart once glucose is <10 mmol/L [180 mg/dL]).

*Drink water freely as much as you can

*Ketones: **1-1.5 mmol/L**: If you can drink liquids, please follow the guidelines mentioned above. Contact your study staff if you are unable to drink liquids.

*Ketones **>1.5 mmol/L**: call your study staff and go to the emergency room if you cannot drink liquids and are dehydrated.

Annex 6. Clinical Laboratory Test

**Clinical Laboratory Test**	
**Hematology**	**Pancreas (Exocrine) Panel**
Hemoglobin	Pancreatic amylase
Hematocrit	Lipase
**Hepatic Chemistry/Coagulation**	**Lipid Panel (Fasting)**
Total bilirubin	Total cholesterol
Direct bilirubin	LDL
Alkaline phosphatase	HDL
ALT	Triglycerides
AST	
GGT	**HbA1c**
PTT	
PT/INR	**Fasting Plasma Glucose**
Albumin	
	**Pregnancy test (serum or urine; females only)[c]**
**Endocrine**	
TSH	**Urine Chemistry**
FT4	Creatinine
Calcitonin	Urine albumin/creatinine ratio (uACR)
C-peptide (fasting)	
Estradiol[a]	
FSH[b]	

Abbreviations: FSH = follicle-stimulating hormone; HbA1c = hemoglobin A1c; HDL = high-density lipoprotein; LDL = low-density lipoprotein; RBC = red blood cells; VLDL = very low-density lipoprotein; WBC = white blood cells; ALT = alanine aminotransferase; AST = aspartate aminotransferase; CPK = creatinine phosphokinase; GGT = gamma-glutamyl transferase; PTT = Partial Thromboplastin Time; PT = Prothrombin Time; International Normalized Ratio (INR); TSH = Thyroid stimulating hormone; FT4 = free thyroxine.

[a,b]FSH test performed at Visit 1 (screening) for postmenopausal women of at least 45 years of age with an intact uterus, not on hormone therapy, and who have had spontaneous amenorrhea for more than six months and less than 12 months and estradiol levels consistent with a postmenopausal state (FSH ≥40 mIU/mL and estradiol <30 pg/mL).

[c]Serum pregnancy test (serum beta-hCG) will be performed during the screening visit. The result must be available before randomization and the first injection of the study drug for women of childbearing potential only. Additional pregnancy in urine tests will be performed during the study clinical visits.

[d]Urine albumin/creatinine ratio and estimated glomerular filtration rate (using the CKD-EPI formula) will be calculated by the central laboratory and included in laboratory result reports.

Annex 6.1 Clinical Laboratory Test

**Clinical Laboratory Test per visit**	
**Visit 1 – Screening**	**Visit 3 – Follow-up**
Hemoglobin	Total bilirubin
Hematocrit	Direct bilirubin
Total bilirubin	Indirect bilirubin
Direct bilirubin	ALT
Alkaline phosphatase	AST
ALT	Pancreatic amylase
AST	Serum Lipase
GGT	Creatinine
PTT	
PT/INR	**Visit 4 – Last Visit**
Serum Albumin	ALT
TSH	AST
FT4	Direct bilirubin
Calcitonin	Total bilirubin
C-peptide (fasting)	Indirect bilirubin
Estradiol	Calcitonin
FSH	Serum Pancreatic amylase
Serum Pancreatic amylase	Serum Lipase
Serum Lipase	Total cholesterol
Total cholesterol	LDL
LDL	HDL
HDL	Triglycerides
Triglycerides	Urine albumin/creatinine ratio (uACR)
HbA1c	HbA1c
Serum pregnancy test (serum beta-hCG)	
Urine albumin/creatinine ratio (uACR)	

Annex 7. Contraceptive Guidance and Collection of Pregnancy Information.

General definitions for contraceptive guidance can be found in **Table A2**.

Table A2. General Definitions

Word/Phrase	Definition
Women not of childbearing potential	Females are considered women not of childbearing potential:<br><br>(i) after menopause<br>(ii) before the menarche<br>(iii) if they have a congenital anomaly such as Mullerian agenesis with primary amenorrhea, **or**<br>(iv) who are infertile due to surgical sterilization.<br><br>Examples of surgical sterilization include:<br>(i) hysterectomy<br>(ii) bilateral oophorectomy, and<br>(iii) tubal ligation.
Post-menopausal state	The post-menopausal state is defined as:<br><br>(i) a woman at any age with at least six weeks post-surgical bilateral oophorectomy with or without hysterectomy, confirmed by operative note, **or**<br>(ii) a woman who is 40 to less than 55 years of age with an intact uterus, not on hormone therapy, who has cessation of menses for at least 12 months without an alternative medical cause, AND a follicle-stimulating hormone (FSH) >40 mIU/mL, **or**<br>(iii) a woman 55 years or older not on hormone therapy, who has had at least 12 months of spontaneous amenorrhea, **or**<br>(iv) a woman at least 55 years of age with a diagnosis of menopause before starting hormone replacement therapy.

Classification of Contraceptive Methods based on their Effectiveness.

Table A3. Classification of Contraceptive Methods based on their Effectiveness.

Effectiveness	Examples
Highly effective contraception	**ORAL**<br>- Combination oral contraceptive pills<br>- Mini pill<br>**NON-ORAL**<br>- Implanted contraceptives<br>- Injectable contraceptives<br>- Contraceptive patch (only women <90 kg)<br>- Total abstinence (if this is their preferred usual lifestyle)<br>- Vasectomy (partner)<br>- Fallopian tube implants (if confirmed by hysterosalpingogram)<br>- Combined contraceptive vaginal ring<br>- Intrauterine devices.
Effective contraception	- Male or female condoms with spermicide<br>- Diaphragms with spermicide or cervical sponges
Ineffective forms of contraception	- Barrier protection methods without the concomitant use of a spermicide<br>- Use of male and female condoms as a double barrier method due to the high failure rate when these methods are combined.<br>✓ Spermicide alone<br>✓ Immunocontraceptives<br>✓ Periodic abstinence<br>Fertility awareness or post-ovulation methods<br>✓ Calendar method.<br>✓ Temperature method.<br>✓ Combination of the above 2<br>✓ Cervical mucus.<br>✓ Symptothermal.<br>- Withdrawal<br>- Post-coital douche<br>- Lactational amenorrhea

Contraception Requirements for Female Participants

All females should follow the contraception guidance in Table A4 for the entirety of the study and 30 days thereafter.

Table A4. Contraception Requirements for Female Participants.

Category	Guidance
Women who remain abstinent as their preferred usual lifestyle	Remain abstinent
Women in exclusively same-sex relationships as their preferred and usual lifestyle	Are not required to use contraception
Women of non-childbearing potential	Are not required to use contraception
Women of childbearing potential (nonpregnant)	**MUST** test negative for pregnancy before initiation of treatment as indicated by a negative serum pregnancy test at the screening visit, followed by repeated urine pregnancy tests during the study clinical visits.<br><br>**AND**<br>Use one **NON-ORAL** form of highly effective contraception listed in **Table A3.**<br><br>If a female participant is using **ORAL** contraceptives. They **MUST** switch to a non-oral method (e.g., vaginal ring or hormonal patch) **OR** use a barrier method[a] for four weeks after starting tirzepatide for four weeks after each dose escalation[b]. Tirzepatide may reduce the effectiveness of oral hormonal contraceptives due to delayed gastric emptying, which is most pronounced after the first dose and diminishes over time.

[a]Only apply to female participants in the intervention group. Females in the control group with childbearing potential, who are using highly effective oral contraceptives, will continue their use without the need to switch for non-oral or additional barrier contraceptives.

[b]The study includes a 4-week titration phase followed by 12 weeks at target doses, participants must use a non-oral highly effective method of contraception or add a barrier method for eight weeks (four weeks after IMP initiation plus four weeks after target dose escalation). After this period, participants may either return to their previous oral contraceptives (if they switched) or discontinue using a barrier method they were using concurrently.

Contraception Guidance

Contraception Requirements for Male Participants in the Intervention Arm

All men in the intervention arm should refrain from sperm donation and follow the contraception guidance in **Table A5**. for the duration of the study and until 30 days following the last dose of the IMP.

Table A5. Contraception Requirements for Male Participants (Intervention Arm)

Category	Guidance
Men who remain abstinent as their preferred usual lifestyle	Remain abstinent
Men in exclusively same-sex relationships, as their preferred and usual lifestyle	Are not required to use contraception
Men with partners of childbearing potential (nonpregnant)	- Use condoms and the partner an additional highly effective method of contraception listed in **Table A3**.<br><br>**OR**<br><br>- Use condoms with spermicide and the partner an additional effective method of contraception such as diaphragms with spermicide, or cervical sponges
Men with partners of non-childbearing potential (postmenopausal females or females with tubal ligation hysterectomy or bilateral oophorectomy or have a congenital anomaly, for example, Mullerian agenesis)	Are not required to use contraception
Men who had a vasectomy or testicular removal surgery or who are sterile due to other medical conditions	Are not required to use contraception
Men with pregnant partners	Use condoms

Collection of Pregnancy Information

Male participants with partners who become pregnant.

- The investigator will attempt to collect pregnancy information on any male participant's female partner who becomes pregnant while the male participant is in this study. This applies only to male participants who receive study interventions.
- After obtaining the necessary signed informed consent from the pregnant female partner directly, the investigator will record pregnancy information on the appropriate form and submit it to the sponsor within 24 hours of learning of the partner's pregnancy. The female partner will also be followed to determine the outcome of the pregnancy.

Information on the status of the mother and child will be forwarded to the sponsor. Generally, the follow-up will be no longer than 6 to 8 weeks following the estimated delivery date. Any termination of the pregnancy will be reported regardless of fetal status (presence or absence of anomalies) or indication for the procedure.

Female participants who become pregnant

- The investigator will collect pregnancy information on any female participant who becomes pregnant while participating in this study. The initial information will be recorded on the appropriate form and submitted to the sponsor within 24 hours of learning of a participant's pregnancy.
- The participant will be followed to determine the outcome of the pregnancy. The investigator will collect follow-up information on the participant and neonate, which will be forwarded to the sponsor. Generally, follow-up will not be required for longer than 6 to 8 weeks beyond the estimated delivery date. Any termination of pregnancy will be reported, regardless of fetal status (presence or absence of anomalies) or indication for the procedure.
- While pregnancy itself is not considered to be an AE or SAE, any pregnancy complication or elective termination of a pregnancy for medical reasons will be reported as an AE or SAE.
- A spontaneous abortion (occurring at <22 weeks gestational age) or stillbirth (occurring at >20 weeks gestational age) is always considered to be an SAE and will be reported as such.
- Any post-study pregnancy-related SAE considered reasonably related to the study intervention by the investigator will be reported to the sponsor as described in **Section 10.1.2.** While the investigator is not obligated to actively seek this information from former study participants, he or she may learn of an SAE through spontaneous reporting.
- Any female participant who becomes pregnant while participating in the study will discontinue study intervention and be withdrawn from the study.

Annex 8. Mitigation plan for gastrointestinal symptoms

To mitigate GI symptoms and manage patients with intolerable gastrointestinal (GI) AEs during the escalation period (Weeks 0 to 8), the investigator should:

- Advise patients to eat smaller meals, such as splitting three daily meals into four or smaller meals and stop eating when they feel full.
- Prescribe symptomatic medication (for example, anti-emetic or antidiarrheal medication) per local availability and individual patient needs.
- Symptomatic medication will be registered as concomitant medication in the eCRF.
- Temporarily interrupt tirzepatide (omit one dose; the patient will take three of four doses at that dose level). After the interruption, restart at the same dose with the patient taking medication to alleviate their GI symptoms.
- The data related to the temporary interruption of study treatment will be documented in source documents and entered on the eCRF.

In case of intolerable GI symptoms or events persist despite the above measures, the investigator may decide to discontinue the study drugs or make a de-escalation back of the study drug. However, all patients will be encouraged to use a maximally tolerable study dose of up to 5 mg SC per week.

Patients who permanently stop the study drug will continue participating in the study according to the protocol to collect all planned efficacy and safety measurements. In the event of intolerable persistent GI symptoms that occur after the escalation period (Week 8), the investigator should take the above measures to keep the patient on the study.

Annex 9. Liver Safety: Hepatic Evaluation testing.

Refer to **Section 10.1.2**. for guidance on appropriate definitions and follow-up assessments.

- The appropriate liver tests (beyond ALT, AST, TB, Indirect and direct bilirubin) will be selected, as needed, according to the investigators' clinical criteria.
- The test will be performed by a central laboratory, and the results will be included in the CFRs.

**Hepatic Monitoring Tests**	
**Hepatic Hematology**	**Hepatic Coagulation**
Hemoglobin	Prothrombin Time
Hematocrit	Prothrombin Time, INR
RBC	
WBC	**Hepatic Serologies**
Neutrophils, segmented	Hepatitis A antibody, total
Lymphocytes Hepatic Serologies	Hepatitis A antibody, IgM
Monocytes	Hepatitis B surface antigen
Eosinophils	Hepatitis B surface antibody
Basophils	Hepatitis B Core antibody
Platelets	Hepatitis C antibody
	Hepatitis E antibody, IgG
**Hepatic Chemistry**	Hepatitis E antibody, IgM
Total bilirubin (TB)	
Direct bilirubin	**Other serologies**
Alkaline phosphatase	Anti-nuclear antibody (ANA)
ALT	Anti-smooth muscle antibody (ASMA)
AST	Anti-actin antibody
GGT	Epstein-Barr Virus (EBV) antibody
CPK	Epstein-Barr Virus DNA
	Cytomegalovirus antibody
**Other Chemistry**	Cytomegalovirus DNA
Acetaminophen	Herpes Simplex Virus (HSV)
Acetaminophen protein adducts	HSV (Type 1 and 2) antibody
Alkaline phosphatase isoenzymes	HSV (Type 1 and 2) DNA
Ceruloplasmin	LKM-1 Antibody
Copper	
Ethyl Alcohol	
Haptoglobin	

Immunoglobulin IgA (Quantitative)	
Immunoglobulin IgG (Quantitative)	
Immunoglobulin IgM (Quantitative)	
Phosphatidylethanol (Peth)	

Abbreviations: ALT = alanine aminotransferase; AST = aspirate aminotransferase; CPK = creatinine phosphokinase; GGT = gamma-glutamyl transferase; Ig = immunoglobulin; INR = international normalized ratio; RBC = red blood cells; WBC = white blood cells; LKM-1 = Liver Kidney Microsomal Type 1.

Annex 10

Table A6. Classification of Hypoglycemia

Classification of Hypoglycemia	Glycemic Criteria	Description
Hypoglycemia alert value (level 1)	< 3.9 mmol/L (70 mg/dL) and ≥ 3.0 mmol/L (54 mg/dL)	Sufficiently low for treatment with fast-acting carbohydrate and dose adjustment of glucose-lowering therapy
Clinically significant hypoglycemia (level 2)	< 3.0 mmol/L (54 mg/dL)[a]	Sufficiently low to indicate serious, clinically important hypoglycemia
Severe hypoglycemia (level 3)	No specific glucose threshold	Hypoglycemia associated with severe cognitive impairment requiring external assistance for recovery

Adapted from: Glycemic Goals and Hypoglycemia. ADA 2024 and the International Hypoglycemia Study Group. 2017.

[a]blood glucose must be below 3.0 mmol/L for at least 15 consecutive minutes.
