# Supplementary material for: GIP/GLP-1RA as adjunctive to automated insulin delivery in adults with Type 1 diabetes (the AID-JUNCT trial): Study protocol for a prospective, randomized, clinical trial
Source: PLoS One. 2025 Oct 22;20(10):e0335060. doi: 10.1371/journal.pone.0335060 (PMC12543135; doi:10.1371/journal.pone.0335060)
Supplement: Attachment — Submitted filename: Response to Reviewers.pdf [file pone.0335060.s006.pdf]

Study Title: GIP/GLP-1RA as Adjunctive to Automated Insulin Delivery in Adults with Type 1 Diabetes (The AID-JUNCT Trial): Study Protocol for a Prospective, Randomized, Clinical Trial.

Dear Reviewers,

We sincerely thank you for your thoughtful and constructive feedback on our manuscript. Below, we provide detailed responses to each of your comments. All suggested clarifications and revisions have been addressed in the revised manuscript, along with additional adjustments made in accordance with the journal's requirements.

We hope these revisions meet your expectations and enhance the overall quality and clarity of our work.

Reviewer #1: The authors plan to recruit 42 participants in a prospective, randomized controlled trial design to evaluate the safety and efficacy of adding tripeptide to insulin therapy in T2D participants.

1. **The sample size is calculated without considering the potential missing or dropout.**

Response: Thank you for your comment. The sample size was calculated considering a 20% dropout rate, which is common in most clinical trials. In addition, it is equal to or slightly higher than that proposed in similar trials with once-weekly GLP-1 receptor agonists (semaglutide) (doi:10.1056/EVIDoa2500173; doi:10.1038/s41591-024-03463-z). This information has been clarified in the updated manuscript (Lines 306-307, manuscript with track changes).

2. **Please comment on how to avoid the potential bias from both participants and investigators, as the open-label design is planned.**

Response: Thank you for this important point. As this is an open-label study, we recognize the potential risk for bias, specifically, the Hawthorne effect, performance bias, and attrition bias. Below are the strategies we are implementing to mitigate each:

- Hawthorne Effect: Both intervention and control groups will be observed equally. A run-in (baseline) period has been implemented to allow participants to acclimate to being observed before randomization. Additionally, the main study outcomes are automated via sensors to reduce observer influence.

- Performance Bias: All participants are followed under a standardized protocol. The study staff have been trained to ensure uniform care and consistent interaction with participants across groups. An external monitor is also overseeing that both arms are receiving care as intended.

- Attrition Bias: We are maintaining active follow-up through reminders and phone calls to encourage ongoing engagement. The study design includes remote visits to reduce participant burden by simplifying procedures and minimizing time/effort required. We also plan to use an intention-to-treat analysis, ensuring all participants are included in the final analysis according to their original group assignment. Dropout reasons will be tracked to identify any emerging patterns that can inform future studies.

The potential risks of bias have been discussed in more detail in the Discussion section of the revised manuscript (Lines 649-663, manuscript with track changes).

3. **It's unclear what imputation approach will be implement.**

Response: We plan to apply a multiple imputation approach with 10 imputations, as this is one of the most robust and widely recommended methods for handling missing data in clinical trials. This information has now been included in the methods section (Lines 490-491, manuscript with track changes).

4. **The ANCOVA and MMRM will be implemented with multiple covariates (e.g., hba1c, bmi, tir, group). With limited sample size, the robustness of the analysis results might be questionable.**

Response: Thank you for your valuable comment. We acknowledge that including multiple covariates in ANCOVA and MMRM models with a limited sample size raises concerns regarding model stability and overfitting. To mitigate this, we have limited covariates to those that are clinically relevant and pre-specified (baseline HbA1c, BMI, and Time in Range; TIR) to preserve degrees of freedom and avoid overfitting. Additionally, we will perform sensitivity analyses to test the robustness of the results under alternative model specifications. This approach seeks to balance confounding control with model stability while maintaining transparency and interpretability.
This information has been added to the methods section (Lines 479-481, manuscript with track changes).

5. **Is there any plan to compare the baseline characteristics for two arms?**

Response: Yes, a table comparing baseline characteristics between the two arms will be included in the final analysis and reported in the publication of the trial results.

Reviewer #2:

Sample Size Justification:

1. The sample size calculation (n=42) assumes a 10.5% difference in time in range (TIR) between groups. Please provide references supporting this effect size estimate for tirzepatide in T1D, as most cited references appear to be from type 2 diabetes studies.

Response: Thank you for the valuable comment. The sample size estimation was based on previous proof-of-concept studies with tirzepatide in T1D, as referenced in the manuscript (Lines 307-308, manuscript with track changes). While there are currently no results from full-scale clinical trials with tirzepatide in T1D, recent data on semaglutide, a once-weekly GLP-1 receptor agonist (doi:10.1056/EVIDoa2500173; doi:10.1038/s41591-024-03463-z) demonstrated an effect size in TIR of +8.8 percentage points (95% CI: 3.9 to 13.7) and +4.8 percentage points (SD: 7.6), which also supports our assumption. Given tirzepatide's dual incretin mechanism, we expect the effect size may be even greater.

2. Consider whether this sample provides adequate power for secondary outcomes like hypoglycemia rates or cardiometabolic parameters.

Response: Thank you for the comment. The sample size and power were calculated based solely on the primary outcome (Time in Range; TIR). We acknowledge that this sample size may not provide sufficient power for secondary or exploratory outcomes. However, these secondary outcomes are labeled as such since they are intended to support the interpretation of the primary outcome and generate hypotheses for future studies, as well as the exploratory outcomes. This has now been clarified in the discussion section (under study limitations; lines 669-673, manuscript with track changes).

Open-Label Design:

1.The potential for bias (performance, detection, and reporting bias) should be discussed in greater depth.

Response: Thank you for your suggestion. The potential for bias introduced by the open-label design is now discussed more thoroughly in the Discussion section of the revised manuscript (Lines 649-663, manuscript with track changes).

2.Consider adding blinded outcome adjudication for key endpoints like hypoglycemia and DKA events.

Response: Thank you for the suggestion. We acknowledge that blinded outcome adjudication enhances objectivity in classifying key events. While this is not currently part of our protocol, severe hypoglycemia and DKA are reviewed by the Clinical Safety Officer as part of safety monitoring. However, we recognize that this role is not blinded and does not constitute formal adjudication. We will consider implementing blinded adjudication for these endpoints in the final analysis, particularly if the frequency or clinical significance of such events warrants it or in future studies to improve outcome assessment robustness.

Statistical Analysis:

1.The plan to use ANCOVA for primary analysis is appropriate, but please specify how missing data will be handled (e.g., multiple imputation methods).

Response: We plan to apply a multiple imputation approach with 10 imputations, as this is one of the most robust and widely recommended methods for addressing missing data in clinical trials. This detail has been added to the Methods section (Lines 490-491, manuscript with track changes).

2.Consider adding sensitivity analyses accounting for potential confounding by baseline characteristics.

Response: We will perform sensitivity analyses to assess the robustness of findings under alternative model specifications and potential baseline confounding. This has been included in the appropriate section of the manuscript (Lines 479-481, manuscript with track changes).
