# Supplementary material for: GIP/GLP-1RA as adjunctive to automated insulin delivery in adults with Type 1 diabetes (the AID-JUNCT trial): Study protocol for a prospective, randomized, clinical trial
Source: PLoS One. 2025 Oct 22;20(10):e0335060. doi: 10.1371/journal.pone.0335060 (PMC12543135; doi:10.1371/journal.pone.0335060)
Supplement: Attachment — Submitted filename: comments.docx [file pone.0335060.s004.docx]

**Sample Size Justification**:

1. The sample size calculation (n=42) assumes a 10.5% difference in time in range (TIR) between groups. Please provide references supporting this effect size estimate for tirzepatide in T1D, as most cited references appear to be from type 2 diabetes studies.
2. Consider whether this sample provides adequate power for secondary outcomes like hypoglycemia rates or cardiometabolic parameters.

**Open-Label Design**:

1. The potential for bias (performance, detection, and reporting bias) should be discussed in greater depth.
2. Consider adding blinded outcome adjudication for key endpoints like hypoglycemia and DKA events.

**statistical Analysis**:

1. The plan to use ANCOVA for primary analysis is appropriate, but please specify how missing data will be handled (e.g., multiple imputation methods).
2. Consider adding sensitivity analyses accounting for potential confounding by baseline characteristics.
